# Boosting the interfacial superionic conduction of halide solid electrolytes for all-solid-state batteries

**DOI:** 10.1038/s41467-023-38037-z

**Published:** 2023-04-28

**Authors:** Hiram Kwak, Jae-Seung Kim, Daseul Han, Jong Seok Kim, Juhyoun Park, Gihan Kwon, Seong-Min Bak, Unseon Heo, Changhyun Park, Hyun-Wook Lee, Kyung-Wan Nam, Dong-Hwa Seo, Yoon Seok Jung

**Affiliations:** 1 Department of Chemical and Biomolecular Engineering, Yonsei University, Seoul, 03722 South Korea; 2 School of Energy and Chemical Engineering, Ulsan National Institute of Science and Technology (UNIST), Ulsan, 44919 South Korea; 3 Department of Energy and Materials Engineering, Dongguk University, Seoul, 04620 South Korea; 4 National Synchrotron Light Source II, Brookhaven National Laboratory, Upton, NY 11973 USA; 5 Department of Materials Science and Engineering, Yonsei University, 03722 Seoul, South Korea

**Keywords:** Batteries, Solid-state chemistry, Materials for energy and catalysis, Energy, Electrochemistry

## Abstract

Designing highly conductive and (electro)chemical stable inorganic solid electrolytes using cost-effective materials is crucial for developing all-solid-state batteries. Here, we report halide nanocomposite solid electrolytes (HNSEs) ZrO_2_(-ACl)-A_2_ZrCl_6_ (A = Li or Na) that demonstrate improved ionic conductivities at 30 °C, from 0.40 to 1.3 mS cm^−1^ and from 0.011 to 0.11 mS cm^−1^ for Li^+^ and Na^+^, respectively, compared to A_2_ZrCl_6_, and improved compatibility with sulfide solid electrolytes. The mechanochemical method employing Li_2_O for the HNSEs synthesis enables the formation of nanostructured networks that promote interfacial superionic conduction. Via density functional theory calculations combined with synchrotron X-ray and ^6^Li nuclear magnetic resonance measurements and analyses, we demonstrate that interfacial oxygen-substituted compounds are responsible for the boosted interfacial conduction mechanism. Compared to state-of-the-art Li_2_ZrCl_6_, the fluorinated ZrO_2_−2Li_2_ZrCl_5_F HNSE shows improved high-voltage stability and interfacial compatibility with Li_6_PS_5_Cl and layered lithium transition metal oxide-based positive electrodes without detrimentally affecting Li^+^ conductivity. We also report the assembly and testing of a Li-In||LiNi_0.88_Co_0.11_Mn_0.01_O_2_ all-solid-state lab-scale cell operating at 30 °C and 70 MPa and capable of delivering a specific discharge of 115 mAh g^−1^ after almost 2000 cycles at 400 mA g^−1^.

## Abstract (web summary)

Compositional tuning is a standard procedure to improve the ionic conductivity of inorganic superionic conductors. Here, the authors report (electro)chemical stable composite halide solid electrolytes applying a nanostructure approach that promotes interfacial superionic conductivity.

## Introduction

Lithium-ion batteries are ubiquitous in electronic devices, and now their use is expanding to electric vehicles^1–3^. However, concerns about their safety and limited energy density motivate the development of all-solid-state batteries (ASSBs) exploiting nonflammable inorganic superionic conductors to enable alternative electrode materials such as Li metal^2–12^.

Among various inorganic solid electrolyte (SE) candidates, sulfides, such as argyrodite Li_6-y_PS_5-y_X_1+y_ (X = Cl, Br; y = 0.0–0.5; Li^+^ conductivities range 1–10 mS cm^−1^ at 25 °C)^13^, have the advantages of high ionic conductivities reaching those of standard non-aqueous liquid electrolyte solutions and deformability, which allows for practical cold-pressing fabrication of ASSB cells^2,4^. However, due to their low intrinsic electrochemical oxidative limits (~2.6 V vs. Li/Li^+^), uncoated 4 V class layered Ni-rich LiMO_2_ (M = Ni, Co, Mn, or Al mixture) positive electrode active materials with sulfide SEs exhibit unsatisfactory performance^14–16^. Compared to sulfides, the other major class of SEs, oxides, such as Li_7_La_3_Zr_2_O_12_ (max. 1.8 mS cm^−1^ at 27 °C)^5,17^, have good (electro)chemical oxidation stabilities but are brittle, which makes it challenging to fabricate ASSBs without hot-sintering or hybridization with non-aqueous liquid electrolyte solutions^18,19^.

Recently, halide SEs have emerged as a strong contender because they have a balance and combination of the advantages of sulfides and oxides, i.e., mechanical sinterability with good (electro)chemical stability^20,21^. In 2018, Asano et al. reported that mechanochemically prepared trigonal Li_3_YCl_6_ exhibited moderate Li^+^ conductivity of 0.51 mS cm^−1^ at 25 °C and good performance in ASSB cells, even when uncoated LiCoO_2_-based positive electrode was used in combination with a Li-In negative electrode^20^. These results boosted research to develop halide SEs, such as Li_3_InCl_6_ (1.5 mS cm^−1^ at 25 °C), Li_3_ScCl_6_ (3 mS cm^−1^ at 25 °C), Li_2_Sc_2/3_Cl_4_ (1.5 mS cm^−1^ at 30 °C), Li_2_ZrCl_6_ (0.40 mS cm^−1^ at 30 °C), and Li_3_YbCl_6_ (0.19 mS cm^−1^ at 30 °C)^22–26^. Similar to other types of SE materials^2,4,6,13,27–29^, the conventional strategy of compositional tuning, e.g., aliovalent substitution, to control the charge carrier concentration and/or structural framework was applied to enhance the ionic conductivity of halide SEs:^22,26,30,31^ Li_3-x_M_1-x_Zr_x_Cl_6_ (M = Y, Er, max. 1.4 mS cm^−1^ at 25 °C), Li_3-x_M_1-x_Zr_x_Cl_6_ (M = In, Sc, max. 2.1 mS cm^−1^ at 30 °C), Li_2+x_Zr_1-x_M_x_Cl_6_ (M = Fe, Cr, V, max. 1.0 mS cm^−1^ at 30 °C), and Li_3-x_Yb_1-x_M_x_Cl_6_ (M = Zr, Hf, max. 1.5 mS cm^−1^ at 30 °C). Na^+^ halide analogues such as Na_3-x_Er_1-x_Zr_x_Cl_6_ and Na_3-x_Y_1-x_Zr_x_Cl_6_ were also developed via aliovalent substitution, but their conductivities were as low as 0.040 mS cm^−1^ at 25 °C and 0.066 mS cm^−1^ at 20 °C, respectively (vs. 0.018 mS cm^−1^ of Na_2_ZrCl_6_ at 30 °C)^32–34^. In addition, structural disorders, such as M (M = Y, Er) and/or Li^+^ site disorder and stacking faults, varied depending on the preparation protocol and were identified as the key factors for enhancing the ionic conductivity of halide SEs^26,30,33,35^.

In terms of practical applications, most halide SEs exploit scarce and expensive central metals, such as Y, Sc, and In, with the sole exception of Zr (Supplementary Fig. 1)^21,26,36^. Recent theoretical and experimental studies reported that the central metal cation and halide anion governed the electrochemical stability of halide SEs^37^. In particular, F-substitution in chloride SEs effectively pushes the electrochemical oxidative limit further^37^ but at the expense of lower ionic conductivities^37,38^. In contrast to their good electrochemical oxidation stability, halide SEs suffer from poor cathodic stability associated with the reduction of central metal cations^14,24,38,39^. In this regard, an ASSB design, wherein the halide and sulfide SEs function synergistically as the catholyte and SE layer that is placed in-between negative and positive electrodes (hereafter, referred to as SE layer), respectively, is reasonable for practical ASSBs^7,20–26,30–32,38,40^. However, the compatibility issue of halide/sulfide has been overlooked thus far^41^.

Since 1973, when Liang et al. discovered that the ionic conductivity of LiI improved from 10^−7^ to 10^−5^ S cm^−1^ at 25 °C with the addition of Al_2_O_3_, the ionic conduction enhancement in heterostructured systems has been an intriguing but debatable question for various material systems, such as CaF_2_/BaF_2_ multilayered films, LiF/silica films, LiBH_4_/Al_2_O_3_, and polymer electrolytes with inorganic fillers^42–48^. Because there is no clear understanding of such behaviour, the design principle for interfacial conduction enhancement has not been established. Therefore, it is critical to precisely understand the interfacial conduction mechanism to utilize the superionic conduction effect as a general material design principle. Specifically, application of the interfacial conduction strategy is not reported yet for any superionic conductor with ionic conductivity ≥1 mS cm^−1^ at 25 °C, which is the minimum conductivity for practical ASSBs^49^.

In this work, we report the mechanochemical preparation of Li^+^- and Na^+^-conducting halide nanocomposite SEs (HNSEs, e.g., ZrO_2_-AX-A_2_ZrX_6_, A = Li or Na, X = Cl, F), that exhibit enhancements in not only ionic conductivities via interfacial superionic conduction (Li_2_ZrCl_6_: from 0.40 to 1.3 mS cm^−1^ at 30 °C, Na_2_ZrCl_6_: 0.011 to 0.11 mS cm^−1^ at 30 °C, hereafter, all reported conductivity values, in the absence of temperature information, are to be understood as having been obtained at 30 °C) but also compatibility with sulfide SEs. Density functional theory (DFT) calculations revealed the underlying interfacial superionic behaviour by establishing the interfacial conduction principles of HNSEs. These were experimentally probed by combined synchrotron-based X-ray and ^6^Li magic-angle spinning–nuclear magnetic resonance (MAS-NMR) measurements and analyses. In addition, the HNSE strategy was applied to F-substituted Li_2_ZrCl_6_, which offset the degradation in ionic conductivity and resolved the incompatibility issue with sulfide Li_6_PS_5_Cl (LPSCl) at elevated temperature. These HNSEs enabled good electrochemical energy storage performances of lab-scale cells with Li-In negative electrodes and LiCoO_2_ (LCO) or single-crystalline LiNi_0.88_Co_0.11_Mn_0.01_O_2_ (S-NCM88) positive electrodes in terms of LPSCl compatibility at 60 °C, high-voltage stability, fast charging, and long-term cycle life.

## Results and discussion

### Synthesis and characterization of HNSEs

**Fig. 1:**
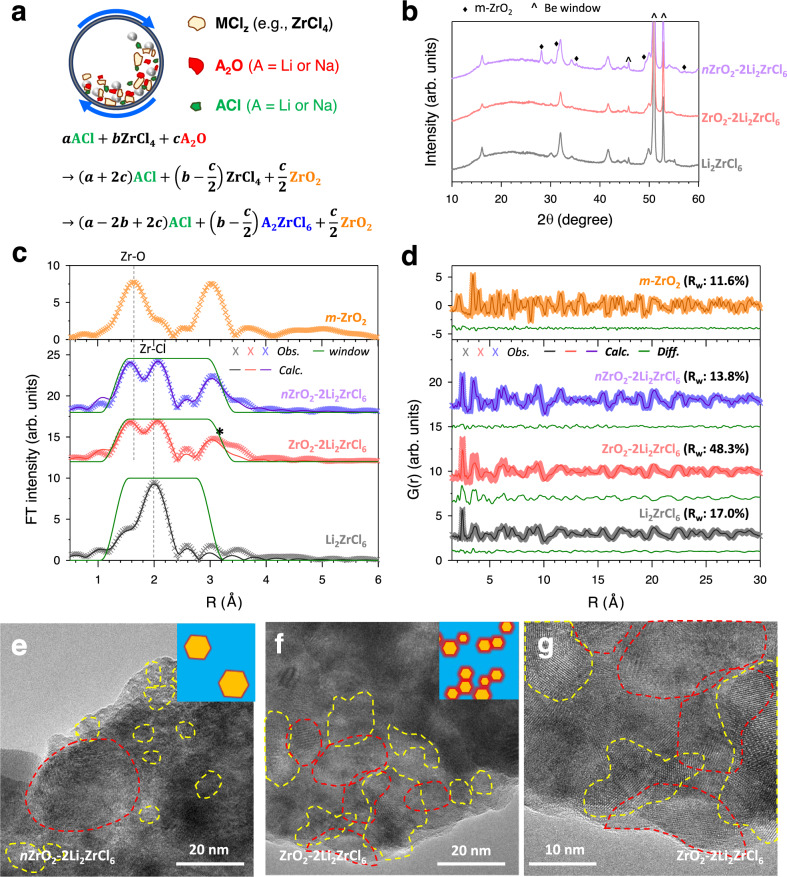
Synthesis and characterization of HNSEs (ZrO_2_−2Li_2_ZrCl_6_). **a** Schematic of the one-pot mechanochemical synthesis of ZrO_2_(-ACl)-A_2_ZrCl_6_ (A = Li or Na) HNSEs. **b**–**d** XRD patterns (**b**), coordination-number-refined Zr K-edge EXAFS fitting curves (**c**), and PDF G(r) with best-fit (**d**) for Li_2_ZrCl_6_, ZrO_2_-2Li_2_ZrCl_6_, and nZrO_2_-2Li_2_ZrCl_6_. **e**–**g** Cryo-HRTEM images of nZrO_2_-2Li_2_ZrCl_6_ (**e**) and ZrO_2_-2Li_2_ZrCl_6_ at lower magnification (**f**) and higher magnification (**g**). The red and yellow outlines indicate the domains of ZrO_2_ and Li_2_ZrCl_6_, respectively. Schematics of the nanostructures (blue: Li_2_ZrCl_6_, yellow: ZrO_2_, red: interface or interphase) are shown in the insets of (**e**) and (**f**).

HNSEs were prepared by the mechanochemical reaction of LiCl (or NaCl) and ZrCl_4_ with Li_2_O (or Na_2_O) (Fig. 1a). Li_2_O acts as an oxygen source and reacts with ZrCl_4_ to form ZrO_2_ nanoparticles^50^, and the residual ZrCl_4_ and LiCl react to produce Li_2_ZrCl_6_. Based on DFT calculations, the reaction to generate ZrO_2_ and LiCl from ZrCl_4_ and Li_2_O has a strong driving force (Supplementary Table 1, Supplementary Equation 1, ΔE = −4.736 eV). Furthermore, there is another spontaneous reaction from reactants (Li_2_O, ZrCl_4_, LiCl) to products (Li_2_ZrCl_6_, ZrO_2_) when the molar ratios are stoichiometrically matched (Supplementary Table 1, Supplementary Equation 2, ΔE = −5.000 eV). Moreover, Li_2_ZrCl_6_ is the only stable phase in the ZrO_2_-ZrCl_4_-LiCl ternary region (Supplementary Fig. 2 and Supplementary Note 1). Consistently, the synchrotron X-ray diffraction (XRD) and pair distribution function (PDF) results for a precursor mixture of Li_2_O and ZrCl_4_ (2:3 molar ratio) during mechanochemical milling (Supplementary Figs. 3 and 4) confirm the mechanochemical synthesis of ZrO_2_−2Li_2_ZrCl_6_ HNSEs with a negligible amount of precursors or impurities when the milling time is ≥ ≈20 h (Supplementary Note 2). Thus, the products of the mechanochemical reaction of LiCl and ZrCl_4_ with Li_2_O are comprised of Li_2_ZrCl_6_, ZrO_2_, and LiCl, and their fractions are determined from the stoichiometric ratio of the precursors. We extensively characterized the ZrO_2_−2Li_2_ZrCl_6_ HNSE sample as it is a binary system and exhibited a much higher Li^+^ conductivity of 1.1 mS cm^−1^ than Li_2_ZrCl_6_ (0.40 mS cm^−1^), despite the 7.86 vol.% of ionically insulating ZrO_2_ (based on the chemical formula of ZrO_2_−2Li_2_ZrCl_6_). For comparison, a control sample was prepared by mechanochemical milling commercially available ZrO_2_ nanoparticles (~20 nm) with Li_2_ZrCl_6_, referred to as nZrO_2_−2Li_2_ZrCl_6_.

The XRD pattern of ZrO_2_−2Li_2_ZrCl_6_ is compared with those of Li_2_ZrCl_6_ and nZrO_2_−2Li_2_ZrCl_6_ in Fig. 1b. The main reflections for ZrO_2_−2Li_2_ZrCl_6_ matched those of Li_2_ZrCl_6_ with hexagonal close-packed (hcp) trigonal structure (space group *P*$\bar{3}$*m*1), and their breadth indicated low crystallinity^20,26^. However, the XRD signals of ZrO_2_ were not observed, suggesting nanosized grains with poor crystallinity. The local structures of the poorly crystalline ZrO_2_−2Li_2_ZrCl_6_ HNSE were characterized by X-ray absorption spectroscopy (XAS) and PDF measurements, and the corresponding results are compared with those of Li_2_ZrCl_6_ and nZrO_2_−2Li_2_ZrCl_6_ in Fig. 1c, d and Supplementary Fig. 5. Zr K-edge X-ray absorption near-edge structure (XANES) spectra for all three samples showed the main edge position at ~18020 eV (Supplementary Fig. 5a), confirming the tetravalent oxidation state of Zr^26^. A Zr K-edge extended X-ray absorption fine structure (EXAFS) spectrum of ZrO_2_−2Li_2_ZrCl_6_ exhibited a distinct peak at ~1.5 Å (Fig. 1c) corresponding to the Zr-O coordination in ZrO_2_^51^, proving the mechanochemically driven formation of nanosized ZrO_2_ via the reaction of ZrCl_4_ with Li_2_O. The EXAFS spectra of Li_2_ZrCl_6_, ZrO_2_−2Li_2_ZrCl_6_, and nZrO_2_−2Li_2_ZrCl_6_ also showed peaks at ~2 Å for the shortest Zr-Cl coordination, confirming the octahedral coordination of Zr (ZrCl_6_^2−^)^26^. However, the local structural environments of Zr-O and Zr-Cl in the ZrO_2_−2Li_2_ZrCl_6_ HNSE differ from those in single-phase ZrO_2_ and Li_2_ZrCl_6_. While the Zr-O bond length decreases, the average Zr-Cl bond length slightly increases (from 2.46 Å to 2.47 Å in Supplementary Tables 2, 3) in ZrO_2_−2Li_2_ZrCl_6_ HNSE compared with each of the ZrO_2_ and Li_2_ZrCl_6_ phases. This result implies that the two nano-scale phases mutually affect the local structure of each other at the interface region. Such shrinkage of the ZrO_2_ polyhedron and expansion of the Cl-containing polyhedron could enlarge the size of the Li^+^ transport channels in the ZrO_2_−2Li_2_ZrCl_6_ HNSE, as discussed below for the analysis of the DFT calculations. Furthermore, compared to nZrO_2_−2Li_2_ZrCl_6_, the ZrO_2_−2Li_2_ZrCl_6_ HNSE showed a broader peak at ~3 Å for Zr-Zr coordination (indicated by an asterisk in Fig. 1c). A twofold increase in the Debye-Waller factor (Supplementary Tables 2, 3) for such Zr-Zr bonding (4.3 × 10^−3^ Å^2^ for nZrO_2_−2Li_2_ZrCl_6_, 8.0 × 10^−3^ Å^2^ for ZrO_2_−2Li_2_ZrCl_6_ HNSE) also suggested the highly disordered nature of the HNSE owing to the formation of a higher number of interfaces than in nZrO_2_−2Li_2_ZrCl_6_. The refined coordination numbers (Supplementary Tables 2, 3) for Zr-O and Zr-Cl bonds in the HNSE were significantly less than the ideal values based on the model ZrO_2_ and Li_2_ZrCl_6_ structures (e.g., 7.0 to 4.5 in Zr-O and 6.0 to 5.5 in Zr-Cl), providing additional evidence for the formation of interphases. The Cl K-edge XANES spectra (Supplementary Fig. 5b) were almost identical for all three samples with no pre-edge signals, indicating the ionic characteristics of the Zr-Cl bond^26^.

The PDF G(r) values of Li_2_ZrCl_6_, ZrO_2_−2Li_2_ZrCl_6_, and nZrO_2_−2Li_2_ZrCl_6_ are shown in Fig. 1d. The refinement result for Li_2_ZrCl_6_ confirmed the hcp trigonal structure with the *P*$\bar{3}$*m*1 space group^26,33^. PDF G(r) for both ZrO_2_−2Li_2_ZrCl_6_ and nZrO_2_−2Li_2_ZrCl_6_ revealed signals for both trigonal Li_2_ZrCl_6_ and monoclinic ZrO_2_. Importantly, whereas the ZrO_2_ PDF signal for nZrO_2_−2Li_2_ZrCl_6_ was distinct up to ~10 Å, that for the ZrO_2_−2Li_2_ZrCl_6_ HNSE disappeared above ~5 Å. This result indicated that the mechanochemically derived ZrO_2_ of the HNSE exhibited much smaller grain sizes and/or higher disorder compared with nZrO_2_−2Li_2_ZrCl_6_^33^. Moreover, we performed PDF fitting analysis for ZrO_2_−2Li_2_ZrCl_6_ HNSE and nZrO_2_−2Li_2_ZrCl_6_ using the model crystal structures obtained by fits of the single-phase PDFs of Li_2_ZrCl_6_ and ZrO_2_. The fit of nZrO_2_−2Li_2_ZrCl_6_ converged and showed a reasonably decent fit (*R*_w_ = 13.8 %) using two model structures. However, the fit of ZrO_2_−2Li_2_ZrCl_6_ HNSE was unsuccessful, ending with an unacceptable *R*_*w*_ value of 48.3%, which suggested the necessity of including an additional (inter)phase to unambiguously describe its complicated structure. This result indirectly emphasized the formation of interphases, enough to affect the average structure of the ZrO_2_−2Li_2_ZrCl_6_ HNSE. Further refinements of the PDF using additional (inter)phases indicated by DFT calculations are discussed in the Interfacial Superionic Conduction of HNSEs section.

Cryogenic high-resolution transmission electron microscopy (cryo-HRTEM) images for nZrO_2_−2Li_2_ZrCl_6_ and ZrO_2_−2Li_2_ZrCl_6_ are displayed in Fig. 1e-g. The corresponding fast Fourier transform (FFT) images are provided in Supplementary Figs. 6 and 7. For nZrO_2_−2Li_2_ZrCl_6_, crystalline ZrO_2_ nanoparticles with sizes 20-50 nm were distributed in the glass-ceramic Li_2_ZrCl_6_ matrix (Fig. 1e). Interestingly, the cryo-HRTEM images of the ZrO_2_−2Li_2_ZrCl_6_ HNSE (Fig. 1f, g) showed that ZrO_2_ nanograins with <20 nm sizes formed a local percolating network nanostructure. Li_2_ZrCl_6_ nanograin domains <10 nm were embedded in the percolating network nanostructure, implying large-area percolating interfaces (Supplementary Note 3), which were critical for anomalous interfacial conduction^46,47^. The mechanochemical preparation of the HNSEs was also effective for the Na^+^ analogues, such as ZrO_2_−2Na_2_ZrCl_6_ and 0.13ZrO_2_−0.61NaCl-0.26Na_2_ZrCl_6_. The corresponding XRD, PDF, and HRTEM results are presented in Supplementary Figs. 8 and 9.

**Fig. 2:**
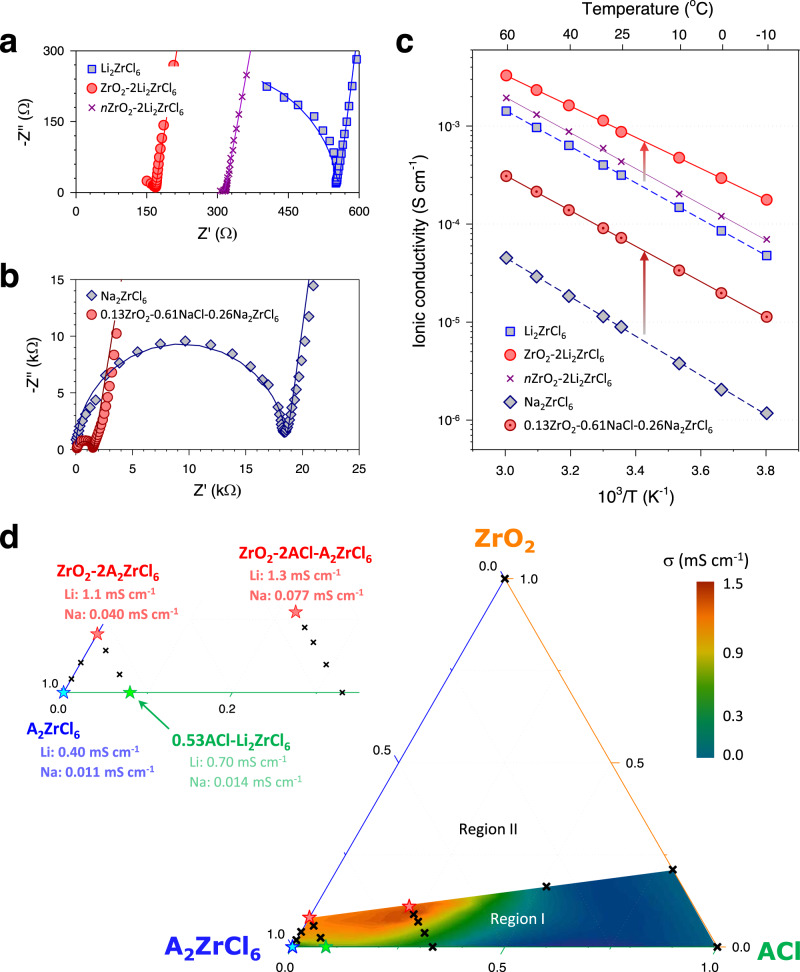
Ionic conductivities of Li^+^ and Na^+^ HNSEs (ZrO_2_(-ACl)-A_2_ZrCl_6_). **a**, **b** Nyquist plots of ion-blocking Ti|SE|Ti symmetric cells at 30 °C for Li^+^ HNSEs (ZrO_2_-2Li_2_ZrCl_6_ vs. Li_2_ZrCl_6_, nZrO_2_-2Li_2_ZrCl_6_) (**a**) and Na^+^ HNSE (0.13ZrO_2_-0.61NaCl-0.26Na_2_ZrCl_6_ vs. Na_2_ZrCl_6_) (**b**). The fitted lines using equivalent circuit model in Supplementary Fig. 10 with raw data (symbol) are also shown in (**a**) and (**b**). **c** Arrhenius plots of ionic conductivities for conventional halide SEs and HNSEs. **d** Ionic conductivity contour plot at 30 °C for ternary ZrO_2_-ACl-A_2_ZrCl_6_ HNSEs. The contour map was plotted using data represented by stars and crosses, with further details provided in Supplementary Tables 5 and 6.

The ionic conductivity results for the Li^+^ and Na^+^ HNSEs obtained by the AC impedance method using ion-blocking Ti|SE|Ti symmetric cells are shown in Fig. 2. Nyquist and Arrhenius plots for the ionic conductivity are displayed in Fig. 2a-c. The equivalent circuit model and the fitted results are also provided in Supplementary Fig. 10a and Supplementary Table 4. Compared to Li_2_ZrCl_6_, the ZrO_2_−2Li_2_ZrCl_6_ HNSE exhibited approximately threefold enhancement in the Li^+^ conductivity (1.1 vs. 0.40 mS cm^−1^) with a lowered activation energy (0.31 vs. 0.37 eV), which was noteworthy in that the ionically insulating ZrO_2_ occupied theoretically as much as 7.86 vol.% in ZrO_2_−2Li_2_ZrCl_6_, based on the chemical formula. Interestingly, the Li^+^ conductivity of the control sample, nZrO_2_−2Li_2_ZrCl_6_ (0.6 mS cm^−1^), was also slightly higher than that of Li_2_ZrCl_6_ but lower than that of the HNSE ZrO_2_−2Li_2_ZrCl_6_. Considering the smaller sizes of ZrO_2_ grains and thus, larger interfacial areas for ZrO_2_−2Li_2_ZrCl_6_, this result implies increased Li^+^ conduction at the ZrO_2_−2Li_2_ZrCl_6_ interfaces^42^. Although the Li^+^ conductivities for nanocomposites of Li_2_ZrCl_6_ with different metal oxide nanoparticles were consistently higher than that of Li_2_ZrCl_6_, none of them were as high as the conductivity of the mechanochemically prepared ZrO_2_−2Li_2_ZrCl_6_ HNSE (Supplementary Fig. 11). To the best of our knowledge, the Li^+^ HNSE was the first inorganic superionic conductor that exploited the interfacial effect to promote conduction with ionic conductivity reaching 1 mS cm^−1^ at 25-30 °C. Furthermore, the maximum ionic conductivity of the HNSE Na^+^ analogues was approximately an order of magnitude greater (0.11 mS cm^−1^ for 0.13ZrO_2_−0.61NaCl-0.26Na_2_ZrCl_6_) than that of Na_2_ZrCl_6_ (0.011 mS cm^−1^) (Fig. 2b, c, and Supplementary Fig. 12). This value is the highest among the Na^+^ halide SEs developed thus far^32–34^.

The extended compositions of Li^+^ and Na^+^ HNSEs were further explored, as shown in the contour map of ionic conductivity for the ternary system of ZrO_2_-ACl-A_2_ZrCl_6_ (A = Li or Na) with the volume fraction scale (Fig. 2d). The corresponding Li^+^ and Na^+^ conductivities are summarized in Supplementary Tables 5 and 6. Four features are worth noting. First, the ionic conductivities of the HNSEs were enhanced as the ZrO_2_ fraction was increased. Second, compared to Li_2_ZrCl_6_ (or Na_2_ZrCl_6_), 0.53LiCl-Li_2_ZrCl_6_ (or 0.53NaCl-Na_2_ZrCl_6_) showed higher ionic conductivities of 0.70 vs. 0.40 mS cm^−1^ for Li^+^, implying enhanced ionic conduction at the ACl-A_2_ZrCl_6_ interfaces. However, from the result where the LiCl/ZrO_2_ ratio varied with the fixed Li_2_ZrCl_6_ fraction, ZrO_2_ was more effective than LiCl in enhancing the ionic conductivity of the HNSEs. Third, the maximum ionic conductivities of the HNSEs were found for the ternary HNSEs of 0.44ZrO_2_−1.26LiCl-0.56Li_2_ZrCl_6_ (1.3 mS cm^−1^) and 0.13ZrO_2_−0.61NaCl-0.26Na_2_ZrCl_6_ (0.11 mS cm^−1^). Finally, an accessible compositional area was restricted to Region I (Fig. 2d). Alternative oxygen sources can be used to access the region beyond the upper boundary limit of Region I, leading to a further enhancement of the ionic conductivities.

### Interfacial superionic conduction of HNSEs

In many previous reports about AX/metal oxide systems (A = Li, Cu, Ag; X = Cl, Br, I) with behaviour similar to that of the HNSEs, anomalously high ionic conductivities were attributed to the space charge layer (SCL) effect, which has been an issue of debate^42,47^. The ionic conduction between Li^+^ conductors and metal oxides (non-Li^+^-conducting materials) can be improved by more charge carriers at the interfaces through SCL^42^. However, the superionic conduction of HNSE could not be explained solely by the conventional SCL effect observed in AX/metal oxide systems due to their much lower ionic conductivities (10^−1^ to 10^−3^ mS cm^−1^ at 25 °C). To elucidate the underlying mechanism for the enhanced ZrO_2_/Li_2_ZrCl_6_ interfacial superionic conduction of HNSEs, we conducted DFT calculations.

**Fig. 3:**
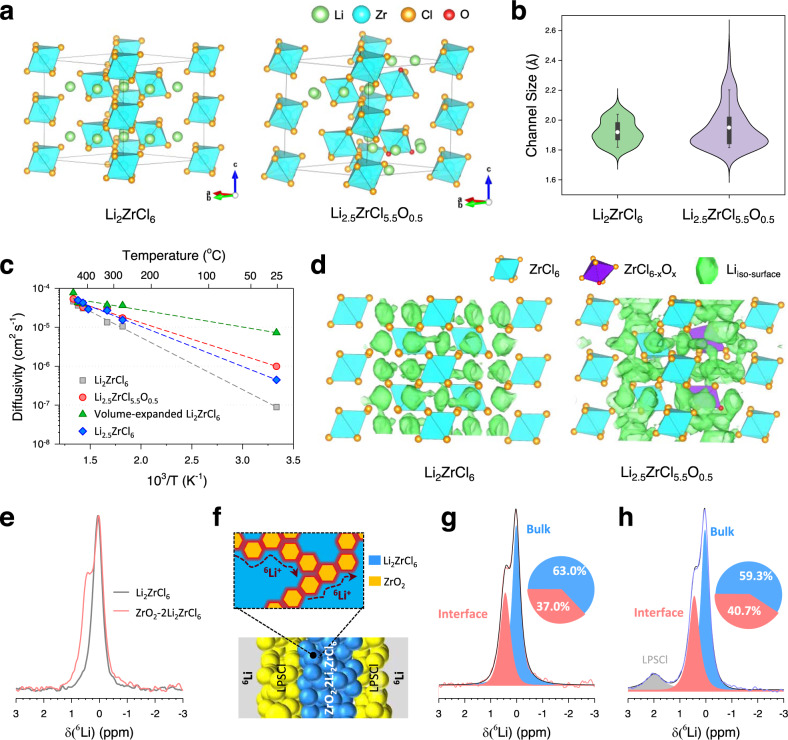
Interfacial Li^+^ superionic conduction in HNSEs. **a** Crystal structures of Li_2_ZrCl_6_ and Li_2.5_ZrCl_5.5_O_0.5_. **b** Topological analysis and channel size of Li_2_ZrCl_6_ and Li_2.5_ZrCl_5.5_O_0.5_. The white circle, box and whisker indicate the mean value, values from the 25% to the 75% percentiles and values from the 10% to the 90% percentiles, respectively. **c** Arrhenius plots of the AIMD simulation extrapolated to 300 K. **d** Li probability density at 600 K in ~200 ps (isosurface value P = P_max_/50). **e** ^6^Li MAS-NMR spectra for Li_2_ZrCl_6_ and ZrO_2_-2Li_2_ZrCl_6_. **f** Schematic of ^6^Li|LPSCl|(ZrO_2_-2Li_2_ZrCl_6_)|LPSCl|^6^Li symmetric cells with interfacial superionic Li^+^ diffusion pathways. (Blue: Li_2_ZrCl_6_, yellow: ZrO_2_, red: interface or interphase) **g**, **h** ^6^Li MAS-NMR spectra of ZrO_2_-2Li_2_ZrCl_6_ before (**g**) and after cycling (**h**).

We considered a small amount (*x* = 0.5 in Li_2+*x*_ZrCl_6-*x*_O_*x*_) of anion exchange between Li_2_ZrCl_6_ (LZC) and ZrO_2_ at the interface during synthesis and an excess Li concentration at the LZC side for local charge compensation with oxygen substitution (see Supplementary Fig. 13 and Supplementary Note 4). Figure 3a and Supplementary Fig. 14 show our model structures of LZC, Li_2.5_ZrCl_5.5_O_0.5_ (LZCO), ZrO_2_, and ZrO_2-x_Cl_x_. After structural relaxation using DFT calculations, the structure of LZCO showed elongation of the average bond length of Zr-Cl compared with that of LZC, and Cl-substitution of ZrO_2_ drove an increase in the Zr-Zr distance (Supplementary Table 7 and Supplementary Fig. 15). These structural changes with anion exchange between LZC and ZrO_2_ agreed with the experimental observation in Zr K-edge EXAFS results (Fig. 1c, Supplementary Table 2), which demonstrated a slight increase in Zr-Cl bond length (from 2.46 to 2.47 Å) and broadening of the Zr-Zr peak (~3.5 Å) for the HNSE. The distances among ZrCl_6-*x*_O_*x*_ octahedra in LZCO increased due to larger electrostatic repulsion of Cl^−^─O^2−^ than that of Cl^−^─Cl^−^, resulting in lattice volume expansion (Supplementary Fig. 16). A Zr-O bond that was shorter than the Zr-Cl bond drove the shrinkage of ZrCl_6-*x*_O_*x*_ octahedra (Supplementary Table 7). These two factors together enlarged the Li^+^ transport channel (Fig. 3b), which boosted the migration of Li ions. In addition, the Li^+^ concentration was higher in LZCO than in LZC, which enriched the ionic carrier concentration. Notably, LZCO was energetically less favourable than the composite of ZrCl_4_, LiCl, and ZrO_2_ (Supplementary Table 8), indicating that it would be challenging to synthesize LZCO directly in a general reaction. However, its energy above the hull is not that high; thus, it can be partially formed at the interface by high-energy mechanochemical synthesis.

To further verify the composition of ZrO_2_−2Li_2_ZrCl_6_ and validate the O-substituted LZCO populated at the interfaces, we performed PDF fitting across different refinement ranges (low r range of 1.5−10 Å; high r range of 10−30 Å) as shown in Supplementary Fig. 17 and Supplementary Table 9. After extensive preliminary fitting attempts using various combinations of model structures, including the LZCO interphase structure provided via DFT calculations (Supplementary Table 7), the best-fit result (*R*_*w*_ = 10.9%) is achieved by including the LZCO interphase with minor Li_2_O impurity (Supplementary Table 10) in the low r range (1.5 ~ 10 Å), where the interface regime becomes prevailing. On the other hand, the medium and average structure in the high r range (10 ~ 30 Å) can be well represented by a single Li_2_ZrCl_6_ structure. The calculated composition obtained by the PDF fit corresponds to 1.47Li_2_ZrCl_2_−0.36Li_2.5_ZrCl_5.5_O_0.5_(interphase)−1.01ZrO_2_−0.16Li_2_O (Supplementary Table 11). This result strongly supports the presence of a LZCO interphase between ZrO_2_ and LZC nanodomains.

Ab initio molecular dynamics (AIMD) simulations were also performed to verify superionic conduction at the HNSE interface (Fig. 3c, Supplementary Fig. 18 and Table 12). LZCO shows a faster Li^+^ diffusion than LZC at all temperatures for which AIMD simulations were performed, but also has a gentle slope compared with the Li^+^ diffusivities of LZC. It is expected that Li^+^ diffusion shows ~11 times faster for LZCO than LZC at 300 K. To explain the origin of such fast diffusion, we generated two hypothetical structures of LZC: (1) off-stoichiometric Li-rich LZC (Li_2.5_ZrCl_6_), where the amount of Li in the structure was simply increased, and (2) volume-expanded LZC, where the lattice parameters were the same as those of LZCO. The diffusivities of both hypothetical structures at 300 K were higher than that of the LZC structure (Fig. 3c), implying that both volume expansion and Li enrichment positively impacted facile diffusion. The Li probability density showed a more 3D-connected and broadened Li isosurface in LZCO than the LZC case, revealing that the overall expanded Li^+^ diffusion pathway activated the movement of Li^+^ (Fig. 3d and Supplementary Fig. 19). In addition, we discuss more detailed observations of the Li^+^ diffusion behaviour near the neighbouring anions (O^2−^, Cl^−^) of LZCO in Supplementary Fig. 20 and the Supplementary Note 5. To effectively exploit interfacial superionic conduction, it is crucial to not only accumulate Li^+^ carriers at the interface but also widen Li^+^ channels via anion substitution. Notably, the interface formed by a poor ionic conductor (LiCl) and superionic conductor (Li_2_ZrCl_6_) only promoted Li accumulation by the chemical potential difference, excluding the effect of the channel size increase. This may result in an insignificant increase in ionic conduction compared to that in the HNSE of ZrO_2_-LZC, as shown in our experimental measurements (Fig. 2d). More ZrO_2_-LZC interfaces in HNSEs result in more interfacial phases (LZCO), further boosting the superionic conduction.

To probe the local Li^+^ environments at the interfaces of the HNSEs, ^6^Li MAS-NMR measurements were conducted for Li_2_ZrCl_6_ and ZrO_2_−2Li_2_ZrCl_6_. For both spectra, the main signals are shown at 0.05 ppm corresponding to Li_2_ZrCl_6_ (Fig. 3e). However, in sharp contrast to Li_2_ZrCl_6_, the ZrO_2_−2Li_2_ZrCl_6_ HNSE spectrum exhibited a distinct peak at ~0.42 ppm. In a previous study on LiBH_4_/Al_2_O_3_, a similar shoulder peak was observed and attributed to the highly conductive interface region affected by Al_2_O_3_^48^. Because the electronegativity of oxygen is larger than that of chlorine, electrons in Li^+^ at the interfaces will be withdrawn more for HNSE than for Li_2_ZrCl_6_, which implies more deshielding and thus explains the evolution of the peak at the higher chemical shift^48,52^. In this regard, the shoulder peak at ~0.42 ppm for the HNSE likely corresponded to the O-substituted interphase suggested by the DFT calculations. Furthermore, the Li^+^ migration pathways in the nanocomposite structure of ZrO_2_−2Li_2_ZrCl_6_ were investigated by ^6^Li exchange experiments using ^6^Li|LPSCl|HNSE|LPSCl|^6^Li symmetric cell (Fig. 3f)^53^. After repeated cycling (Supplementary Fig. 21), the ^6^Li NMR spectrum of the HNSE was compared with the result for pristine HNSE (Fig. 3g, h). The area fraction of the interphase peak increased from 37.0 to 40.7% after cycling, which corroborated the promoted interfacial Li^+^ conduction.

### General applicability of the HNSE strategy in interfacial conduction

The material space for the HNSEs is expandable beyond the ZrO_2_-ACl-A_2_ZrCl_6_ system, as illustrated schematically in Supplementary Fig. 22. Two-step mechanochemical protocols can be used to produce multimetal HNSEs. In the first step, Li_2_O and metal chloride (MCl_y_) react to form MO_x_-LiCl nanocomposites. The subsequent step involves the reaction with additional metal halides (M’X_y_ (with M”X_y_)) to form multimetal HNSEs, such as MO_x_-LiCl-Li_a_M’Cl_b_ and MO_*x*_-LiCl-Li_a_M’M”Cl_b_. Following this two-step path, Al_2_O_3_−3Li_2_ZrCl_6_ and SnO_2_−2Li_2_ZrCl_6_ HNSEs were prepared (Supplementary Fig. 23). Similar to ZrO_2_−2Li_2_ZrCl_6_, the Al_2_O_3_−3Li_2_ZrCl_6_ and SnO_2_−2Li_2_ZrCl_6_ HNSEs exhibited enhanced Li^+^ conductivities of 0.88 and 1.6 mS cm^−1^, respectively, compared to that of Li_2_ZrCl_6_ (Supplementary Fig. 23). Notably, the single-step protocol using a mixture of Li_2_O, AlCl_3_, and ZrCl_4_ resulted in a poor Li^+^ conductivity of 0.3 mS cm^−1^ due to the formation of an unfavourable LiCl component. In our previous study, the Li^+^ conductivity of Li_2_ZrCl_6_ was improved via aliovalent substitution with Fe^3+^, showing a maximum conductivity of 1.0 mS cm^−1^ (Li_2.25_Zr_0.75_Fe_0.25_Cl_6_)^26^. Such HNSEs can also be prepared by the two-step protocol. To avoid the reaction of FeCl_3_ with Li_2_O, after ZrO_2_−2Li_2_ZrCl_6_ was prepared, FeCl_3_ and LiCl were added to substitute Fe^3+^ in Li_2_ZrCl_6_. Li_2+*x*_Zr_1-*x*_Fe_*x*_Cl_6_ showed enhanced Li^+^ conductivities for HNSEs over the entire range of *x*, reading a maximum Li^+^ conductivity of 1.4 mS cm^−1^ (Supplementary Fig. 24).

**Fig. 4:**
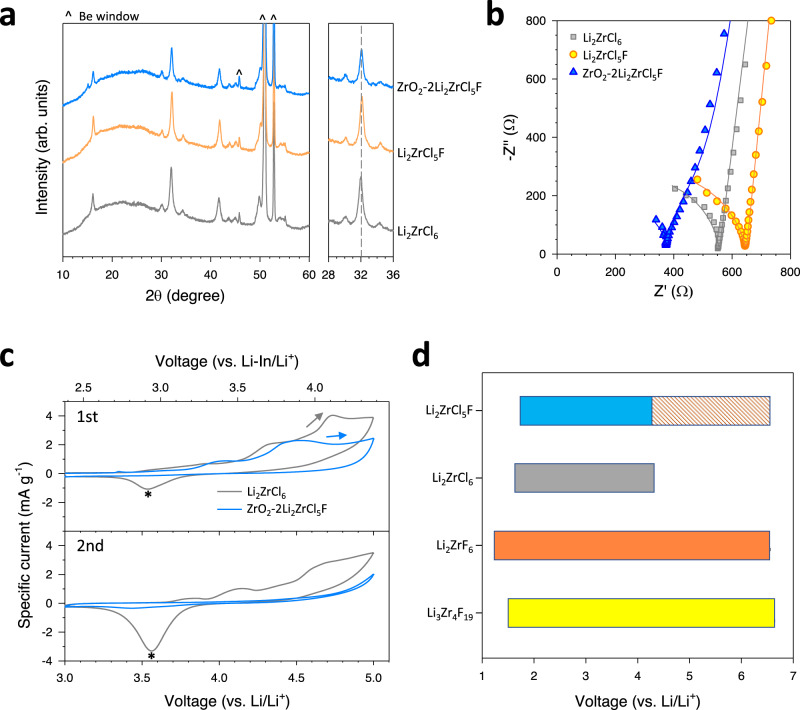
HNSE for F-substituted Li_2_ZrCl_6_ (ZrO_2_-2Li_2_ZrCl_5_F). **a**–**c** XRD patterns (**a**) and Nyquist plots of Ti|SE|Ti symmetric cells (**b**) for Li_2_ZrCl_6_, Li_2_ZrCl_5_F, and ZrO_2_-2Li_2_ZrCl_5_F. The fitted lines are also plotted in (**b**). The symbols represent the raw data, and the lines correspond to the fitted results obtained from the equivalent circuit model, as described in Supplementary Fig. 10. **c** CV curves for (SE-C)|SE|LPSCl|(Li-In) cells between 3.0 and 5.0 V (vs. Li/Li^+^) at 0.1 mV s^−1^ and 30 °C. **d** Calculated electrochemical stability of Li_2_ZrCl_6_, Li_2_ZrCl_5_F, Li_2_ZrF_6_ and Li_3_Zr_4_F_19_. Li_2_ZrF_6_ and Li_3_Zr_4_F_19_ are thermodynamically stable passivating interphases that can be decomposed from Li_2_ZrCl_5_F. The dashed box of Li_2_ZrCl_5_F represents the electrochemical stability window stabilized by the decomposition products of Li_2_ZrCl_5_F (Li_2_ZrF_6_ and Li_3_Zr_4_F_19_). Calculation details are found in the “Methods” section.

Importantly, owing to the small ionic size and low polarizability of F^-^ (r(F^−^) = 119 pm, r(Cl^−^) = 181 pm, ionic radius values represent the crystal ionic radii^54^), F-substitution in SEs generally decreases the ionic conductivity^37,38^, but it can be counterbalanced by applying the HNSE synthetic strategy. From the XRD results, the F-substitution limit in Li_2_ZrCl_6-*x*_F_*x*_ is approximately *x* = ~1.0 (Supplementary Fig. 25). The XRD pattern of the ZrO_2_−2Li_2_ZrCl_5_F HNSE showed a slight positive shift of the (301) peak at ~32^o^ (Fig. 4a), indicating the successful fluorination of the Li_2_ZrCl_6_ domain. Consistent with the result for the ZrO_2_−2Li_2_ZrCl_6_ HNSE, the ZrO_2_−2Li_2_ZrCl_5_F HNSE also exhibited a shoulder peak in ^6^Li MAS-NMR spectrum (Supplementary Fig. 26). This result suggests an O-substituted Li_2_ZrCl_5_F at the interface as revealed for ZrO_2_−2Li_2_ZrCl_6_. The HRTEM results are also provided in Supplementary Fig. 27. While the Li^+^ conductivity of Li_2_ZrCl_6_ was decreased to 0.35 mS cm^−1^ by fluorination (Li_2_ZrCl_5_F), the ZrO_2_−2Li_2_ZrCl_5_F HNSE exhibited even higher Li^+^ conductivity of 0.49 mS cm^−1^ compared to Li_2_ZrCl_6_ (0.40 mS cm^−1^, Fig. 4b). Notably, when assessed by cyclic voltammetry (CV) tests at 30 °C, ZrO_2_−2Li_2_ZrCl_5_F exhibited a smaller integrated current of 1.98 mA V g^−1^ up to 5.0 V (vs. Li/Li^+^, although all cells in this work utilized a Li-In alloy electrode instead of metallic Li, we report the voltage vs. Li/Li^+^ instead of vs. Li-In/Li^+^ as a convention in the electrochemistry field. Hereafter, it is to be understood that all reported cell voltages vs. Li/Li^+^ as being shifted by 0.62 V from the cell voltage vs. Li-In/Li^+^ for better comparison with literature data. Further detailed discussion is provided in Supplementary Note 6, Supplementary Fig. 28), compared to Li_2_ZrCl_6_ (2.76 mA V g^−1^, Fig. 4c, Supplementary Table 13). The difference became even larger at the second cycle (0.55 vs. 2.00 mA V g^−1^ for ZrO_2_−2Li_2_ZrCl_5_F and Li_2_ZrCl_6_, respectively). Furthermore, Li_2_ZrCl_6_ showed a cathodic peak at ≈3.5 V (vs. Li/Li^+^) at the first cycle and they intensified further at the second cycle, which is indicated by an asterisk in Fig. 4c. It is speculated that the cathodic currents originate from the decomposition byproducts formed during the prior positive scan^55^. By contrast, ZrO_2_−2Li_2_ZrCl_5_F exhibited negligible cathodic currents, which agrees with the substantially lower oxidation currents compared with Li_2_ZrCl_6_, thus suggesting the passivating behaviour of ZrO_2_−2Li_2_ZrCl_5_F. The DFT results consistently revealed that the oxidative limit of Li_2_ZrCl_5_F (4.274 V vs. Li/Li^+^) was slightly lower than that of Li_2_ZrCl_6_ (4.307 V vs. Li/Li^+^), but the formation of desirable F-based passivating interphase materials such as Li_2_ZrF_6_ and Li_3_Zr_4_F_19_ can increase the range of the anodic limit (Fig. 4d and Supplementary Table 14)^37,38^.

### Electrochemical energy storage performances of all-solid-state Li-based cells with HNSEs

**Fig. 5:**
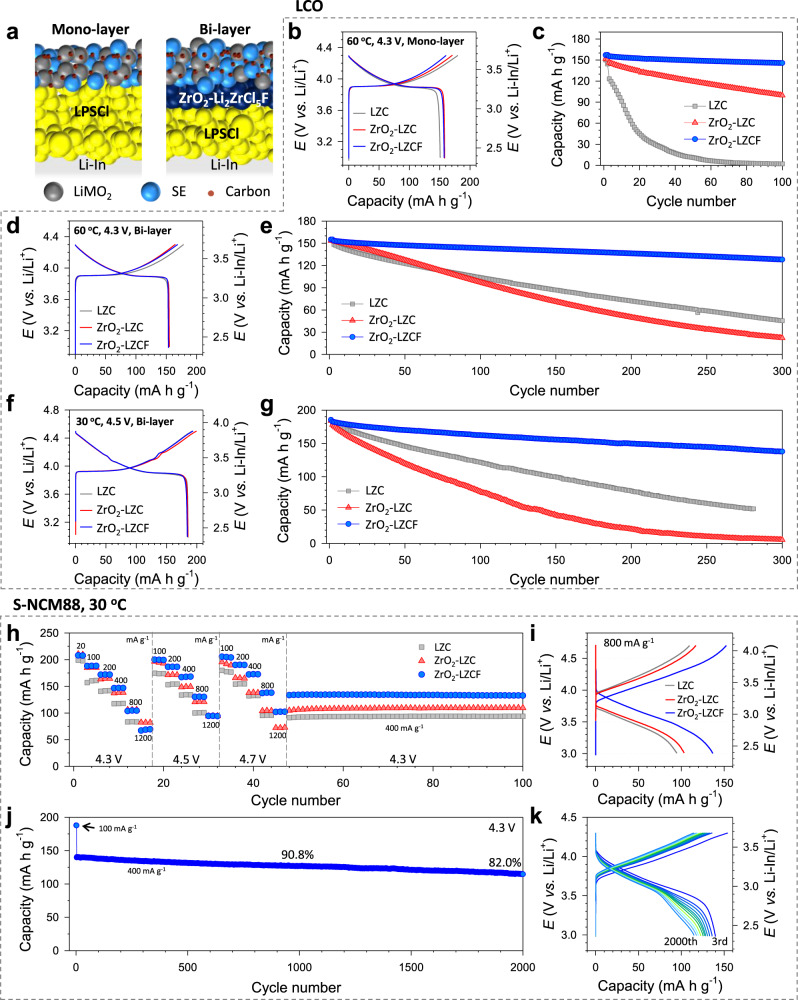
LCO and S-NCM88 ASSB cells employing HNSEs (ZrO_2_-2Li_2_ZrCl_6_ and ZrO_2_-2Li_2_ZrCl_5_F vs. Li_2_ZrCl_6_). **a** Schematics of ASSB cells employing an LPSCl monolayer or (ZrO_2_-LZCF)|LPSCl bilayer. **b**, **c** First-cycle charge–discharge voltage profiles at 16.4 mA g^−1^ and 60 °C (**b**) and cycling performances at 82.0 mA g^−1^ (**c**) for LCO electrodes with an LPSCl monolayer cycled up to 4.3 V (vs. Li/Li^+^). **d**–**g** First-cycle charge–discharge voltage profiles at 16.4 mA g^−1^ and cycling performances at 82.0 mA g^−1^ for LCO electrodes with a ZrO_2_-LZCF bilayer cycled up to 4.3 V at 60 °C (**d**, **e**) and 4.5 V (vs. Li/Li^+^) at 30 °C (**f**, **g**). **h**, **i** Rate capabilities with varying cut-off voltages (**h**) and the corresponding charge–discharge voltage profiles at 800 mA g^−1^ (**i**) for S-NCM88 electrodes. **j**, **k** Cycling performance for the S-NCM88 electrode using ZrO_2_-LZCF at 400 mA g^−1^ (**j**) and the corresponding charge–discharge voltage profiles (**k**). LPSCl monolayers were used for the S-NCM88 cells. The capacity retention was determined by comparing capacities at the 1000th and 2000th cycles to that at the 3rd cycle. For the LCO-based electrodes, the specific capacity of 164 mA g^−1^ corresponds to 1.30 mA cm^−2^ and, for the S-NCM88-based electrodes the specific capacity of 200 mA g^−1^ corresponds to 1.09 mA cm^−2^. The specific current and capacity were determined based on the mass of active material (10.2 mg for LCO and 7.3 mg for S-NCM88). All the cells were cycled under a pressure of 70 MPa.

The mechanochemically prepared HNSEs ZrO_2_-2Li_2_ZrCl_6_ and ZrO_2_-2Li_2_ZrCl_5_F (hereafter referred to as ZrO_2_-LZC and ZrO_2_-LZCF, respectively) were tested in combination with uncoated LCO and S-NCM88 positive electrodes in ASSB cells, and the results were compared to those obtained using Li_2_ZrCl_6_ (hereafter referred to as LZC) as shown in Fig. 5 and Supplementary Fig. 29. LPSCl monolayers or (ZrO_2_-LZCF)|LPSCl bilayers and Li-In counter electrodes were employed for the ASSB cells (Fig. 5a). At 30 °C and a cut-off voltage of 4.3 V (vs. Li/Li^+^), all three LCO cells exhibited good performances with marginal differences in terms of their capacity, initial Coulombic efficiency (ICE), and capacity retention at 82.0 mA g^−1^ (Supplementary Fig. 30, Supplementary Table 15). However, upon increasing the temperature to 60 °C, the performance difference became distinct in the descending order of ZrO_2_-LZCF > ZrO_2_-LZC » LZC (Fig. 5b, c, Supplementary Fig. 29a); the ICEs were 94.5%, 91.8%, and 80.3%, and the values of the capacity retention at the 100th cycle were 93.7%, 68.0%, and 1.7%, respectively. When other major halide SEs like Li_3_YCl_6_ and Li_3_InCl_6_ were tested at 60 °C, capacity fading was also observed (Supplementary Fig. 31). The poor cycling performances of LCO electrodes using halide SEs were uncommon^7,20,25,26,31,38^. Despite being overlooked, these results could be associated with compatibility with the sulfide SE used for the SE layer^41^. The incompatibility between sulfide and halide SEs impacted Coulombic efficiency. For monolayer cells utilizing LZC or ZrO_2_-LZC at 30 °C and 60 °C (Supplementary Figs. 29 and 30), Coulombic efficiency values continuously increased beyond 100%, suggesting side reactions between the sulfide and halide SEs. The good capacity retention and consistent Coulombic efficiency with ZrO_2_-LZCF indicated the compatibility of ZrO_2_-LZCF with LPSCl. The cells using the LZC catholyte with the (ZrO_2_-LZCF)|LPSCl bilayer, where direct contact between LZC and LPSCl was prevented (Fig. 5d, e, Supplementary Fig. 29b), consistently showed degrading but improved overall performance, from 1.7% to 70.0% for the capacity retention at the 100th cycle. In contrast, for the cells with the LCO electrodes employing fluorinated HNSE ZrO_2_-LZCF, the cycling performance was satisfactory, regardless of the separating SE layer, with capacity retention of 93.7% and 93.4% after 100 cycles using mono- and bilayers, respectively. The corresponding electrochemical impedance spectroscopy (EIS) results are provided in Supplementary Fig. 32 and the equivalent circuit and the fitted results with detail discussion are also provided in Supplementary Fig. 10b, Supplementary Table 16 and Supplementary Note 7. The still degrading performance of the cells using LZC with the (ZrO_2_-LZCF)|LPSCl bilayer originates from its electrochemical instability and/or incompatibility with cathode material (LCO). Based on the results obtained thus far, we conclude that the reason for the poor performance of the LZC|(LPSCl monolayer) combination at 60 °C was the incompatibility of LZC with LPSCl and LiMO_2_, and electrochemical instability, whereas ZrO_2_-LZCF is compatible and electrochemically stable.

DFT calculations were conducted to assess the compatibilities of LCO and sulfide SEs with halide SEs. While the mixtures of LCO and halide SEs (Li_2_ZrCl_6_, Li_3_YCl_6_, Li_3_InCl_6_) showed low mutual reaction energies (|ΔE_D, mutual_| < 0.4 eV/formula unit (f.u.)), the halide/LPSCl SE mixtures exhibited high reaction energies over 0.4 eV/f.u., indicating their poor compatibility (Supplementary Table 17). For LZC and ZrO_2_-LZCF, an EIS experiment using Ti|(halide-LPSCl mixture)|Ti cells stored at 60 °C showed marginal differences in Nyquist plots after a week (Supplementary Fig. 33), indicating a stable halide-LPSCl interface under no electrochemical driving forces (Supplementary Note 8). A control EIS experiment was also performed using Li-In|LPSCl|halide-carbon cells charged to 4.3 V (vs. Li/Li^+^) at 60 °C so that the halide-LPSCl interfaces are subjected to the high voltage (Supplementary Fig. 34). From qualitative analysis of the Nyquist plots, it can be seen that the impedance continuously increased for the cells using LZC. In contrast, the impedance increased for an hour and then saturated for the cell comprised of ZrO_2_-LZCF, which indicated good passivating behaviour. In summary, the halide/sulfide incompatibility, which has often been overlooked, was identified clearly, and it was found to be driven electrochemically at elevated temperatures. Importantly, F-substituted chloride SEs are free from this halide/sulfide incompatibility issue and thus allow for the use of sulfide monolayers, which simplifies the fabrication of ASSB cells for practical applications, as illustrated in Supplementary Fig. 35. High-voltage stabilities of up to 4.5 V (vs. Li/Li^+^) for LCO electrodes using HNSEs were also tested in ASSB cells with bilayer at 30 °C (Fig. 5f, g and Supplementary Fig. 29c). The capacity retention with ZrO_2_-LZCF was much higher (82.2% after 200 cycles) than that with LZC or ZrO_2_-LZC (43.8% and 12.1%, respectively), which agreed well with the results of CV (Fig. 4c) and DFT calculations. The charge–discharge voltage profiles for the LCO electrodes at different cycles are shown in Supplementary Fig. 36.

The high-voltage stability of ZrO_2_-LZCF enables pushing the upper cut-off voltage limit, offering an extra margin that counteracts polarization-driven capacity loss^56^. This feature could be advantageous for fast charging (Supplementary Fig. 37)^38^. Moreover, the elevated ion conduction in the HNSEs further boosts the fast-charging capability. The rate capabilities of cracking-free S-NCM88 positive electrodes in combination with Li-In negative electrodes were thus assessed using ASSB cells with LPSCl monolayers and stepwise increasing cut-off voltages of 4.3, 4.5, and 4.7 V (vs. Li/Li^+^) at various specific currents and 30 °C (Fig. 5h, i, and Supplementary Fig. 38). The S-NCM electrodes showed high initial discharge capacities of 199, 210, and 208 mA h g^−1^ at 20.0 mA g^−1^ for LZC, ZrO_2_-LZC, and ZrO_2_-LZCF, respectively. At the lowest cut-off voltage of 4.3 V, the rate capabilities decreased in the following order: ZrO_2_-LZC ≈ ZrO_2_-LZCF > LZC, which was consistent with the Li^+^ conductivity order (ZrO_2_-LZC: 1.1 mS cm^−1^ > ZrO_2_-LZCF: 0.49 mS cm^−1^ > LZC: 0.40 mS cm^−1^) and the stability of LZCF. At higher cut-off voltages, especially 4.7 V, the electrodes employing ZrO_2_-LZCF outperformed the others, emphasizing the effect of high-voltage stability. Finally, the single-NCM88 electrodes employing ZrO_2_-LZCF cycled at 400 mA g^−1^ and 30 °C showed a long cycle life of 90.8% capacity retention after 1000 cycles (Fig. 5j, k Supplementary Fig. 29d), which is well positioned in the state-of-the-art literature of ASSBs^7,20,25,26,31,38^.

In summary, we reported a nanocomposite strategy for halide SEs to enhance their ionic conductivity and the compatibility with sulfide SEs. The mechanochemical reaction using Li_2_O as an oxygen source created ZrO_2_(-ACl)-A_2_ZrCl_6_ (A = Li or Na) with a network nanostructure wherein large-area interfaces were percolated. Despite the presence of the ionically insulating ZrO_2_ phase in the HNSEs, interfacial superionic conduction enhanced ionic conductivities for Li^+^ and Na^+^ halide SEs: from 0.40 to 1.3 mS cm^−1^ for ZrO_2_-2LiCl-Li_2_ZrCl_6_ and from 0.011 to 0.11 mS cm^−1^ for 0.13ZrO_2_-0.61NaCl-0.26Na_2_ZrCl_6_. The applicability of the HNSE approach to other metals was highlighted, e.g., Al_2_O_3_-3Li_2_ZrCl_6_ (0.88 mS cm^−1^), SnO_2_-2Li_2_ZrCl_6_ (1.6 mS cm^−1^) and 0.75ZrO_2_-Li_2.25_Zr_0.75_Fe_0.25_Cl_6_ (1.4 mS cm^−1^). DFT calculations combined with experimental synchrotron X-ray measurements and analysis revealed that the conduction behaviour at the ZrO_2_/Li_2_ZrCl_6_ interface originated from local anion substitution, thereby leading to widened ion transport channels and increased local Li content at the interface. Partially oxidized Li_2_ZrCl_6_ at the populated interfaces was responsible for anomalous superionic conduction in the HNSEs, and active interfacial conduction was probed by ^6^Li MAS-NMR measurements and analysis. Our research work provides design principles for HNSEs, and they can be applied to other combinations of non-Li^+^-conducting compounds and halide superionic conductors. In addition, the HNSE strategy counteracted the degradation of Li^+^ conductivity by F-substitution. Finally, the HNSEs, especially the F-substituted HNSE ZrO_2_-2Li_2_ZrCl_5_F, demonstrated good electrochemical energy storage performances in ASSB cells using LiCoO_2_ or S-NCM88 positive electrodes and Li-In negative electrodes in terms of high-voltage stability up to 4.7 V (vs. Li/Li^+^), compatibility with LPSCl and LiMO_2_ cathode materials at 60 °C, rate capability, and long-term cycle life (82.0% capacity retention through 2000 cycles with respect to that at the 3rd cycle at 400 mA g^−1^ and 30 °C). Notably, in this study, the HNSEs demonstrated the use of cost-effective elements, such as Zr and Al. Our approach provides not only an advancement in practical all-solid-state technology but also a dimension that widens the materials chemistry spaces for superionic conduction.

## Methods

### Preparation of materials

To prepare ZrO_2_(-ACl)-A_2_ZrCl_6_ (A = Li or Na), a stoichiometric mixture of Li_2_O (99.5%, Alfa Aesar) or Na_2_O (80%, Sigma Aldrich, ~20% Na_2_O_2_), LiCl (99.99%, Sigma Aldrich) or NaCl (99.99%, Alfa Aesar), and ZrCl_4_ (99.99%, Sigma Aldrich) was ball-milled at 600 rpm for 20 h in a ZrO_2_ vial with ZrO_2_ balls using Pulverisette 7PL (Fritsch GmbH) under Ar atmosphere. To prepare fluorinated HNSE ZrO_2_-2Li_2_ZrCl_5_F, a stoichiometric mixture (Li_2_O: ZrF_4_: ZrCl_4_ = 2: 0.5: 2.5) of Li_2_O (99.5%, Alfa Aesar), ZrF_4_ (99.9%, Sigma Aldrich) and ZrCl_4_ (99.99%, Sigma Aldrich) was ball-milled under the same condition as for the conventional HNSEs. To prepare *n*M_y_O_z_-Li_2_ZrCl_6_ nanomixtures, M_y_O_z_ nanoparticles were ball-milled at 600 rpm for 20 h in a ZrO_2_ vial with ZrO_2_ balls using Pulverisette 7PL (Fritsch GmbH). ZrO_2_ (99.95%, 20 nm) and Al_2_O_3_ (≥95%, 50 nm) nanopowders were purchased from Avention and Sigma Aldrich, respectively. Fumed SiO_2_ powders were obtained from Sigma Aldrich. For the preparation of Li_6_PS_5_Cl, a stoichiometric mixture of Li_2_S (99.9%, Alfa Aesar), P_2_S_5_ (99%, Sigma Aldrich), and LiCl (99.99%, Sigma Aldrich) was ball-milled at 600 rpm for 10 h in a ZrO_2_ vial with ZrO_2_ balls, followed by annealing at 550 °C for 6 h under an Ar atmosphere.

### Material characterization

Powder XRD patterns were collected using a Rigaku MiniFlex600 diffractometer with Cu K_α_ radiation (*λ* = 1.5406 Å). XRD cells containing hermetically sealed SE samples with a Be window were mounted on an XRD diffractometer and measured at 40 kV and 15 mA. X-ray total-scattering data were collected at beamline 28-ID-1 PDF at the National Synchrotron Light Source II (NSLSII) of Brookhaven National Laboratory with an X-ray energy of 74.5 keV (*λ* = 0.1665 Å). The prepared samples were loaded into polyimide (Kapton) tubes and hermetically sealed with epoxy resin. The 2D images were reduced to a 1D pattern with Ni calibrant using Dioptas software^57^, and the PDF G(r) was obtained from Fourier transformation with a Q range of 1.5−23 Å^−1^ from xPDFsuite^58^. The PDF G(r) of the HNSEs was refined using various structural models, including Li_2_ZrCl_6_ and ZrO_2_ with adjustment of the scale factor, lattice parameter, and atomic displacement parameters. Zr K-edge XAS measurements were conducted at the 7D and 10 C beamlines of the Pohang Accelerator Laboratory (PAL) using a Si (111) double-crystal monochromator in transmission and fluorescence modes. Energy calibration was carried out using the reference spectra of the Zr metal foils. The Cl K-edge XANES spectra were measured in fluorescence yield mode at the beamline 8-BM TES of NSLSII and 16A1 of Taiwan Light Source. XANES and EXAFS data were processed using the Demeter software package^59^. The extracted EXAFS signal, *k*^3^*χ*(*k*), is Fourier transformed in the *k*-range of 3.2−11.2 Å^−1^ and fitted in the R-range of 1.3−3.0 Å (Li_2_ZrCl_6_) and 1.3−3.2 Å (ZrO_2_-2Li_2_ZrCl_6_ and nZrO_2_-2Li_2_ZrCl_6_). ^6^Li MAS-NMR spectra were obtained at 170 K on a 400 MHz Advance II + system (Bruker solid-state NMR) at the KBSI Seoul Western Center, for which the ^6^Li resonance frequency was 58.862 MHz and a rotation frequency of 10 kHz was applied. The external chemical shift reference of the LiCl powder spun at 4 kHz was calibrated to 0 ppm. The NMR sample powders were sealed in a 4 mm ZrO_2_ rotor in an Ar-filled glove box. ^6^Li^+^-ion nonblocking symmetric cells of ^6^Li|LPSCl|(ZrO_2_-2Li_2_ZrCl_6_)|LPSCl|^6^Li were assembled as follows. ^6^Li foils were prepared by compressing ^6^Li chunks (95%, Sigma Aldrich). ^6^Li|LPSCl|(ZrO_2_-2Li_2_ZrCl_6_)|LPSCl|^6^Li cells were cycled at each cycle for 1 h at 500 µA cm^−2^ and 60 °C. For the HRTEM measurements, the samples were loaded onto a lacey Cu grid, and HRTEM images were obtained using a JEM-ARM 200 F NEOARM (JEOL). For the TEM measurements in Supplementary Fig. 3g-i, the samples were loaded onto a lacey Cu grid and mounted on a double-tilt cryo-TEM holder with vacuum transfer (Double tilt LN2 Atmos Defend Holder, Mel-Build) to prevent air exposure of the samples. TEM images were obtained using a JEM-2100F (JEOL).

### Theoretical calculations

First-principles calculations were carried out using the Vienna Ab initio Simulation Package (VASP)^60^. Generalized gradient approximation (GGA) exchange-correlation with the Perdew–Burke–Ernzerhof (PBE) functional^61^ was adopted, alongside the projector-augmented wave (PAW) method. A plane-wave cut-off energy of 520 eV was used, and the cell shape, cell volume, and atomic positions of each structure were fully relaxed until the forces on each atom were below 0.05 eV/Å.

Ab initio molecular dynamics (AIMD) simulations were performed to calculate Li^+^ diffusivity and reveal its migration mechanism. These simulations used the NVT ensemble using a Nose–Hoover thermostat with a period of 80 fs^62^. The 1 × 1 × 2 supercells of Li_2_ZrCl_6_ and other structures have lattice parameters larger than 10 Å in each direction, and a Γ-centred 1 × 1 × 1 k-point grid was used. The heat-up process was executed for each supercell by raising the temperature from 100 K to each holding temperature (550−750 K) over 2 ps. AIMD simulations were conducted with a 2 fs interval time step for 200 ps at different holding temperatures, and diffusivities (D) were determined by linear fitting of the mean square displacement (MSD) of lithium ions using the following equations:

1 $${{{{{\rm{MSD}}}}}}=\frac{1}{{{{{{\rm{N}}}}}}}\mathop{\sum }\limits_{{{{{{\rm{i}}}}}}=1}^{{{{{{\rm{N}}}}}}}{\left|{{{{{{\rm{r}}}}}}}_{{{{{{\rm{i}}}}}}}\left({{{{{\rm{t}}}}}}+\triangle {{{{{\rm{t}}}}}}\right)-{{{{{{\rm{r}}}}}}}_{{{{{{\rm{i}}}}}}}({{{{{\rm{t}}}}}})\right|}^{2}$$

2 $${{{{{\rm{D}}}}}}=\frac{1}{2{{{{{\rm{dtN}}}}}}}\mathop{\sum }\limits_{{{{{{\rm{i}}}}}}=1}^{{{{{{\rm{N}}}}}}}{\left|{{{{{{\rm{r}}}}}}}_{{{{{{\rm{i}}}}}}}\left({{{{{\rm{t}}}}}}+\triangle {{{{{\rm{t}}}}}}\right)-{{{{{{\rm{r}}}}}}}_{{{{{{\rm{i}}}}}}}({{{{{\rm{t}}}}}})\right|}^{2}$$

where r_i_ is the position of the i^th^ ion at time t, Δt is the time step, N is the number of Li in the supercell structure, and d is the dimensionality factor. MSD and diffusivities were analysed by using the diffusion analyser module^63^ in Pymatgen^64^. Diffusion channel size analysis based on the Li-ion trajectories generated from AIMD simulations was utilized using the topological analysis package Zeo^++^^65^.

Electrochemical stability windows were evaluated by constructing grand potential phase diagrams of all relevant phases with compositions of Li_2_ZrCl_6_, Li_2_ZrCl_5_F, Li_2_ZrF_6_ and Li_3_Zr_4_F_19_ in equilibrium with the chemical potential of Li. The decomposition reaction energy is defined as follows:

3 $${\triangle {{{{{\rm{E}}}}}}}_{{{{{{\rm{D}}}}}}}={{{{{{\rm{E}}}}}}}_{{{{{{\rm{eq}}}}}}}({{{{{\rm{Phase\; equlibria}}}}}},\,{{{{{{\rm{\mu }}}}}}}_{{{{{{\rm{Li}}}}}}})-{{{{{{\rm{E}}}}}}}_{{{{{{\rm{SE}}}}}}}({{{{{\rm{phase}}}}}})-{\triangle {{{{{\rm{n}}}}}}}_{{{{{{\rm{Li}}}}}}}{{{{{{\rm{\mu }}}}}}}_{{{{{{\rm{Li}}}}}}}$$

where ${{{{{{\rm{\mu }}}}}}}_{{{{{{\rm{Li}}}}}}}$ is the chemical potential of Li and ${\triangle {{{{{\rm{n}}}}}}}_{{{{{{\rm{Li}}}}}}}$ is the number difference of elemental Li from the original composition. Interfacial chemical stability is calculated as the interface pseudobinary reaction energy between halide SE and contact material (cathode or sulfide SE), which is normalized by energy per formula unit of SE. The interface pseudobinary reaction energy is calculated as

4 $${\triangle {{{{{\rm{E}}}}}}}_{{{{{{\rm{rxn}}}}}}}({{{{{{\rm{C}}}}}}}_{{{{{{\rm{SE}}}}}}},\,{{{{{{\rm{C}}}}}}}_{{{{{{\rm{CM}}}}}}},\,{{{{{\rm{x}}}}}})={{{{{{\rm{E}}}}}}}_{{{{{{\rm{eq}}}}}}}({{{{{\rm{x}}}}}}{{{{{{\rm{C}}}}}}}_{{{{{{\rm{SE}}}}}}}+\left(1-{{{{{\rm{x}}}}}}\right){{{{{{\rm{C}}}}}}}_{{{{{{\rm{CM}}}}}}})-{{{{{\rm{xE}}}}}}({{{{{{\rm{C}}}}}}}_{{{{{{\rm{SE}}}}}}})-(1-{{{{{\rm{x}}}}}}){{{{{\rm{E}}}}}}({{{{{{\rm{C}}}}}}}_{{{{{{\rm{CM}}}}}}})$$

where x is the molar fraction of the SE with ${{{{{{\rm{C}}}}}}}_{{{{{{\rm{SE}}}}}}}\,{{{{{\rm{and}}}}}}\,{{{{{{\rm{C}}}}}}}_{{{{{{\rm{CM}}}}}}}$ which are the compositions of halide SE and contact material (cathode or sulfide SE), respectively. For a given composition, the most stable convex hull energy (the lowest energy of the phase equilibrium) was used. The crystal structures of LiCoO_2_ (space group: *R*$\bar{3}$*m*), Li_6_PS_5_Cl (space group: *F*$\bar{4}$3*m*) and all known compounds belonging to the Li-Zr-Cl-F systems were obtained from the Materials Project database^66^, and their energies were calculated using the same DFT calculation parameters.

### Electrochemical characterization

Ionic conductivities were measured by the AC impedance method using ion-blocking Ti|SE|Ti symmetric cells. The cold-pressed pellets (40 mg, ≈500 μm) with a diameter of 6 mm were prepared at 370 MPa. The measurements were conducted 3 h after cell fabrication to ensure that thermal equilibrium was achieved. The EIS data were recorded for cells under an external pressure of ~70 MPa at open circuit voltage with an amplitude of 10 mV and a frequency range from 10 mHz to 7 MHz using a VSP-300 (Bio-Logic). Ten data points in each decade in frequency were recorded. For the Li-In||LiCoO_2_ or LiNi_0.88_Co_0.11_Mn_0.01_O_2_ cells, Li-In was used as the counter and reference electrodes. After the Li-In powders with a nominal composition of Li_0.5_In were prepared by ball-milling of In (Aldrich, 99%) and Li (FMC Lithium Corp.) at 2000 rpm, they were then mixed with Li_6_PS_5_Cl powders in a weight ratio of 8:2 at 2000 rpm. For the CV measurements, two kinds of SE powders (Li_2_ZrCl_6_ and ZrO_2_-2Li_2_ZrCl_5_F) were manually mixed with super C65 with a weight ratio of 10:1. Li_6_PS_5_Cl powders (150 mg) were pelletized under 100 MPa to form SE layers. The SE-super C65 (Wellcos Co., Korea, BET surface area = 62 m^2^ g^−1^) mixture and Li-In electrodes were attached on either side of the SE layers, and the whole assembly was pressed at 370 MPa. The CV measurements were conducted using VMP3 (Bio-Logic) with a scan range from open-circuit voltage to 5 V (vs. Li/Li^+^) at 0.1 mV s^−1^. All-solid-state cells were fabricated as follows. For the preparation of the LPSCl monolayer, LPSCl powders (150 mg, ≈600 μm) were pelletized under 100 MPa. For the preparation of the bilayer, a thinner layer of ZrO_2_-2Li_2_ZrCl_5_F (30 mg) was placed on the LPSCl layer and pelletized under 100 MPa. Composite working electrodes were prepared from a mixture of LiCoO_2_ (Wellcos Co., Korea, D50 = 15.5 μm) or single-NCM88 (EcoPro BM, Korea, D50 = 3.3 μm), HNSEs, and super C65 powders with a weight ratio of 70:30:3 or 50:50:3. Finally, the LiCoO_2_ or single-NCM88 electrodes (40-50 μm) and the Li-In electrodes (≈130 μm) were attached on either side of the SE layers, and the whole assembly was pressed at 370 MPa. The all-solid-state cells were tested under an external pressure of ~70 MPa. The specific current and specific capacity were determined based on the mass of active material, which was 10.2 mg for LCO and 7.3 mg for S-NCM88. The EIS measurements for the cells were performed from 1.5 MHz to 5 mHz at an amplitude of 10 mV after discharging the cells to 3.8 V (vs. Li/Li^+^) at 16.4 mA g^−1^ at the 2nd, 10th, and 100th cycles. To maintain a constant temperature during electrochemical tests, the cells were placed within an incubator at 30 °C. To ensure the reproducibility of the results, at least three cells were employed for each electrochemical test.

### Reporting summary

Further information on research design is available in the Nature Portfolio Reporting Summary linked to this article.

## Supplementary information

Supplementary Information

Peer Review File

Reporting Summary

## Data Availability

The data generated or analysed in this study are provided in the paper and Supplementary Information and available from the corresponding author on reasonable request.
