# Supplementary material for: Boosting the interfacial superionic conduction of halide solid electrolytes for all-solid-state batteries
Source: Nat Commun. 2023 Apr 28;14:2459. doi: 10.1038/s41467-023-38037-z (PMC10147626; doi:10.1038/s41467-023-38037-z)
Supplement: Supplementary file 1 — Supplementary Information [file 41467_2023_38037_MOESM1_ESM.pdf]

[Supplementary Information]

Boosting the interfacial superionic conduction of halide solid electrolytes for all-solid-state batteries

Hiram Kwak,[a1] Jae-Seung Kim,[b1] Daseul Han,[c1] Jong Seok Kim,[a] Juhyoun Park,[a] Gihan Kwon,[d] Seong-Min Bak,[d] Unseon Heo,[c] Changhyun Park,[b] Hyun-Wook Lee,[b] Kyung-Wan Nam,[c] Dong-Hwa Seo,*[b] and Yoon Seok Jung*[a]*

[a] *Department of Chemical and Biomolecular Engineering, Yonsei University, Seoul 03722, South Korea*

[b] *School of Energy and Chemical Engineering, Ulsan National Institute of Science and Technology (UNIST), Ulsan, 44919, South Korea*

[c] *Department of Energy and Materials Engineering, Dongguk University, Seoul, 04620, South Korea*

[d] *National Synchrotron Light Source II, Brookhaven National Laboratory, Upton, New York 11973, United States*

[1] *These authors contributed equally: Hiram Kwak, Jae-Seung Kim, Daseul Han.*

Corresponding Authors: *e-mail: knam@dongguk.edu (Prof. K.-W. Nam), dseo@unist.ac.kr (Prof. D.-H. Seo), yoonsjung@yonsei.ac.kr (Prof. Y. S. Jung)

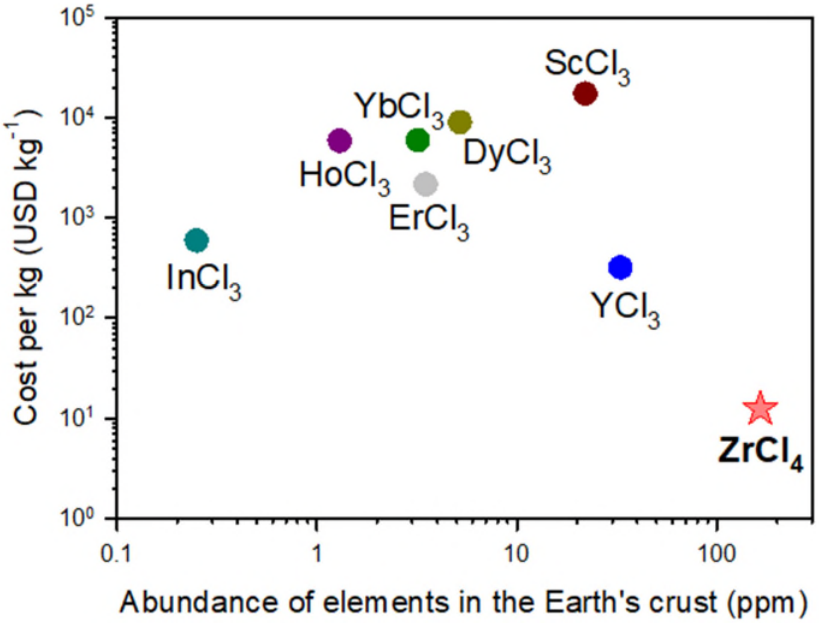

Supplementary Fig. 1 Cost and abundance of metal halides. Estimated bulk prices and abundance in the Earth's crust for metal halide precursors for halide SEs.[1,2]

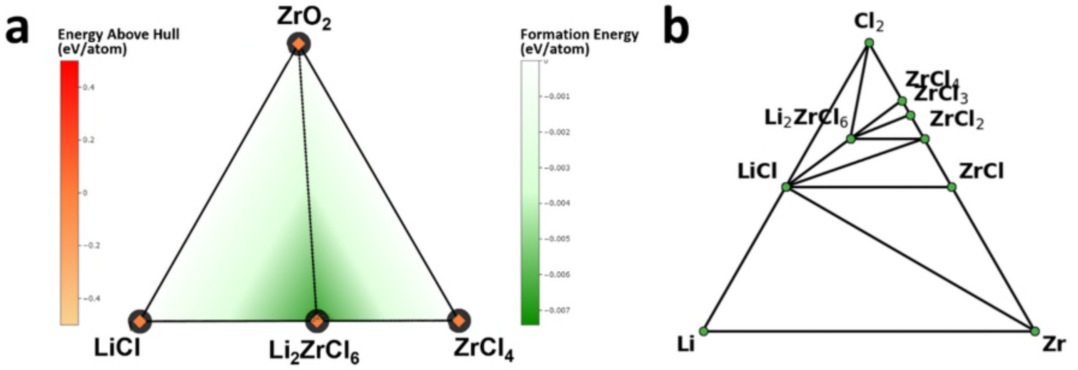

Supplementary Fig. 2 a,b Ternary phase diagrams of ZrO_2-$ZrCl_4$-LiCl compound (**a**) and Li-Zr-Cl (**b**).

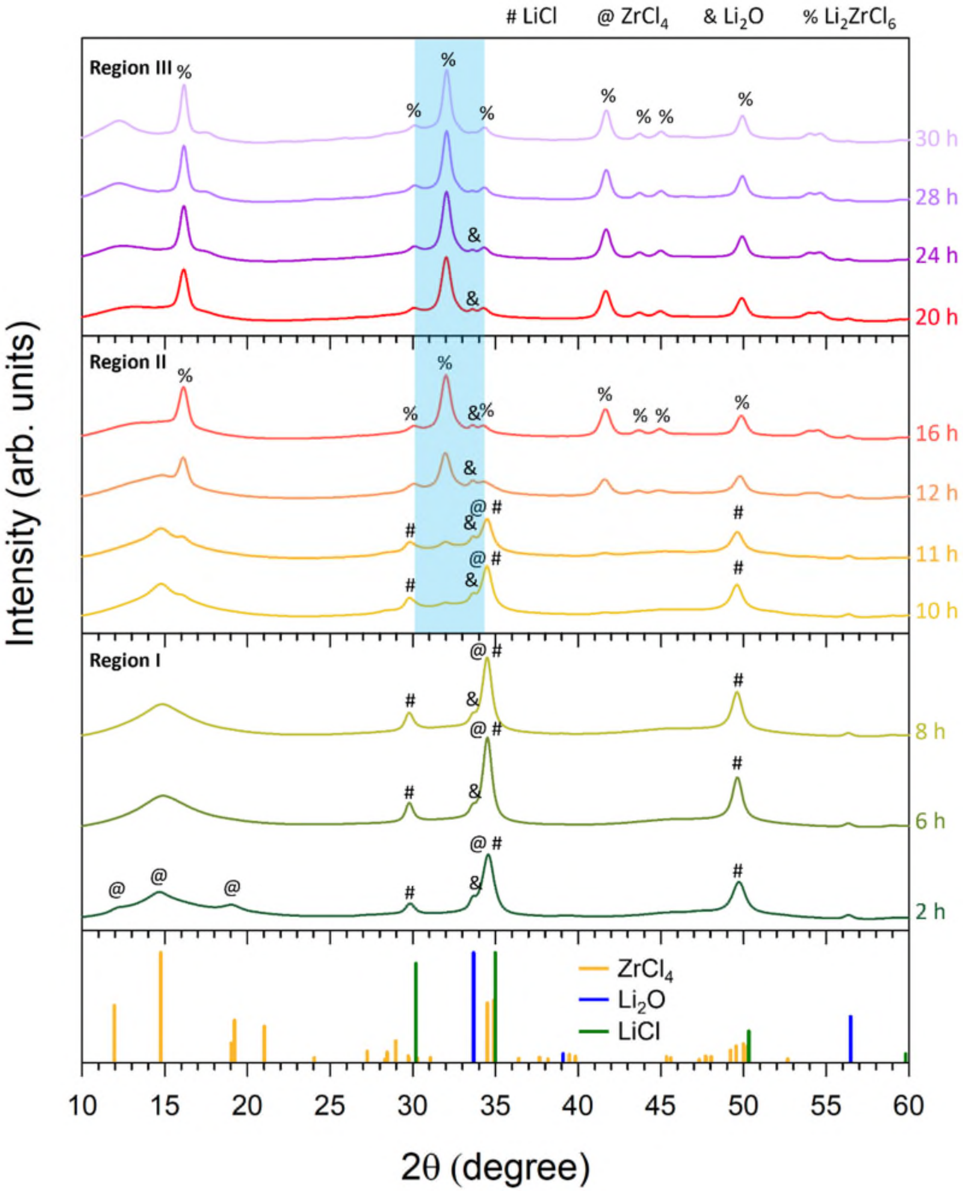

Supplementary Fig. 3 Characterization of synthesis reaction mechanism for HNSE by Synchrotron XRD. Synchrotron XRD patterns for the precursor mixture of Li_2O and $ZrCl_4$ (2:3 molar ratio) with varying ball-milling time.

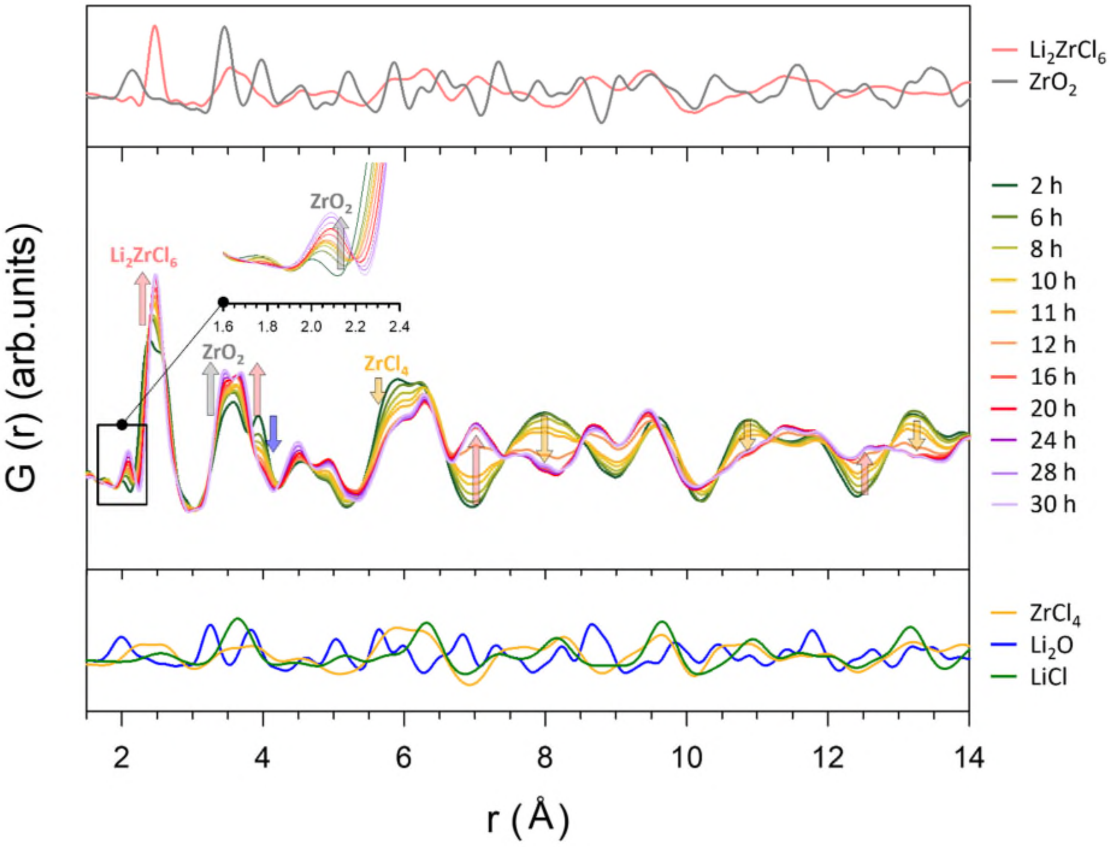

Supplementary Fig. 4 Characterization of the synthesis reaction mechanism for HNSE by PDF. Synchrotron PDF G(r) for the precursor mixture of Li_2O and $ZrCl_4$ (2:3 molar ratio) as a function of ball-milling time.

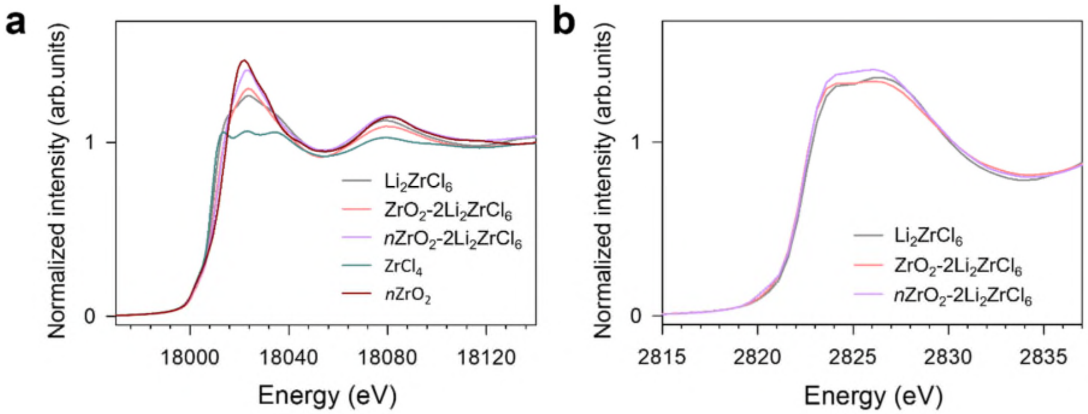

Supplementary Fig. 5 XANES results. a,b Zr K-edge (**a**) and Cl K-edge (**b**) XANES spectra of Li_2ZrCl_6, ZrO_2-$2Li_2ZrCl_6$, and *n*ZrO_2-$2Li_2ZrCl_6$. The reference samples spectra for $ZrCl_4$ and ZrO_2 nanopowders (*n*ZrO_2) are also compared in (**a**).

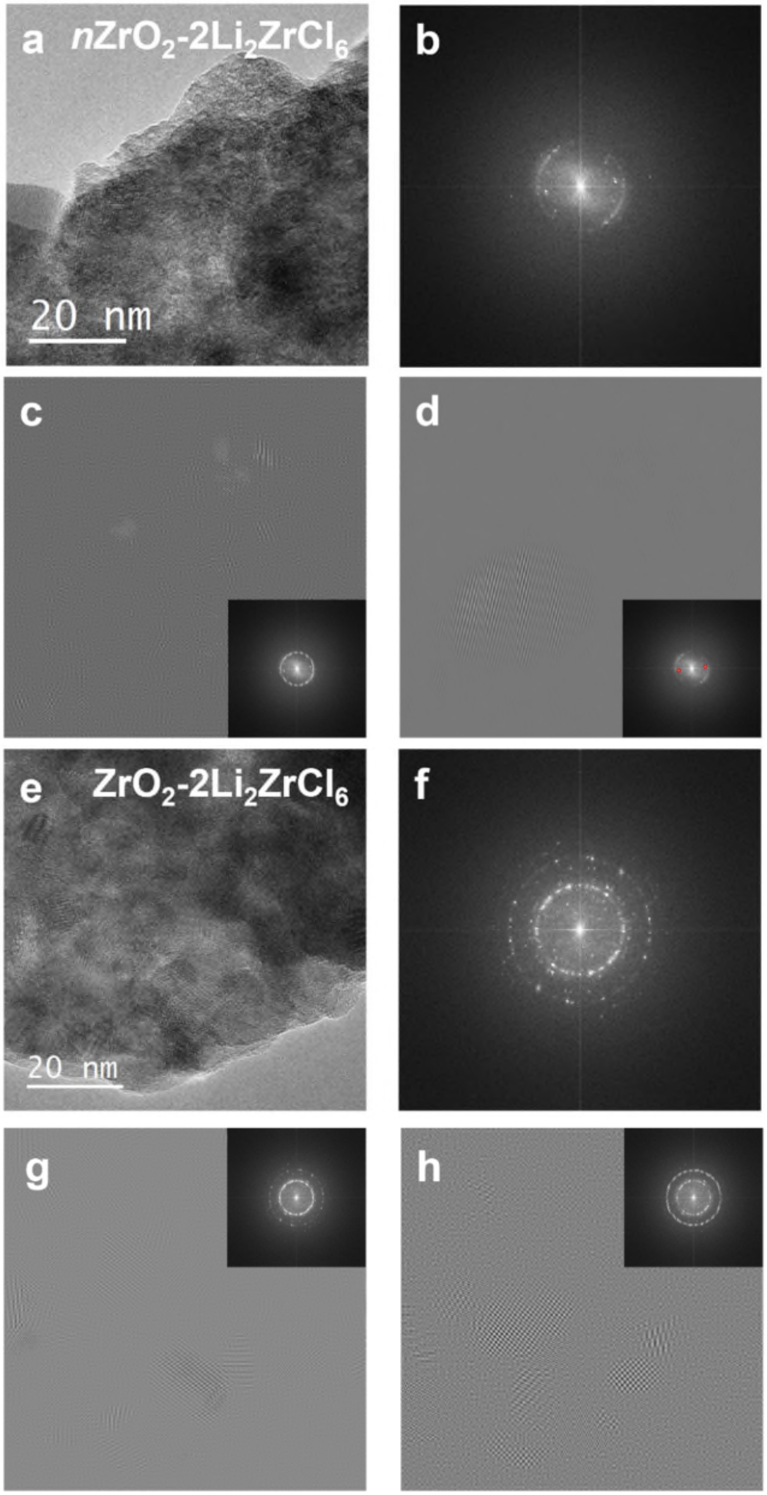

Supplementary Fig. 6 Cryo-HRTEM results of Li^+ HNSEs. a-d, Cryo-HRTEM image of $n$$ZrO_2$-$2Li_2ZrCl_6$ (**a**) and corresponding FFT patterns (**b**). **c**,**d,** Inverse FFT images from the circled region of the FFT pattern in the inset image, corresponding to Li_2ZrCl_6 (**c**) and ZrO_2 (**d**). **e-h**, Cryo-HRTEM image of ZrO_2-$2Li_2ZrCl_6$ (**e**) and corresponding FFT patterns (**f**). **g**, **h** Inverse FFT images from the circled region of the FFT pattern in the inset image, corresponding to Li_2ZrCl_6 (**g**) and ZrO_2 (**h**).

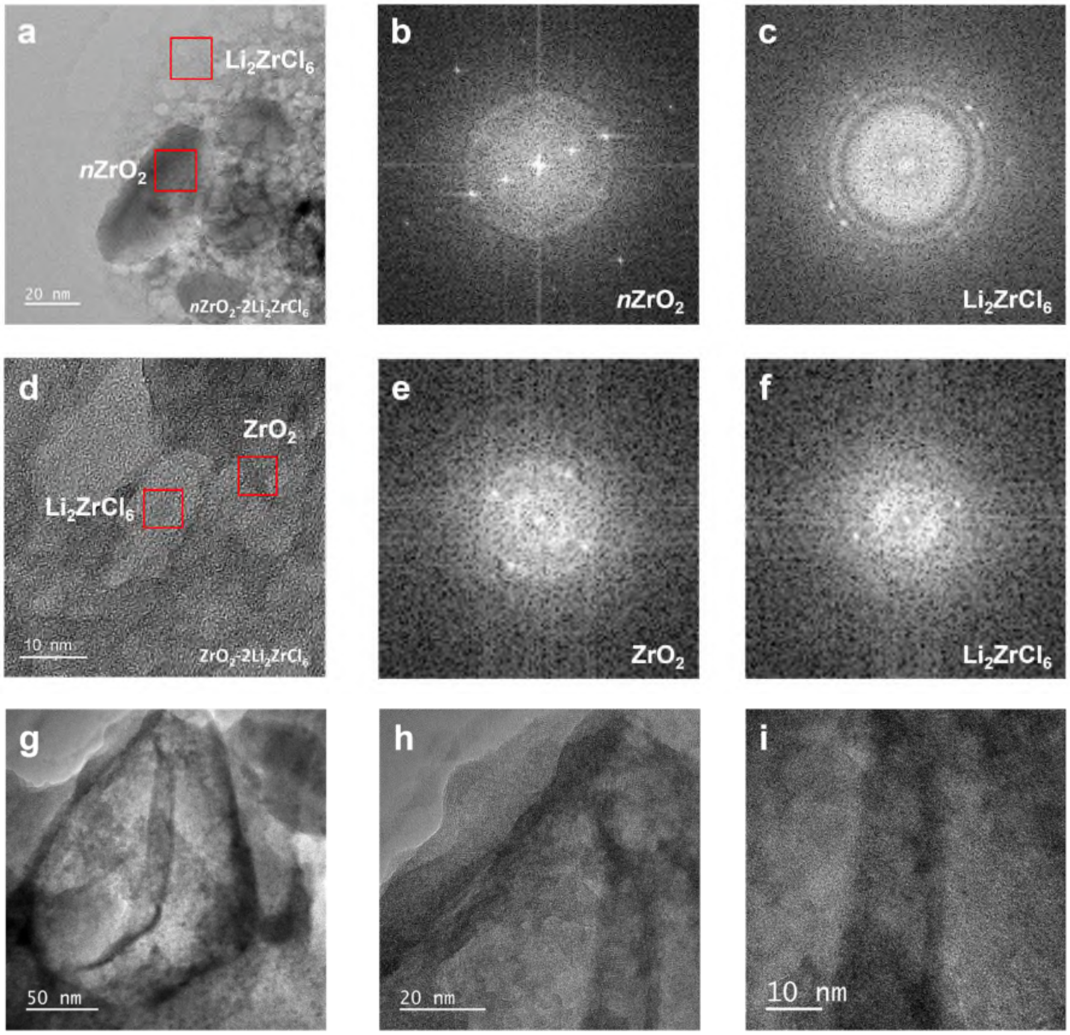

Supplementary Fig. 7 HRTEM results of Li$^+$ HNSEs. a-c, HRTEM image of *n*ZrO_2-$2Li_2ZrCl_6$ (**a**) and corresponding FFT patterns for *n*ZrO_2 (**b**) and Li_2ZrCl_6 (**c**). **d-f**, HRTEM image of ZrO_2-$2Li_2ZrCl_6$ (**d**) and corresponding FFT patterns of ZrO_2 (**e**) and Li_2ZrCl_6 (**f**). **g-i**, High-resolution TEM images of ZrO_2-$2Li_2ZrCl_6$ at different magnifications.

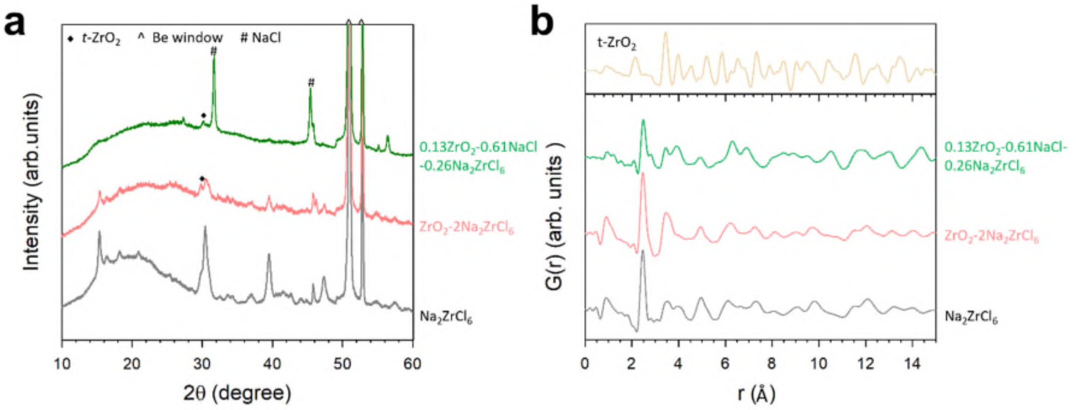

Supplementary Fig. 8 Characterization of Na^+ HNSEs. a,**b**, XRD patterns (**a**) and PDF G(r) (**b**) for Na_2ZrCl_6, ZrO_2-$2Na_2ZrCl_6$, and $0.13ZrO_2$-$0.61NaCl$-$0.26Na_2ZrCl_6$.

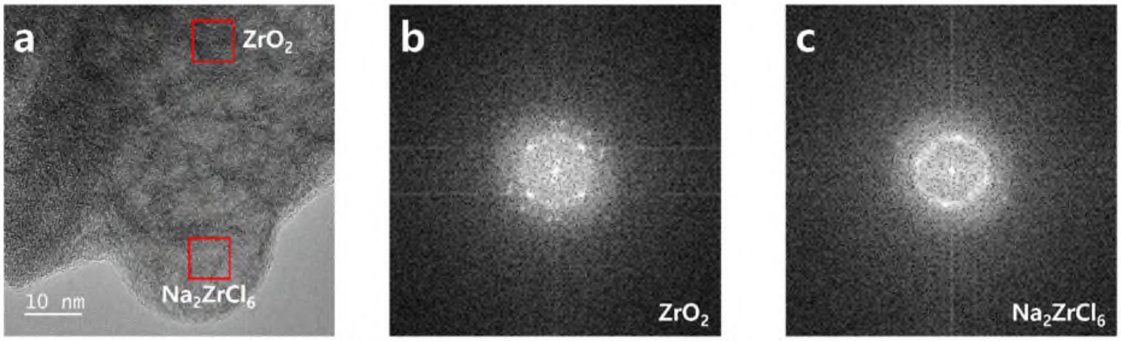

Supplementary Fig. 9 HRTEM results for Na^+ HNSEs. a, HRTEM image of ZrO_2-$2Na_2ZrCl_6$. **b**,**c**, Corresponding FFT patterns of ZrO_2 (**b**) and Na_2ZrCl_6 (**c**).

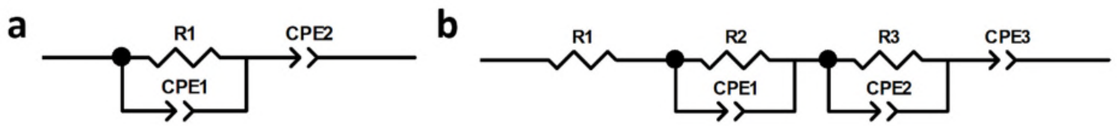

Supplementary Fig. 10 Equivalent circuit models for Ti|SE|Ti symmetric cells and Li–In||$LiCoO_2$ ASSB cells. a, Equivalent circuit moldel employed to fit the raw data presented in Figures 2a, 2b, 4b, and Supplementary Figures 12a, 23a, 24b. **b,** Equivalent circuit model employed to fit the raw data presented in Supplementary Figure 31.

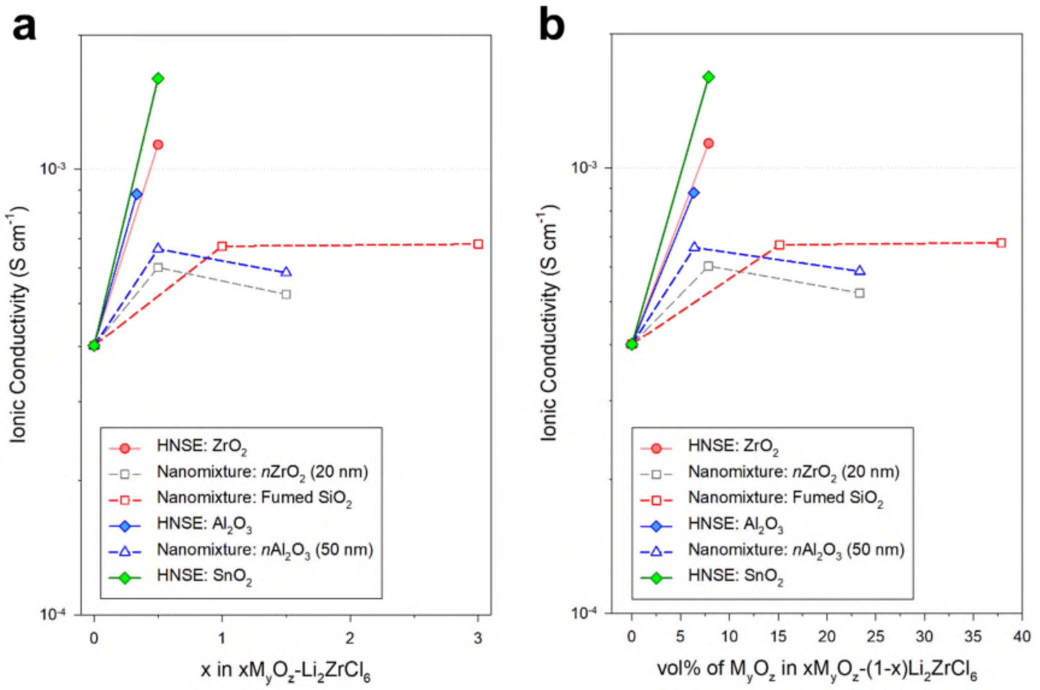

Supplementary Fig. 11 Li^+ conductivities for M_yO_z-Li_2ZrCl_6 HNSEs vs. nanomixtures. **a**,**b**, Li^+ conductivities for HNSEs prepared by the in situ mechanochemical method using Li_2O compared with those for nanomixture samples prepared by mechanical mixing with M_yO_z nanoparticles as a function of the mole fraction of M_yO_z (**a**) and volume % of M_yO_z (**b**). The measurements of Li^+ conductivities were conducted using ion-blocking Ti|SE|Ti symmetric cells at 30 °C.

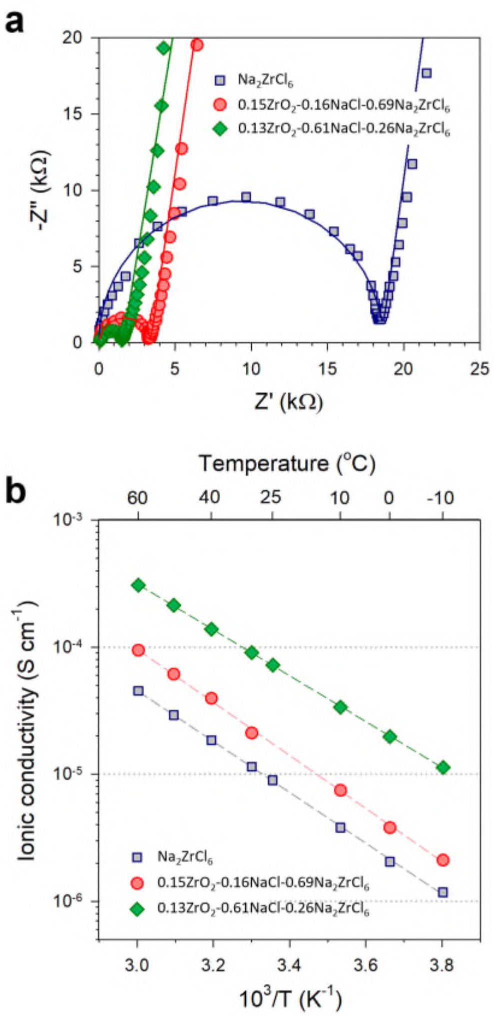

Supplementary Fig. 12 Na^+ conductivity results of Na^+ HNSEs. a, Nyquist plots of ion-blocking Ti|SE|Ti symmetric cells at 30 °C for Na^+ HNSEs (0.15ZrO_2-0.16NaCl-0.69Na_2ZrCl_6 and 0.13ZrO_2-0.61NaCl-0.26Na_2ZrCl_6) and Na_2ZrCl_6. The symbols and lines correspond to the raw data and fitted results obtained from the equivalent circuit model, respectively, as described in Supplementary Figure 10. The fitted result of EIS is summarized in Supplementary Table 4. **b**, Corresponding Arrhenius plots.

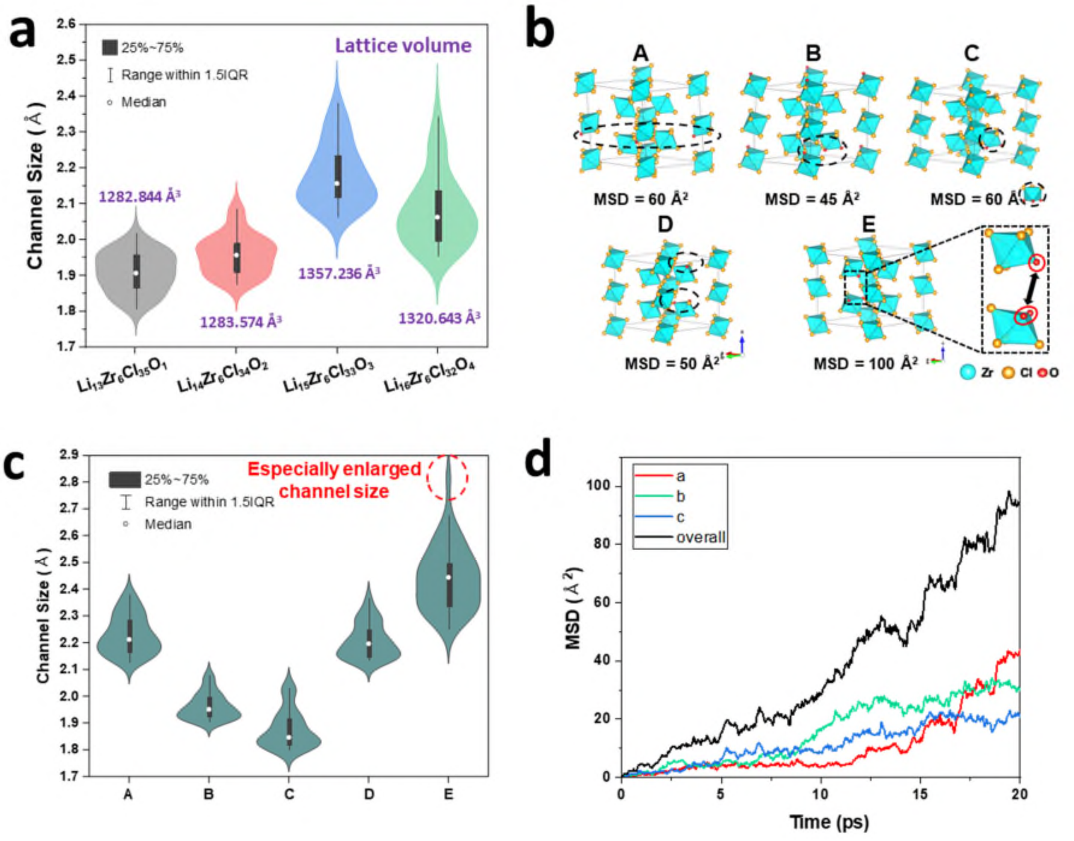

Supplementary Fig. 13 Screening process of oxygen-substituted Li_2ZrCl_6 (LZC) and $Li_{2.5}ZrCl_{5.5}O_{0.5}$. a, Topological analysis and lattice volume of the most stable oxygen-substituted structures of LZC ($Li_{12}Zr_6Cl_{36} \rightarrow Li_{12+x}Zr_6Cl_{36-x}O_x$, x = 1~4) for determining O-substituted composition. **b-c**, Several screened structures among screened 50 structures (**b**) in the composition of $Li_{15}Zr_6Cl_{33}O_3$ (= $Li_{2.5}ZrCl_{5.5}O_{0.5}$) with mean square displacement (MSD) value obtained by short AIMD screening (700 K, 20 ps) and Li^+ transport channel size of each structure (**c**). **d**, Short AIMD screening during 20 ps at 700 K for crystal structure E.

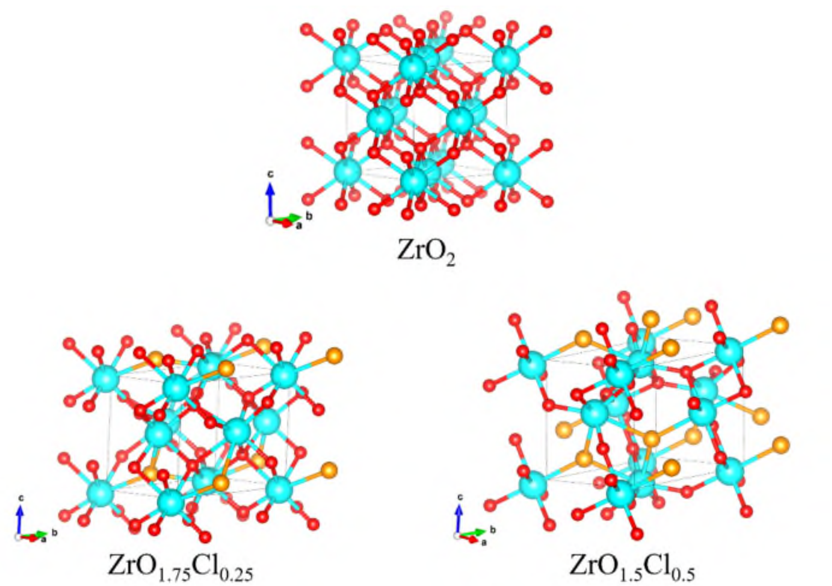

Supplementary Fig. 14 Crystal structures of ZrO_2, $ZrO_{1.75}Cl_{0.25}$, and $ZrO_{1.5}Cl_{0.5}$. (Light blue: zirconium, red: oxygen, yellow: chloride)

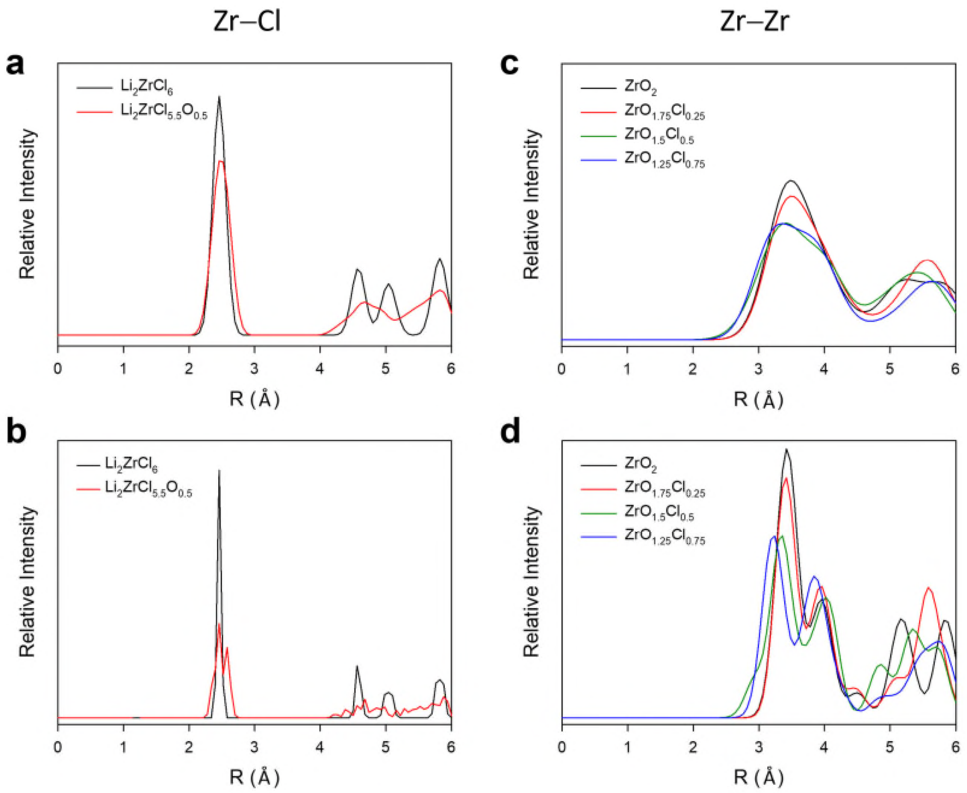

Supplementary Fig. 15 Simulated radial distribution function (RDF) results. a,b Simulated RDF of the calculated structures for Zr-Cl peak of Li_2ZrCl_6 and $Li_2ZrCl_{5.5}O_{0.5}$ with higher smoothing factor (**a**) and with lower smoothing factor (**b**). Simulated RDF of the calculated structures for Zr-Zr peaks of ZrO_2, $ZrO_{1.75}Cl_{0.25}$, $ZrO_{1.5}Cl_{0.5}$ and $ZrO_{1.25}Cl_{0.75}$ with higher smoothing factor (**c**) and with lower smoothing factor (**d**). Broad Zr-Zr peak at approximately 3.5 Å suggests local anion substitution of the interface between Li_2ZrCl_6 and ZrO_2 under high-energy ball-milling conditions.

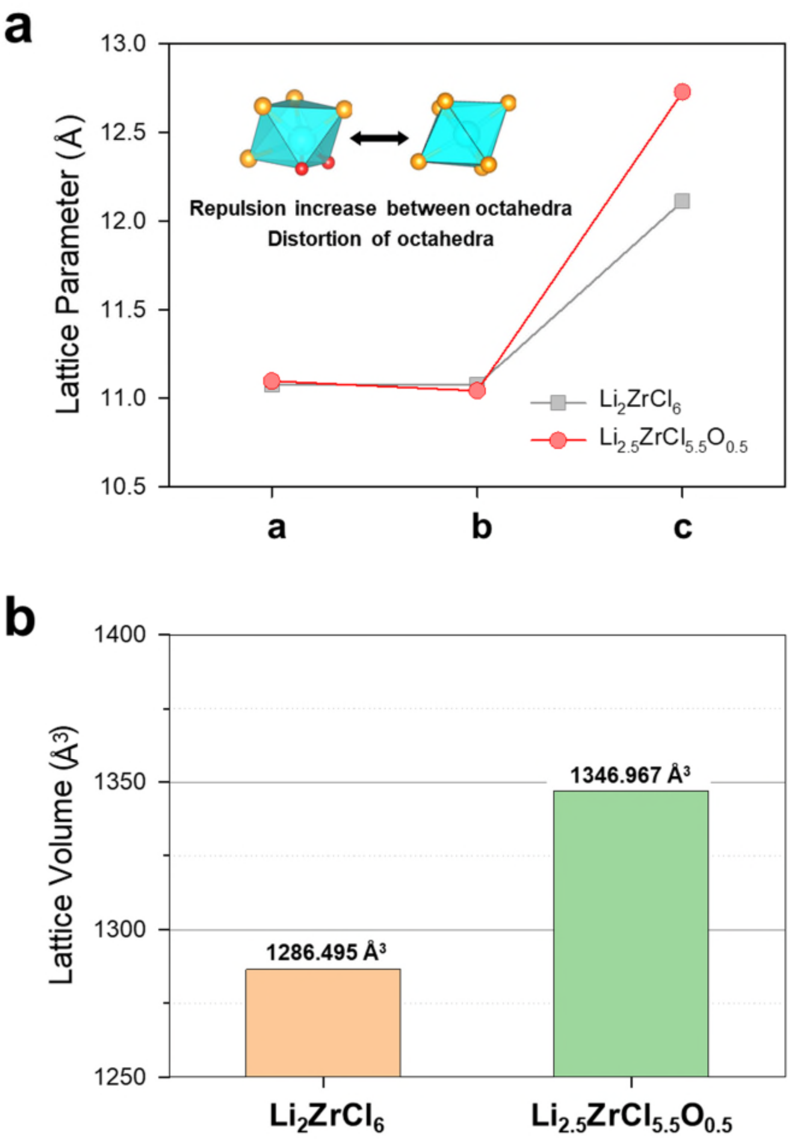

Supplementary Fig. 16 Differences in the lattice between Li_2ZrCl_6 (LZC) and $Li_{2.5}ZrCl_{5.5}O_{0.5}$ (LZCO). a,b, Lattice parameters (**a**) and lattice volume (**b**) of LZC and LZCO.

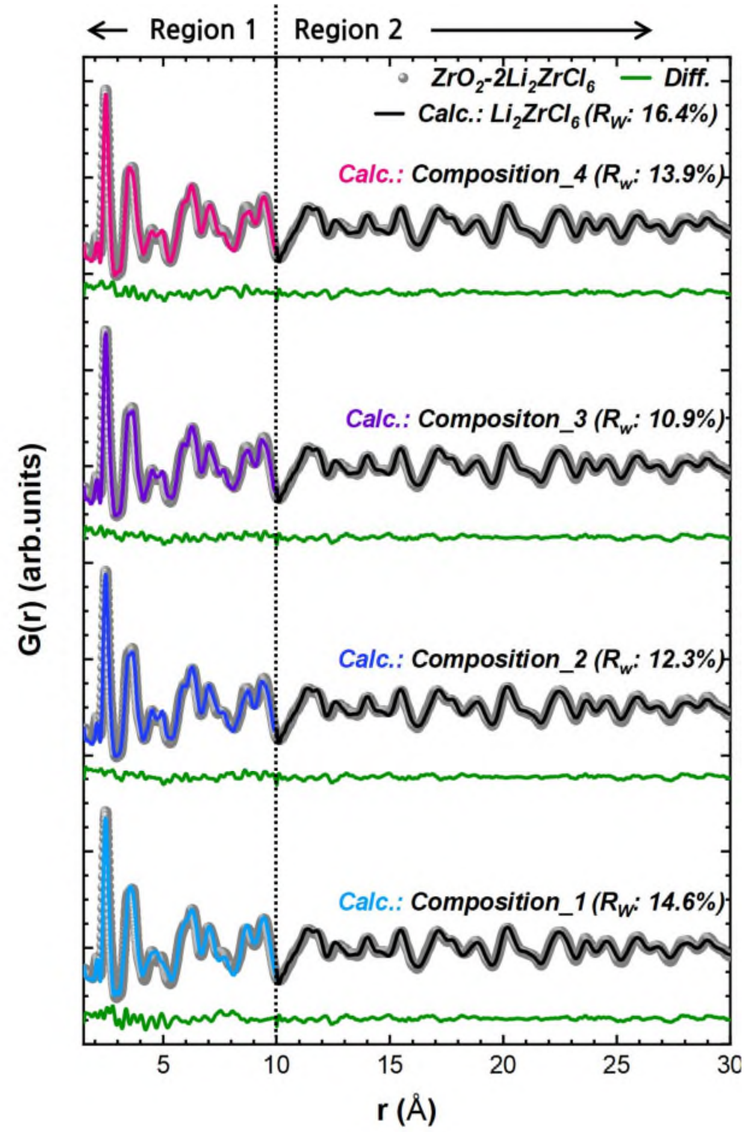

Supplementary Fig. 17 Experimental PDF with best-fit results for ZrO_2-$2Li_2ZrCl_6$ in the 1.5–30 Å range. The PDF fitting was performed across different refinement ranges (low r range of 1.5–10 Å; high r range of 10–30 Å)

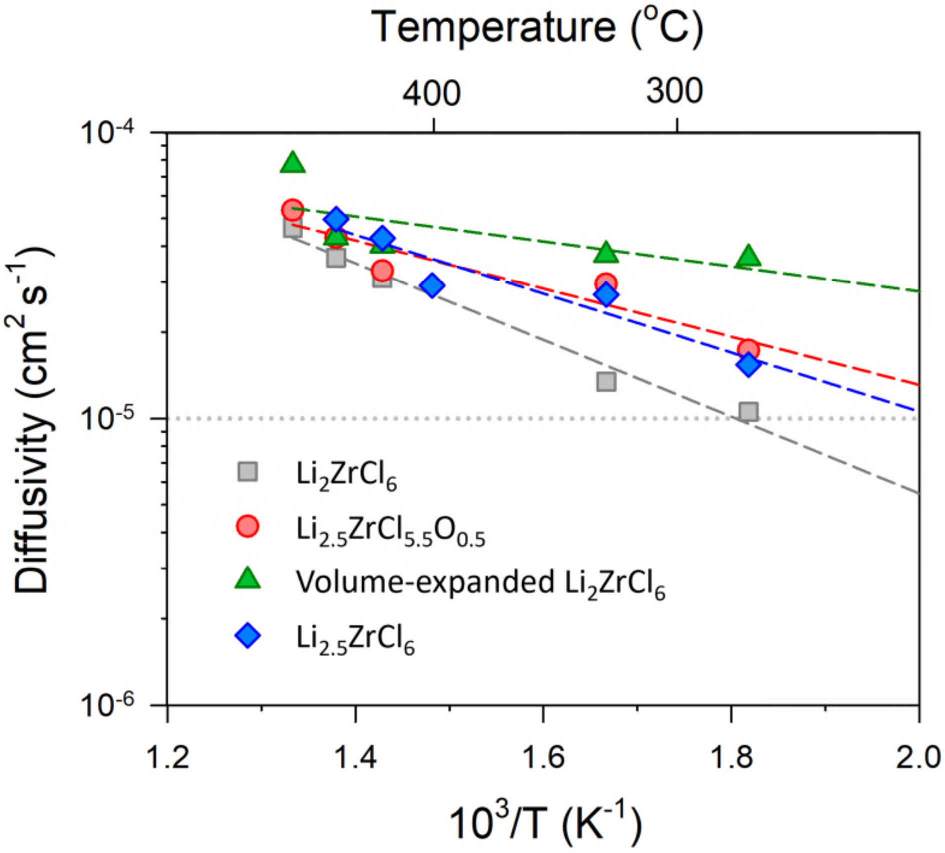

Supplementary Fig. 18 Interfacial Li^+ superionic conduction in HNSEs. Arrhenius plots with higher magnification of the AIMD simulation shown in Figure 3c.

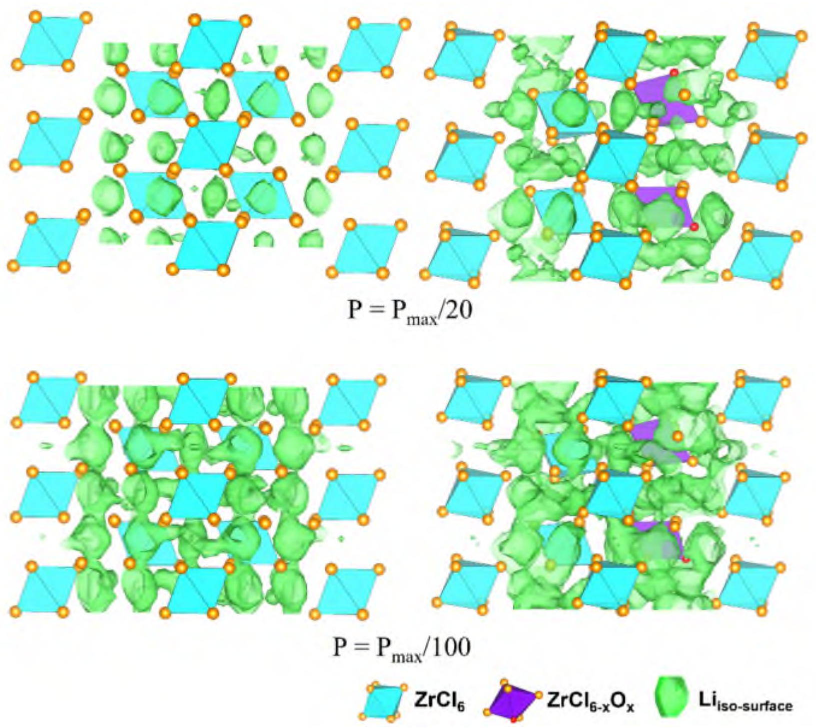

Supplementary Fig. 19 Li probability density at 600 K in ~200 ps (isosurface value $P = P_{max}/20$ & $P = P_{max}/100$)

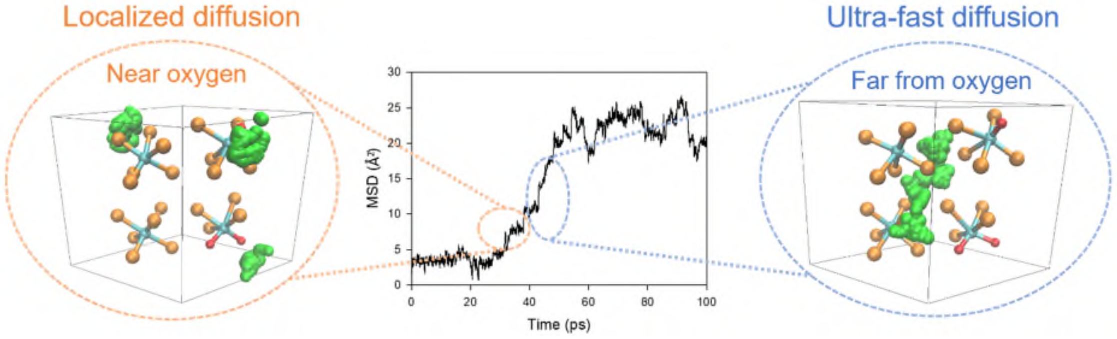

Supplementary Fig. 20 Traced individual Li^+ trajectory of LZCO from 30 ps to 40 ps and from 40 ps to 50 ps. MSD is the mean square distance. (Light blue: zirconium, red: oxygen, yellow: chloride, green: lithium)

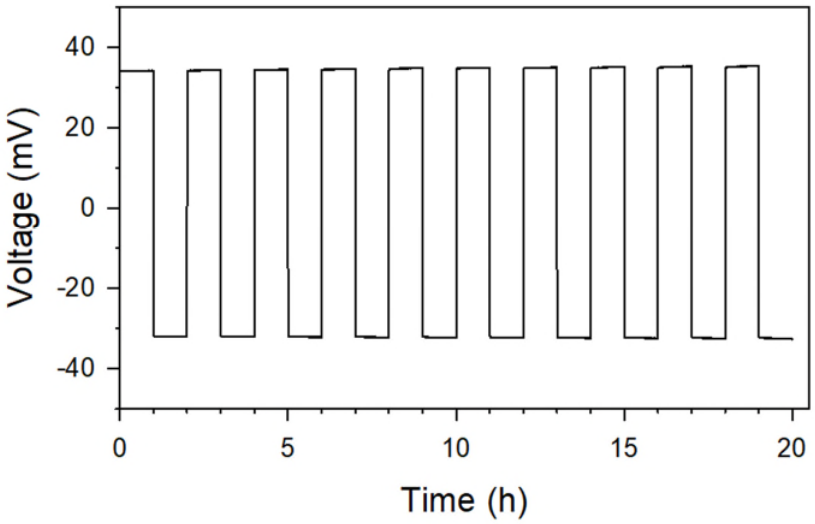

Supplementary Fig. 21 Cycling test for ^{6}Li exchange NMR measurements. Voltage profiles obtained by continuous charging and discharging at a constant current of 0.5 mA for ^{6}Li|LPSCl|(ZrO_2-2Li_2ZrCl_6)|LPSCl|^{6}Li cells at 60 °C.

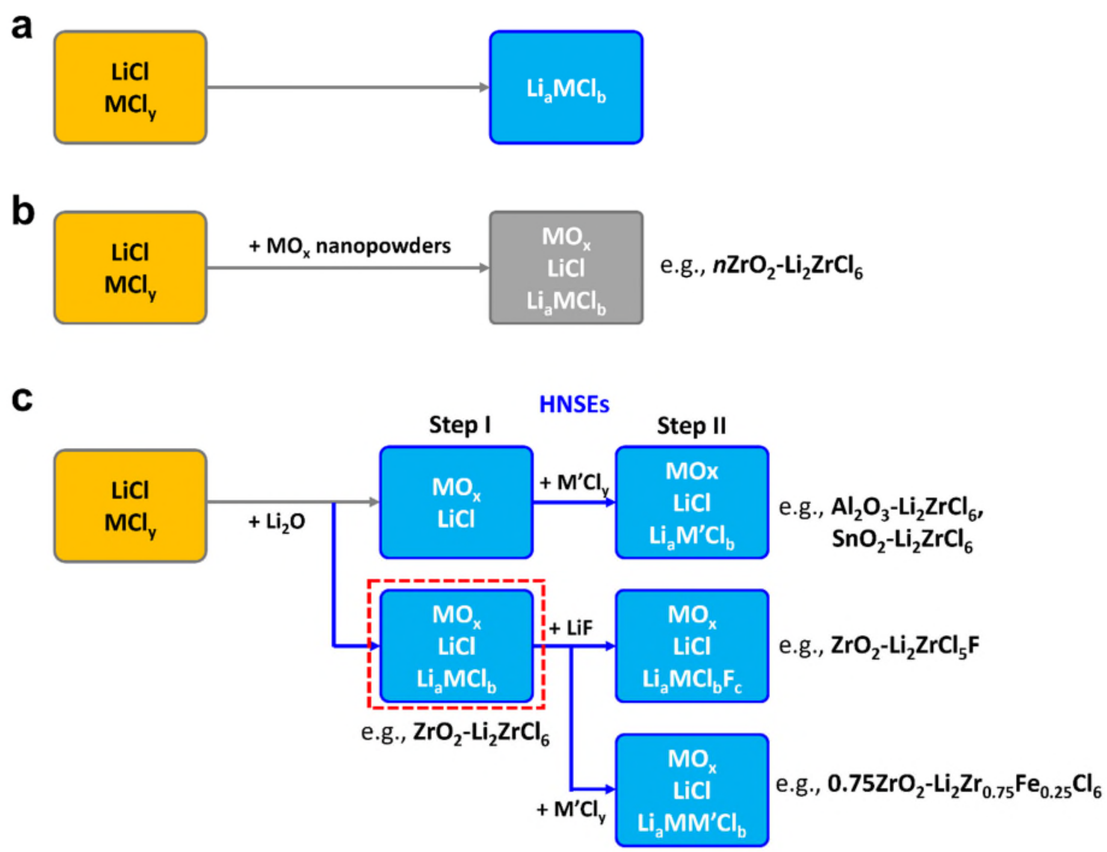

Supplementary Fig. 22 Preparation protocols of HNSEs. a, Conventional preparation protocol of halide SEs. **b**, Preparation protocol for nanomixture samples using metal oxide nanoparticles. **c**, Mechanochemical HNSE preparation protocols using Li_2O. (M = metal elements such as Al, Fe, Sc, Y, In, Ln, Zr, Hf, x = 2-3, y = 3-4)

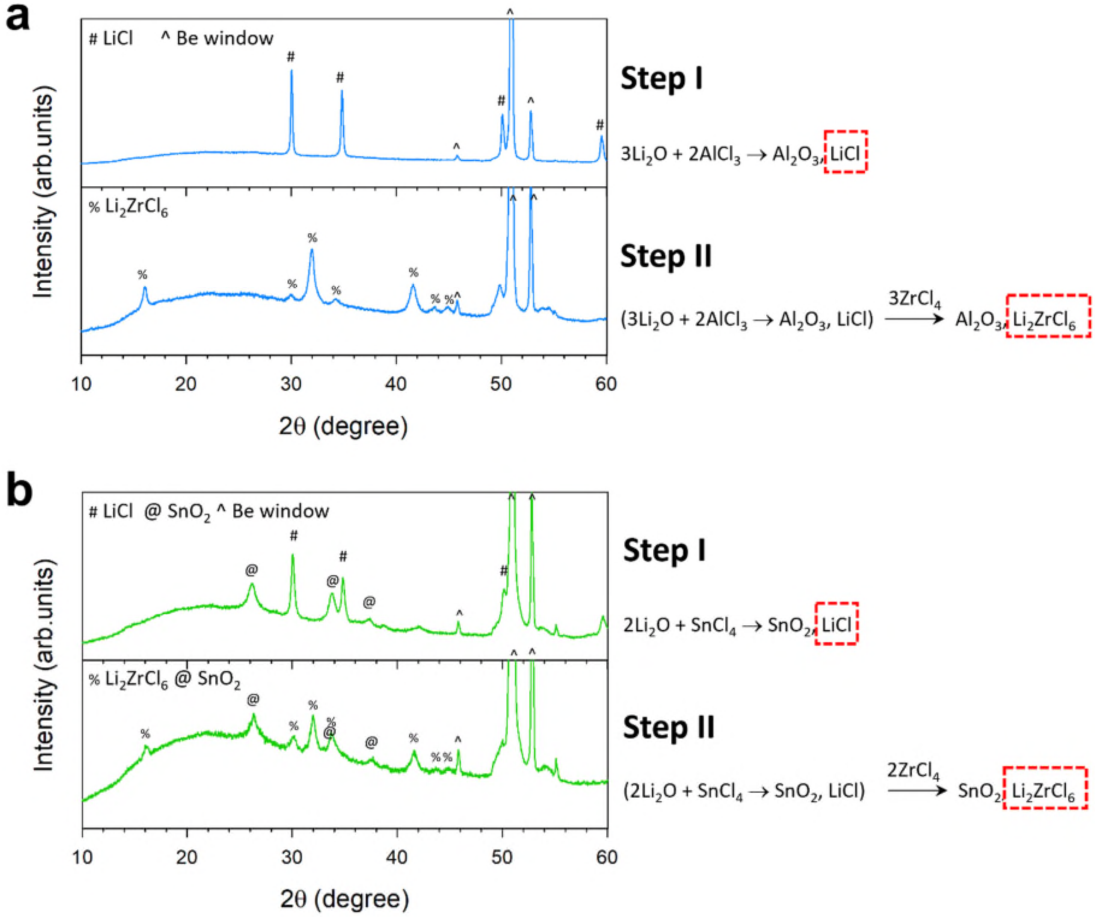

Supplementary Fig. 23 Two-step preparation of Al_2O_3-$3Li_2ZrCl_6$ and SnO_2-$2Li_2ZrCl_6$ HNSEs. a, XRD patterns for Al_2O_3, LiCl (top, step I) and Al_2O_3-$3Li_2ZrCl_6$ (bottom, step II). **b,** XRD patterns for SnO_2, LiCl (top, step I) and SnO_2-$2Li_2ZrCl_6$ (bottom, step II).

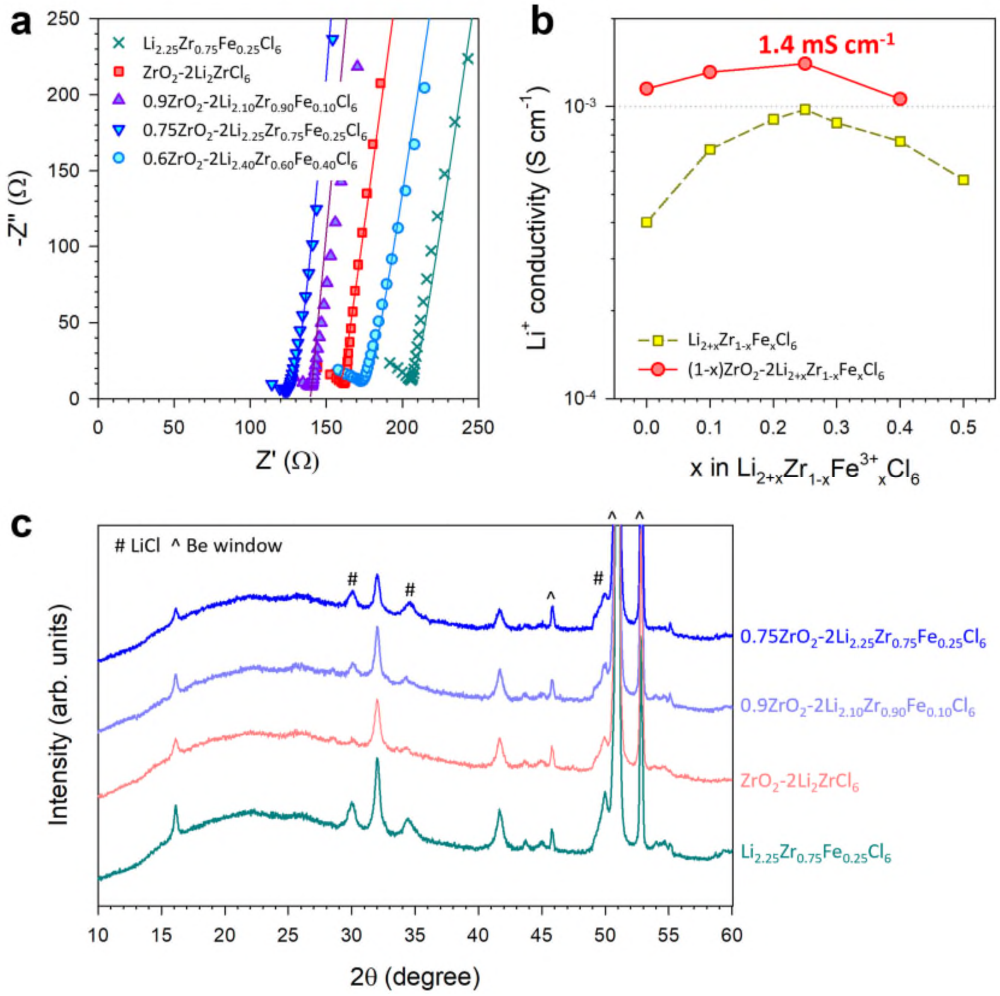

Supplementary Fig. 24 Two-step preparation of (1-*x*)ZrO_2-$2Li_{2+x}Zr_{1-x}Fe_xCl_6$ HNSEs. a-c, Nyquist plots of ion-blocking Ti|SE|Ti symmetric cells at 30 °C (**a**), Li^+ conductivities at 30 °C (**b**), and XRD patterns (**c**) for $Li_{2.25}Zr_{0.75}Fe_{0.25}Cl_6$ and (1-*x*)ZrO_2-$2Li_{2+x}Zr_{1-x}Fe_xCl_6$. The symbols in (**a**) represent the raw data, and the lines correspond to the fitted results obtained from the equivalent circuit model, as described in Supplementary Figure 10. The fitted result of EIS is summarized in Supplementary Table 4.

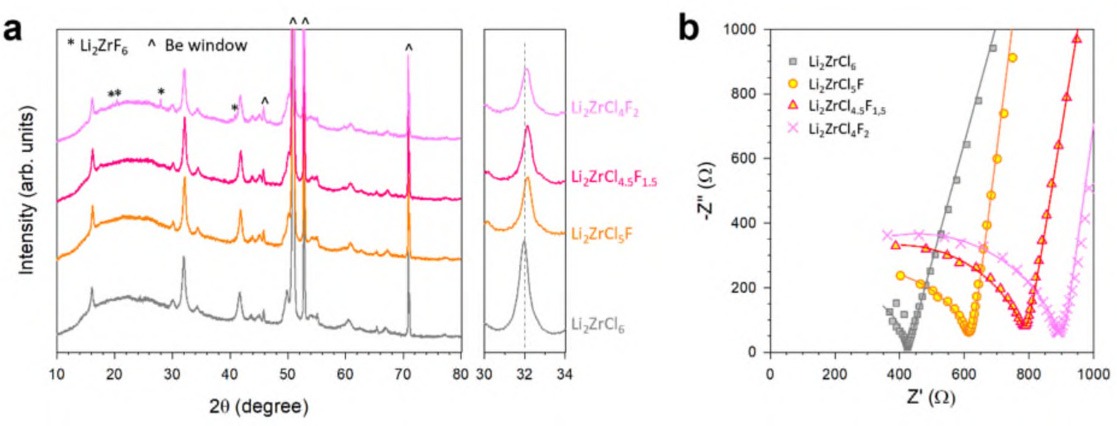

Supplementary Fig. 25 F-substituted Li_2ZrCl_6. a,b, XRD patterns (**a**) and Nyquist plots of ion-blocking Ti|SE|Ti symmetric cells at 30 °C (**b**) for a series of $Li_2ZrCl_{6-x}F_x$. The symbols in the Nyquist plots represent the raw data, and the lines correspond to the fitted results obtained from the equivalent circuit model, as described in Supplementary Figure 10. The fitted result of EIS is summarized in Supplementary Table 4.

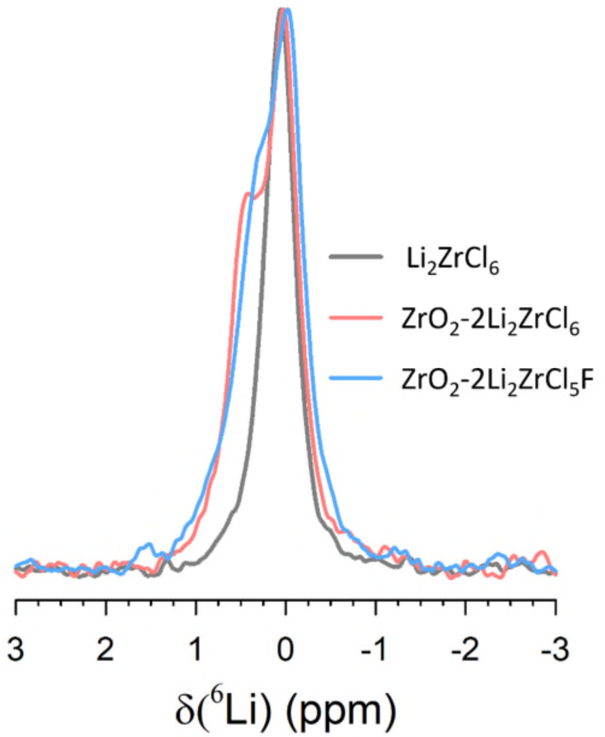

Supplementary Fig. 26 ^{6}Li MAS-NMR spectrum for ZrO_2-2Li_2ZrCl_5F. Spectra for Li_2ZrCl_6 and ZrO_2-2Li_2ZrCl_6 are also compared.

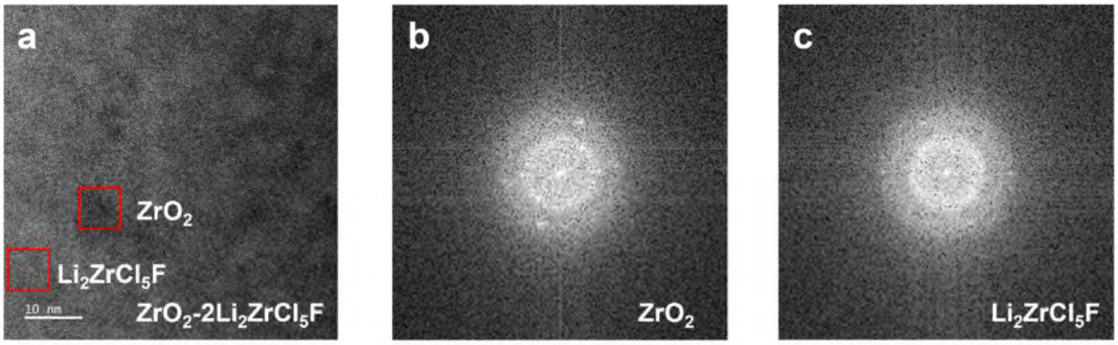

Supplementary Fig. 27 HRTEM results for F-substituted HNSE. a, HRTEM image of ZrO_2-$2Li_2ZrCl_5F$. **b**,**c**, Corresponding FFT patterns of ZrO_2 (**b**) and Li_2ZrCl_5F (**c**).

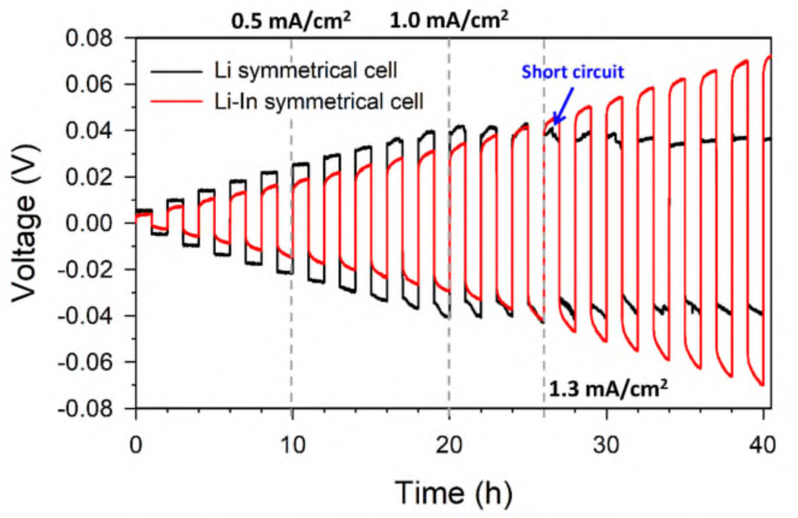

Supplementary Fig. 28 Galvanostatic cycling of Li|LPSCl|Li and Li-In|LPSCl|Li-In symmetrical cells with current increment of 0.1 mA cm^{-2} at 30 ºC. All-solid-state cells with a diameter of 13 mm, comprising Ti rods as the current collectors and a polyaryletheretherketone mold were used. To fabricate Li||Li and Li-In||Li-In symmetrical cells, an LPSCl layer was formed by pelletizing 150 mg of LPSCl by pressing at 370 MPa for 3 min. For Li||Li cells, Li metal with a diameter of 12 mm was attached to each side of the SE (LPSCl) layer and the entire assembly was pressed at a pressure of 30 MPa. For Li-In||Li-In cells, $Li_{0.5}In$ electrode were placed on each side of the SE layer and the entire assembly was pressed at a pressure of 370 MPa.

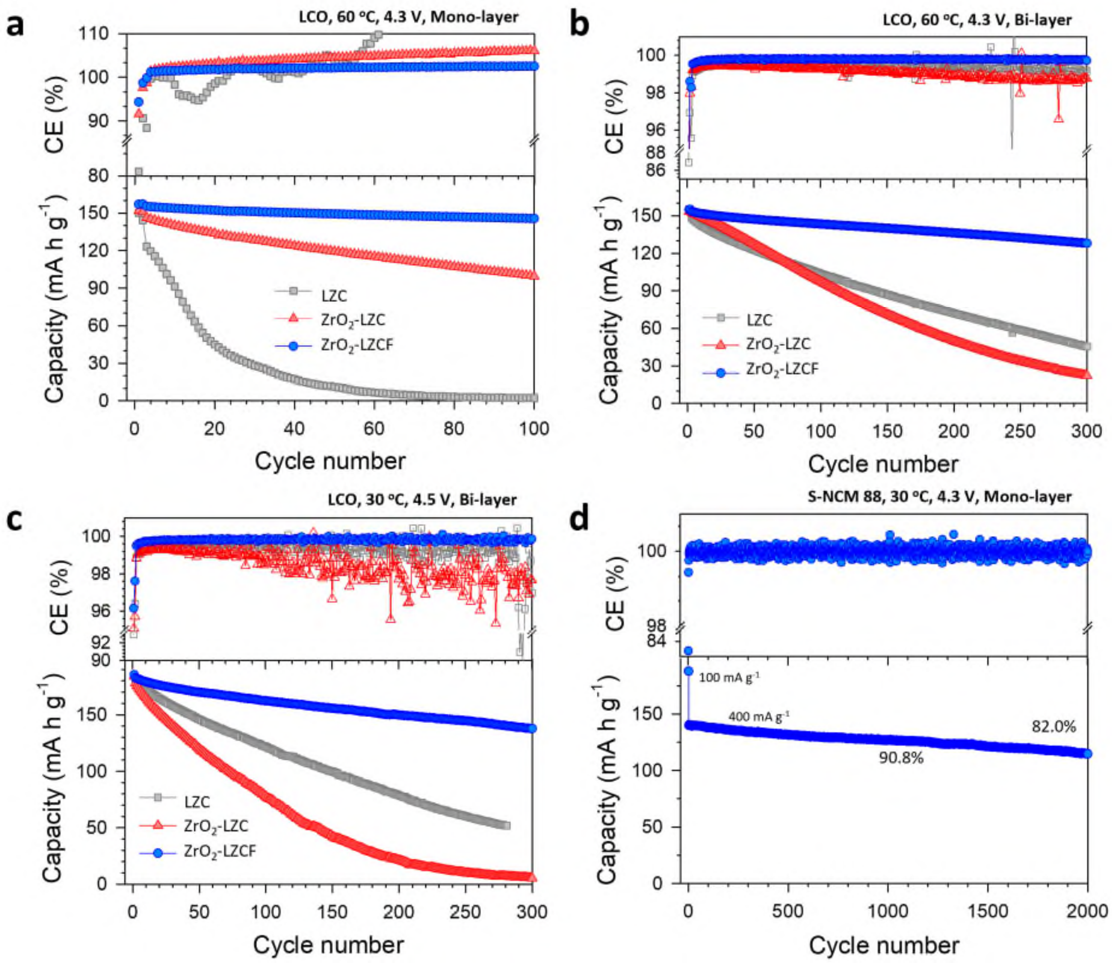

Supplementary Fig. 29 Coulombic efficiency (CE) of LCO and S-NCM88 ASSB cells employing HNSEs (ZrO_2-$2Li_2ZrCl_6$ and ZrO_2-$2Li_2ZrCl_5F$ vs. Li_2ZrCl_6) a, CE for LCO electrodes with an LPSCl monolayer, cycled up to 4.3 V (vs. Li/Li^+) at 82.0 mA g^{-1}. **b**,**c,** CE for LCO electrodes with a ZrO_2-LZCF bilayer, cycled up to 4.3 V at 60 °C and 82 mA g^{-1} (**b**) and 4.5 V (vs. Li/Li^+) at 30 °C and 82.0 mA g^{-1} (**c**). **d,** CE for S-NCM88 electrode using ZrO_2-LZCF at 400 mA g^{-1} and 30 °C. The specific current and capacity were determined based on the mass of active material (10.2 mg for LCO and 7.3 mg for S-NCM88). All the cells were cycled under a pressure of 70 MPa.

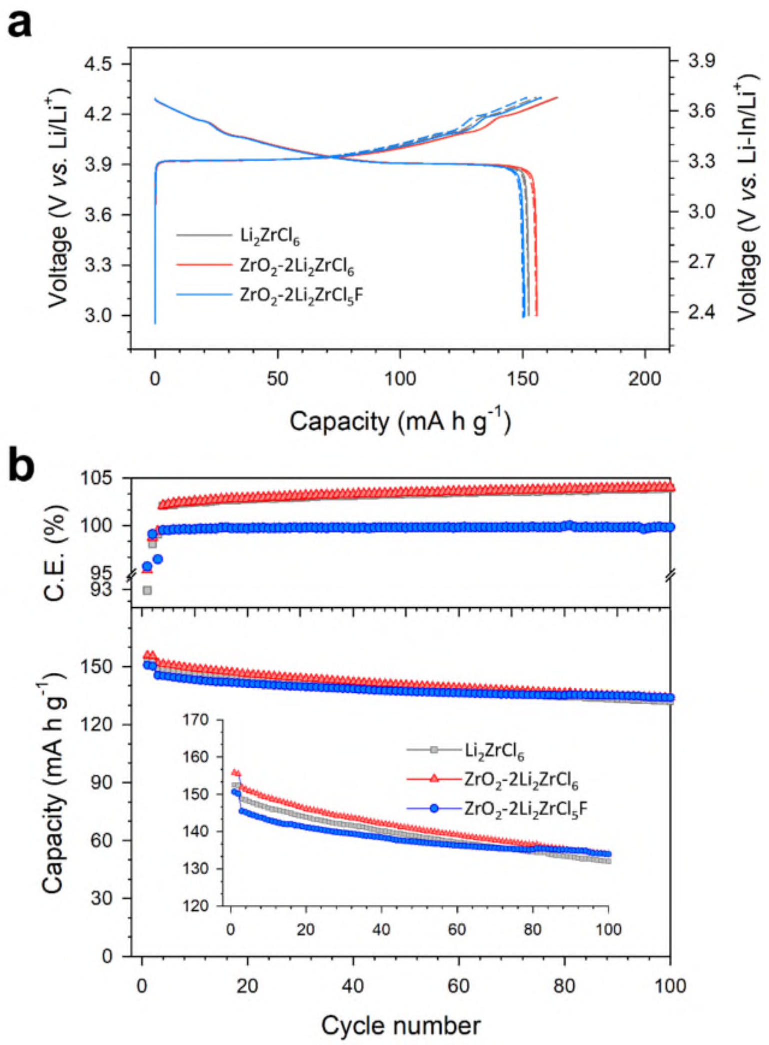

Supplementary Fig. 30 Li–In||LiCoO2 ASSB cells with LPSCl monolayers at 30 °C. a, First-cycle charge–discharge voltage profiles at 16.4 mA g^{-1} for LCO electrodes using LZC, ZrO_2-LZC, and ZrO_2-LZCF. **b**, Corresponding cycling performances with Coulombic efficiency at 82.0 mA g^{-1}. The first two cycles were tested at 16.4 mA g^{-1} for (**b**). The specific current and capacity were determined based on the mass of active material (10.2 mg for LCO and 7.3 mg for S-NCM88). All the cells were cycled under a pressure of 70 MPa.

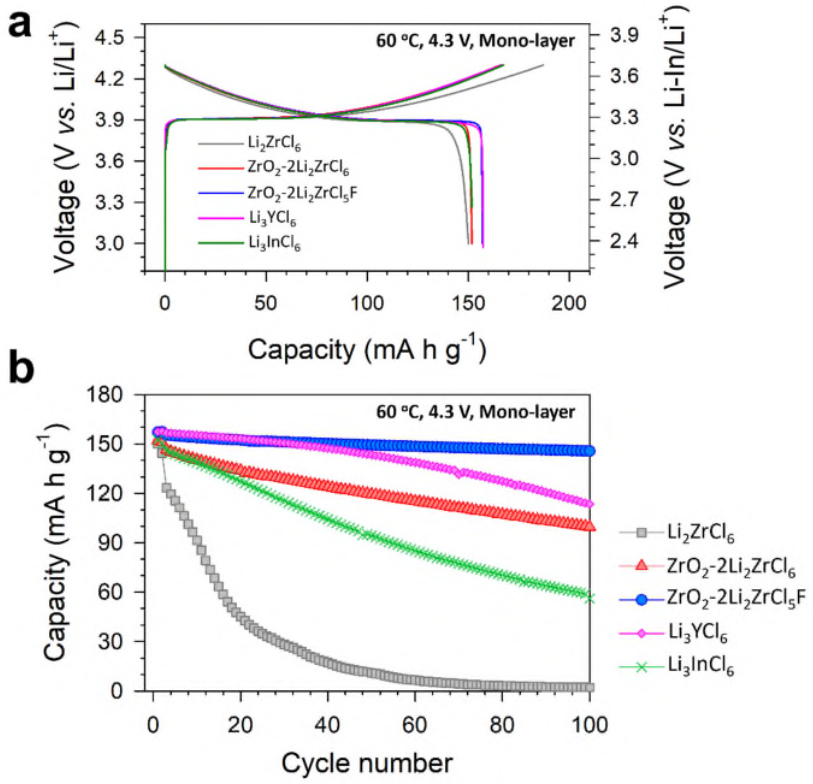

Supplementary Fig. 31 Li–In||$LiCoO_2$ ASSB cells with LPSCl monolayers at 60 °C. a, First-cycle charge–discharge voltage profiles at 16.4 mA g^{-1} for $LiCoO_2$ electrodes using LZC, ZrO_2-LZC, ZrO_2-LZCF, Li_3YCl_6, and Li_3InCl_6. **b**, Corresponding cycling performances at 82.0 mA g^{-1}. The first two cycles were tested at 16.4 mA g^{-1} for (**b**). The specific current and capacity were determined based on the mass of active material (10.2 mg for LCO and 7.3 mg for S-NCM88). All the cells were cycled under a pressure of 70 MPa.

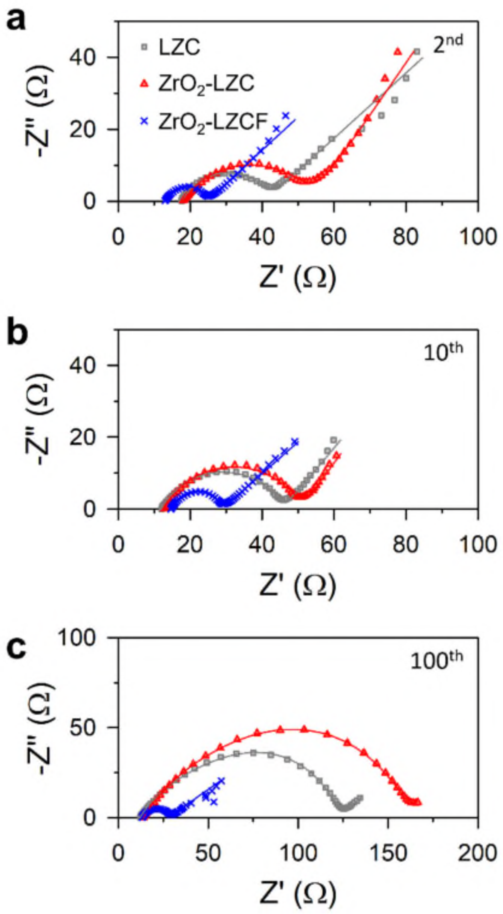

Supplementary Fig. 32 EIS results of Li–In||LiCoO$_2$ ASSB cells with (ZrO$_2$-LZCF) |LPSCl bilayers at 60 °C. a-c, Nyquist plots for the electrodes using LZC, ZrO$_2$-LZC and ZrO$_2$-LZCF at the 2nd cycle (**a**), 10th cycle (**b**), and 100th cycle (**c**). The symbols represent the raw data, and the lines correspond to the fitted results obtained from the equivalent circuit model, as described in Supplementary Figure 10b. The fitted result of EIS is summarized in Supplementary Table 16.

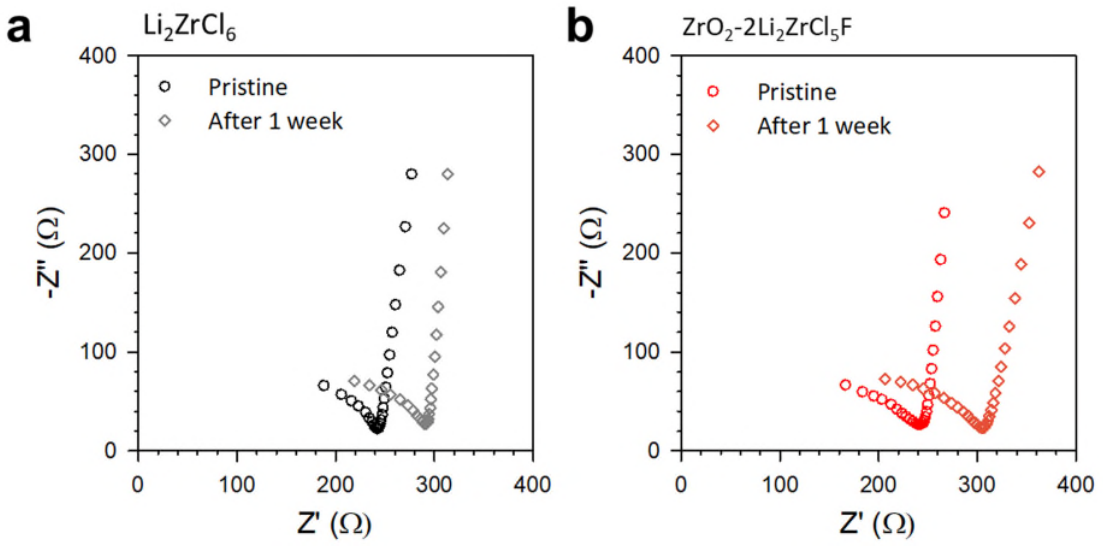

Supplementary Fig. 33 Static compatibility of halide Ses with sulfide SE LPSCl. A,**b**, Nyquist plots of Ti|(halide-LPSCl mixture)|Ti cells stored at 60 °C for a week for Li_2ZrCl_6 (**a**) and ZrO_2-$2Li_2ZrCl_5F$ (**b**).

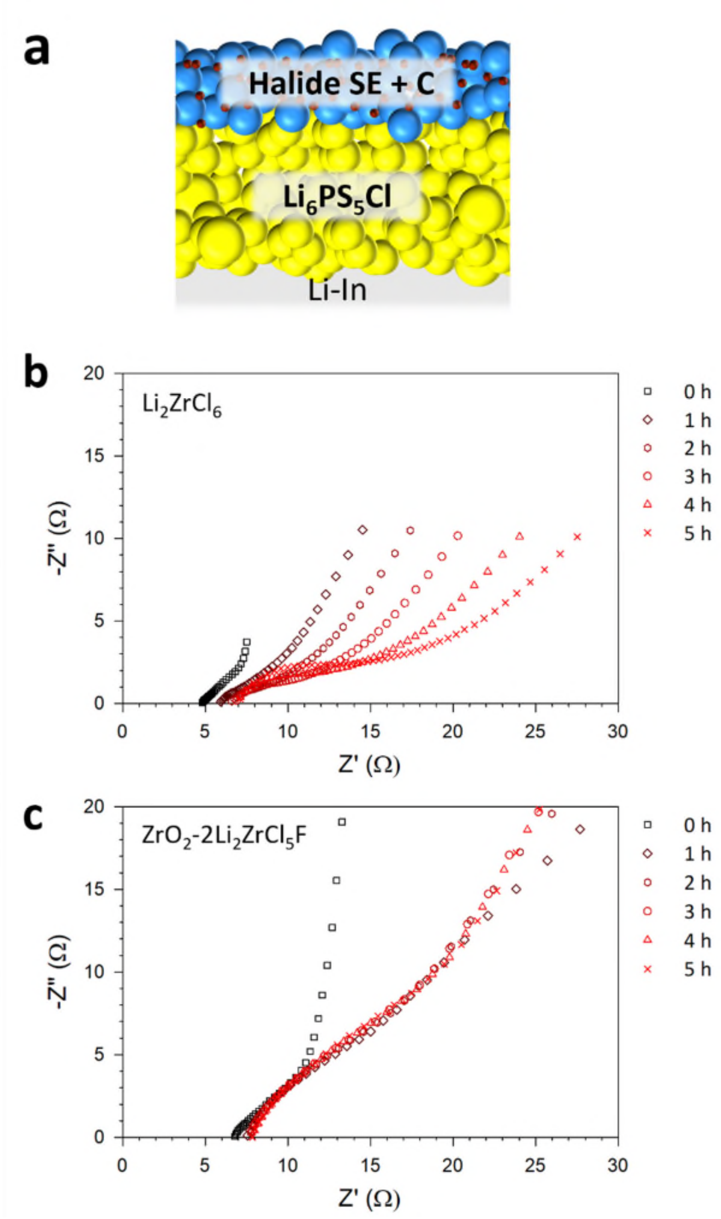

Supplementary Fig. 34 Compatibility of halide Ses with sulfide SE LPSCl under applied voltages. A, Schematic of a Li-In|LPSCl|halide SE-carbon cell. The cells were subjected to an applied voltage of 4.3 V (vs. Li/Li^+) at 60 °C for the duration. **B**,**c**, Corresponding Nyquist plots for Li_2ZrCl_6 (**b**) and ZrO_2-$2Li_2ZrCl_5F$ (**c**) as a function of time. These experiments were conducted in a potentiostatic mode, wherein a cell voltage of 4.3 V (vs. Li/Li^+) was maintained throughout the analysis.

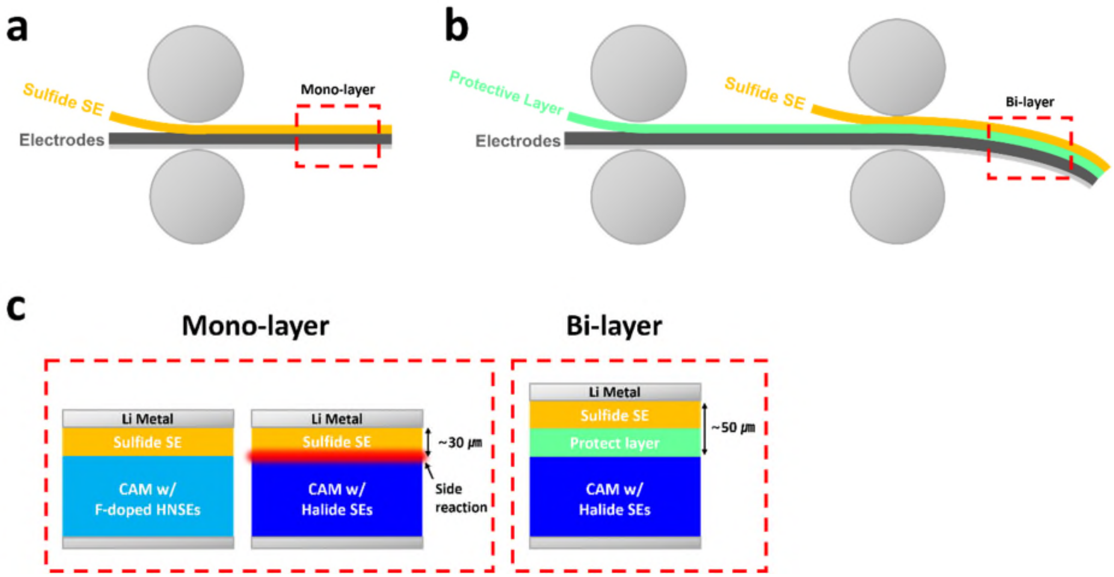

Supplementary Fig. 35 Advantage of ASSB design using sulfide monolayer vs. halide|sulfide bilayer. A,b, Roll-to-roll assembly process of ASSBs using the LPSCl monolayer (**a**) and halide|LPSCl bilayer (**b**). **c**, Schematic of ASSB cells employing a sulfide monolayer or halide|sulfide bilayer. (CAM: cathode active materials)

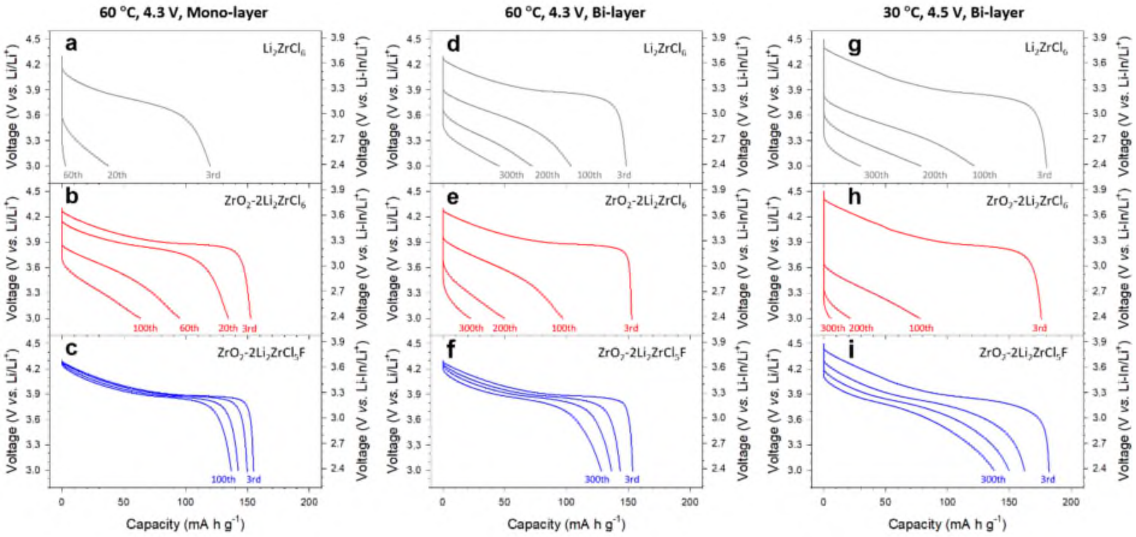

Supplementary Fig. 36 Li–In||LiCoO2 ASSB cells employing halide Ses. A-c, Discharge voltage profiles cycled up to 4.3 V at 60 °C for the cells with LPSCl monolayers using LZC (**a**), ZrO_2-LZC (**b**), and ZrO_2-LZCF (**c**). **d-f**, Discharge voltage profiles cycled up to 4.3 V at 60 °C for the cells with (ZrO_2-LZCF)|LPSCl bilayers using LZC (**d**), ZrO_2-LZC (**e**), and ZrO_2-LZCF (**f**). **g-i**, Discharge voltage profiles cycled up to 4.5 V at 30 °C for the cells with (ZrO_2-LZCF)|LPSCl bilayers using LZC (**g**), ZrO_2-LZC (**h**), and ZrO_2-LZCF (**i**). The specific current was 82.0 mA g^{-1}. The corresponding results are shown in Figure 5c, e, g. The specific current and capacity were determined based on the mass of active material (10.2 mg for LCO and 7.3 mg for S-NCM88). All the cells were cycled under a pressure of 70 MPa.

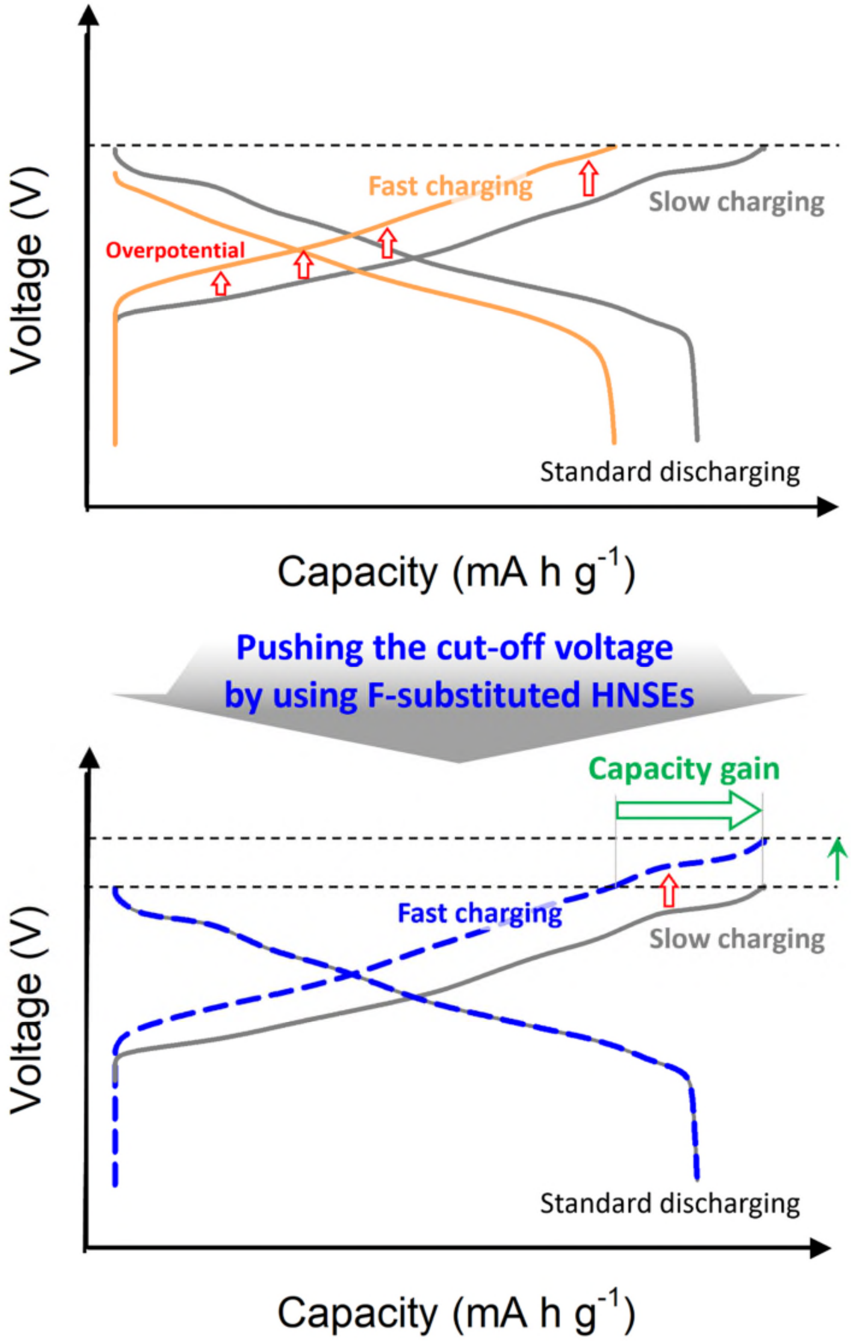

Supplementary Fig. 37 Fast charging enabled by pushing the upper cut-off voltages. Schematic illustrating the enhanced fast-charging ability achieved by raising the upper cut-off voltage, which is enabled by employing high-voltage-stable F-substituted HNSE ZrO_2-LZCF.

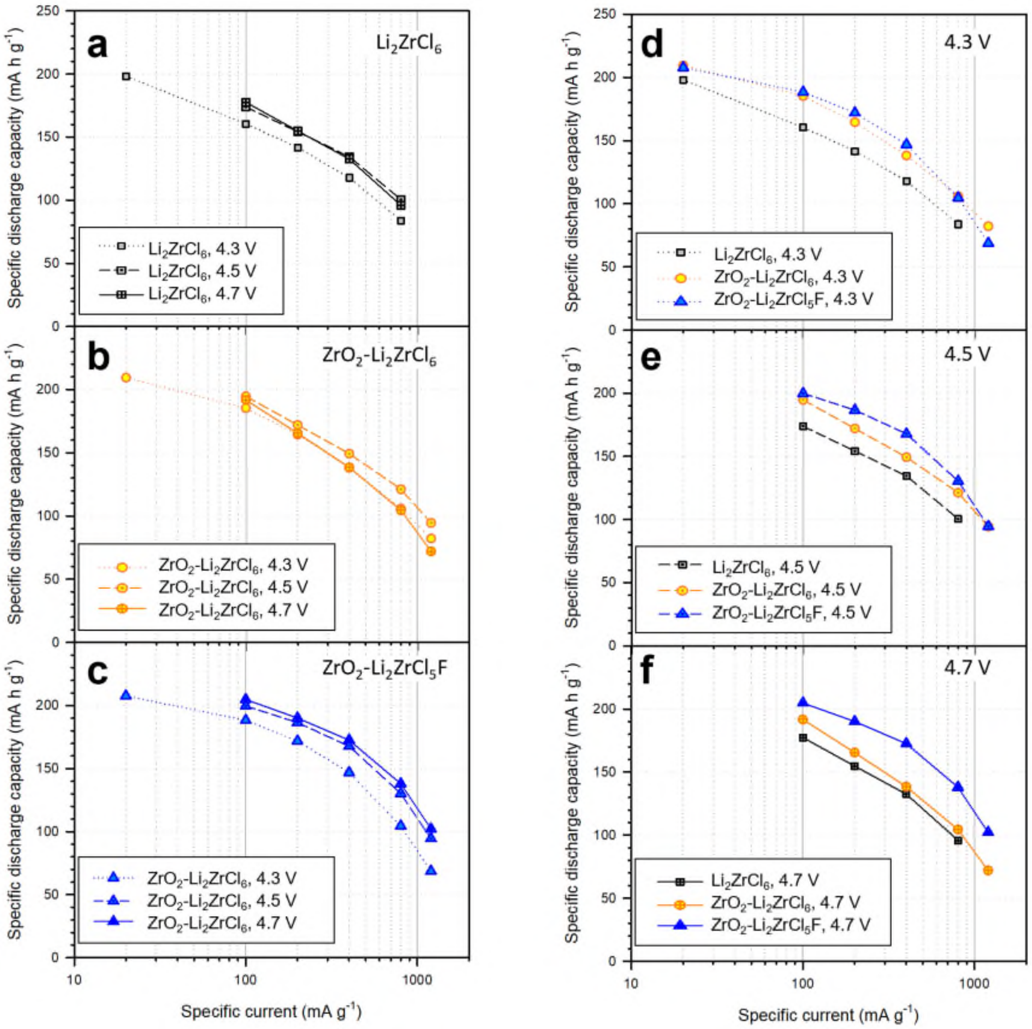

Supplementary Fig. 38 Rate capabilities of Li-In||S-NCM88 ASSB cells at 30 °C. a-c, Discharge capacities as a function of C rates for S-NCM88 electrodes using LZC (**a**), ZrO_2-LZC (**b**) and ZrO_2-LZCF (**c**). **d-f**, Specific discharge capacities as a function of specific current for S-NCM88 electrodes with cut-off voltages of 4.3 V (**d**), 4.5 V (**e**), and 4.7 V (**f**). The corresponding results are shown in Figure 5h. The specific current and capacity were determined based on the mass of active material (7.3 mg for S-NCM88).

Supplementary Table 1 Energy of reactants and products in the HNSE synthesis with the stoichiometric reaction equation of the in situ formation of ZrO_2.

Compound	Energy per formula unit (eV/f.u.)
LiCl	-7.436
$ZrCl_4$	-24.825
Li_2O	-14.347
ZrO_2	-28.511
Li_2ZrCl_6	-39.763

$LiCl + 2ZrCl_4 + 2Li_2O \rightarrow 5LiCl + ZrCl_4 + ZrO_2$ ($\Delta E = -4.736$ eV) (Supplementary equation 1)

$4LiCl + 5ZrCl_4 + 2Li_2O \rightarrow 4Li_2ZrCl_6 + ZrO_2$ ($\Delta E = -5.000$ eV) (Supplementary equation 2)

Supplementary Table 2 Quantitative structural information for Li_2ZrCl_6, ZrO_2-$2Li_2ZrCl_6$, and $n$$ZrO_2$-$2Li_2ZrCl_6$, acquired from coordination-number-refined EXAFS fitting analysis.

Sample	Path	[a] ACN	[b] σ^2 (Å^2)	[c] R (Å)		[d] R-factor (%)	[e] ΔE (eV)
Li_2ZrCl_6	Zr-Cl	1.5	0.00549	2.29693	2.422713 (Average)	0.92	-0.03
	Zr-Cl	4.5	0.00514	2.46464			
ZrO_2-$2Li_2ZrCl_6$	Zr-O	4.52499	0.00112	2.10031		1.48	-0.62
	Zr-Cl	5.56340	0.01060	2.47099			
	Zr-Zr	7	0.00797	3.46418			
$n$$ZrO_2$-$2Li_2ZrCl_6$	Zr-O	5.43812	0.00038	2.10271		0.56	-1.12
	Zr-Cl	5.76121	0.00859	2.46736			
	Zr-Zr	7	0.00431	3.46318			

** a: Average coordination number, b: Debye-Waller factor, c: Bond length, d: EXAFS R-factor, e: Inner shell potential shift*

Supplementary Table 3 Comparison of reliable factors obtained from coordination-number-fixed and refined EXAFS fitting analysis for ZrO_2-Li_2ZrCl_6 and *n*ZrO_2-Li_2ZrCl_6.

	Coordination number fixed fitting		Coordination number refined fitting	
	ZrO_2-2Li_2ZrCl_6	*n*ZrO_2-2Li_2ZrCl_6	ZrO_2-2Li_2ZrCl_6	*n*ZrO_2-2Li_2ZrCl_6
R-factor (%)	2.28	0.84	1.48	0.56
Reduced χ^2	131.04	282.83	85.06	187.76
ΔE (eV)	-0.69	-1.17	-0.62	-1.12

Supplementary Table 4 Fitted results for the EIS data of ion-blocking Ti|SE|Ti symmetric cells at 30 °C shown in Figures 2, 4, and Supplementary Figures 12, 24, and 25. Corresponding equivalent circuit model is presented in Supplementary Figure 10.

HNSE	Thickness (um)	R_1 (Ω)	Q_1 (pF·s$^{(\alpha-1)}$)	α_1	Q_2 (nF·s$^{(\alpha-1)}$)	α_2	χ^2/|Z|	Conductivity at 30°C (mS cm^{-1})	Corresponding Figure no.
Li_2ZrCl_6	640	555.2	83.0	0.958	303	0.922	0.015	0.4	Fig. 2a
ZrO_2-2Li_2ZrCl_6	540	168.1	596	0.804	72.4	0.904	0.040	1.13	
nZrO_2-2Li_2ZrCl_6	510	303.8	-	-	322	0.883	0.246	0.6	
Na_2ZrCl_6	600	18536	53.0	1	81.0	0.914	0.285	0.011	Fig. 2b & Supplementary Fig.12
ZrO_2-2Na_2ZrCl_6	540	3380	126	0.973 7	115	0.974	0.439	0.057	
0.13ZrO_2-0.61NaCl-0.26Na_2ZrCl_6	480	1576	268	0.937	96.6	0.897	1.798	0.11	
$Li_{2.25}Zr_{0.75}Fe_{0.25}Cl_6$	570	209.5	913	0.646	85.4	0.907	0.047	0.96	Supplementary Fig. 24
0.9ZrO_2-2$Li_{2.1}Zr_{0.9}Fe_{0.1}Cl_6$	520	140.8	-	-	46.5	0.940 4	0.096	1.31	
0.75ZrO_2-2$Li_{2.25}Zr_{0.75}Fe_{0.25}Cl_6$	500	126.1	-	-	70.7	0.949 8	0.058	1.40	
0.6ZrO_2-2$Li_{2.4}Zr_{0.6}Fe_{0.4}Cl_6$	520	182	261000	0.464	112	0.906	0.021	1.01	
Li_2ZrCl_5F	610	622.6	268	0.862	40.2	0.921	0.008	0.35	Fig. 4b & Supplementary Fig. 25
$Li_2ZrCl_{4.5}F_{1.5}$	640	790.6	206	0.886	59.1	0.899	0.005	0.29	
$Li_2ZrCl_4F_2$	610	904.7	317	0.867	145	0.914	0.006	0.24	
ZrO_2-2Li_2ZrCl_5F	520	377.6	20.6	1	98.0	0.891 9	0.191	0.49	

Supplementary Table 5 Formulation, fraction, and Li^+ conductivities at 30 °C for HNSEs.

Fraction of precursor (mol.%)			HNSEs	Li^+ conductivity (mS cm^{-1})	Pellet thickness (μm)	Component fraction in HNSEs (vol.%)		
Li_2O	LiCl	$ZrCl_4$				LiCl	ZrO_2	Li_2ZrCl_6
-	66.7	33.3	Li_2ZrCl_6	0.40	630	0.00	0.00	100.00
40.0	-	60.0	ZrO_2-$2Li_2ZrCl_6$	1.17	540	0.00	7.86	92.14
50.0	-	50.0	2LiCl-ZrO_2-Li_2ZrCl_6	1.28	470	21.58	11.43	66.99
66.7	-	33.3	4LiCl-ZrO_2	2.00×10^{-4}	520	79.06	20.94	0.00
35.1	25.1	39.8	1.26LiCl-0.44ZrO_2-0.56Li_2ZrCl_6	1.33	560	24.12	8.89	66.99
24.8	42.1	33.1	1.51LiCl-0.38ZrO_2-0.63Li_2ZrCl_6	1.02	560	26.16	6.86	66.98
12.4	62.8	24.8	2.03LiCl-0.25ZrO_2-0.75Li_2ZrCl_6	0.67	600	29.21	3.81	66.98
-	83.5	16.5	3.06LiCl-Li_2ZrCl_6	0.28	690	33.02	0.00	66.98
26.5	24.5	49.0	0.11LiCl-0.27ZrO_2-0.73Li_2ZrCl_6	1.17	520	2.13	5.74	92.13
9.7	54.2	36.1	0.31LiCl-0.14ZrO_2-0.86Li_2ZrCl_6	1.10	510	5.40	2.47	92.13
-	71.7	28.3	0.53LiCl-Li_2ZrCl_6	0.70	540	7.87	0.00	92.13

Supplementary Table 6 Formulation, fraction, and Na^+ conductivities at 30 °C for HNSEs.

Fraction of precursor (mol.%)			HNSEs	Na^+ conductivity (mS cm^{-1})	Pellet thickness (μm)	Component fraction in HNSEs (vol.%)		
Na_2O	NaCl	$ZrCl_4$				NaCl	ZrO_2	Na_2ZrCl_6
0.0	66.7	33.3	Na_2ZrCl_6	0.011	600	0.0	0.0	100.0
40.0	0.0	60.0	$0.33ZrO_2$-$0.66Na_2ZrCl_6$	0.021	610	0.0	7.0	
25.9	24.8	49.2	$0.24ZrO_2$-0.07NaCl-$0.68Na_2ZrCl_6$	0.040	740	1.99	5.01	
14.6	44.8	40.6	$0.15ZrO_2$-0.16NaCl-$0.69Na_2ZrCl_6$	0.066	810	3.92	3.08	92.9
4.5	32.8	62.7	$0.05ZrO_2$-0.24NaCl-$0.71Na_2ZrCl_6$	0.033	900	5.98	1.02	
0.0	70.6	29.4	0.29NaCl-$0.71Na_2ZrCl_6$	0.014	850	7.0	0.0	
50.0	0.0	50.0	$0.25ZrO_2$-0.5NaCl-$0.25Na_2ZrCl_6$	0.077	510	24.6	9.9	
34.5	25.7	39.8	$0.19ZrO_2$-0.557NaCl-$0.25Na_2ZrCl_6$	0.094	430	27.0	7.5	
20.9	48.2	30.9	$0.13ZrO_2$-0.61NaCl-$0.26Na_2ZrCl_6$	0.109	480	29.5	5.0	65.5
9.6	66.9	23.5	$0.07ZrO_2$-0.67NaCl-$0.26Na_2ZrCl_6$	0.086	470	32.0	2.5	
0.0	82.8	17.2	0.26NaCl-$0.73Na_2ZrCl_6$	0.012	500	34.5	0.0	

Supplementary Table 7 Bond length and volume of octahedra in Li_2ZrCl_6 (LZC) and $Li_{2.5}ZrCl_{5.5}O_{0.5}$ (LZCO). Elongation in the Zr-Cl bond length of LZCO by distortion of the octahedron caused by O-substitution in LZC.

Li_2ZrCl_6 (LZC)		Bond length (Å)	Volume of Octahedron (Å^3)
Zr1	Cl	2.49635<br>2.49635<br>2.49635<br>2.49635<br>2.49635<br>2.49635	20.7412
Zr2	Cl	2.49635<br>2.49635<br>2.49635<br>2.49635<br>2.49635<br>2.49635	20.7412
Zr3	Cl	2.50359<br>2.50359<br>2.50359<br>2.46997<br>2.46997<br>2.46997	20.4463
Zr4	Cl	2.50359<br>2.50359<br>2.50359<br>2.46997<br>2.46997<br>2.46997	20.4463
Zr5	Cl	2.46997<br>2.46997<br>2.46997<br>2.50359<br>2.50359<br>2.50359	20.4463
Zr6	Cl	2.46997<br>2.46997<br>2.46997<br>2.50359<br>2.50359<br>2.50359	20.4463
**Average Bond length of Zr-Cl**		**2.48997**	

$Li_{2.5}ZrCl_{5.5}O_{0.5}$ (LZCO)		Bond length (Å)	Volume of Octahedron (Å^3)
Zr1	Cl	2.46725	20.8752
		2.58657	
		2.58658	
		2.44097	
		2.44098	
		2.50136	
Zr2	Cl	2.48043	20.7755
		2.38707	
		2.38707	
		2.59020	
		2.59020	
		2.58763	
Zr3	O	1.97356	18.2355
		1.97357	
	Cl	2.69480	
		2.46845	
		2.68585	
		2.68586	
Zr4	O	1.90996	19.0874
	Cl	2.51679	
		2.62244	
		2.51678	
		2.59106	
		2.59106	
Zr5	Cl	2.49714	20.7867
		2.49714	
		2.60849	
		2.38230	
		2.51396	
		2.51396	
Zr6	Cl	2.45341	20.6579
		2.45340	
		2.53913	
		2.47819	
		2.52625	
		2.52624	
**Average Bond length of Zr-Cl**		**2.52755**	

Supplementary Table 8 Energy above the hull of O-substituted Li_2ZrCl_6 and Cl-substituted ZrO_2.

Composition	Energy above hull (meV/atom)	Decomposed phase
$Li_7Zr_3Cl_{17}O_1$	37	
$Li_8Zr_3Cl_{16}O_2$	76	$ZrCl_4$, LiCl, ZrO_2
$Li_9Zr_3Cl_{15}O_3$	93	
$Zr_{32}Cl_2O_{62}$	53	
$Zr_{32}Cl_3O_{61}$	80	$ZrCl_2$, ZrO_2
$Zr_{32}Cl_4O_{60}$	93	

Supplementary Table 9 Lattice parameters for Li_2ZrCl_6, ZrO_2-$2Li_2ZrCl_6$, and *n*ZrO_2-$2Li_2ZrCl_6$ calculated from PDF fitting analysis.

Sample	R_w[a] (%)	Lattice parameter									
		Li_2ZrCl_6[d]		ZrO_2[e]				$Li_{2.5}ZrCl_{5.5}O_{0.5}$[f]			Li_2O[g]
		a	*c*	*a*	*b*	*c*	*β*	*a*	*b*	*c*	*a*
Li_2ZrCl_6	17.0	10.97	5.92		-				-		-
ZrO_2	11.6			5.14	5.20	5.31	99.24				-
ZrO_2-$2Li_2ZrCl_6$	10.9[b]<br>16.4[c]	10.96	5.91	5.48	5.83	5.00	103.7	10.97	9.72	15.44	4.79
nZrO_2-$2Li_2ZrCl_6$	13.8	10.96	5.93	5.13	5.21	5.33	99.63		-		-

* a: Reliable factor, b: Reliable factor fitted in 1.5–10 Å, c: Reliable factor fitted in 10–30 Å

* d: $P\bar{3}m1$ (SG# 164), e: $P2/c$ (SG# 13), f: $P1$ (Modeled structure), g: $Fm\bar{3}m$ (SG# 225)

Supplementary Table 10 Reliable factor and mass fraction change obtained from PDF refinement in 1.5 - 10 Å with various compositions of precursors and products for ZrO_2-$2Li_2ZrCl_6$.

Model structure	Mass fraction (%)						R_w[a] (%)
	$Li_6Zr_3Cl_{18}$	Zr_4O_8	$Li_{15}Zr_6Cl_{33}O_3$	Li_8O_4	Zr_2Cl_8	Li_4Cl_4	
Composition_1	82.6	17.4	-	-	-	-	14.6
Composition_2	54.5	13.3	32.2	-	-	-	12.3
**Composition_3**	**53.9**	**14.3**	**31.3**	**0.55**	**-**	**-**	**10.9**
Composition_4	83.6	15.4	-	0.91	-	-	13.9
Composition_5	80.9	14.6	-	-1.2	4.8	0.9	15.8
Composition_6	78.6	13.2	-	-4.0	8.5	-	12.5
Composition_7	89.2	17.2	-	-7.0	-	0.6	13.6

*a: Reliable factor

Supplementary Table 11 Composition of ZrO_2-2Li_2ZrCl_6 and *n*ZrO_2-2Li_2ZrCl_6 calculated from the PDF refinement.

Sample	Composition ratio			
	Li_2ZrCl_6	ZrO_2	$Li_{2.5}ZrCl_{5.5}O_{0.5}$	Li_2O
ZrO_2-2Li_2ZrCl_6	1.47	1.01	0.36	0.16
nZrO_2-2Li_2ZrCl_6	2.05	0.95	-	-

Supplimentary Table 12. The diffusivity corresponding to each temperature obtained through AIMD calculations.

Temperature (K)	Diffusivity of Li^+ ($cm^2\ s^{-1}$)	
	LZC	LZCO
550	1.05×10^{-5}	1.73×10^{-5}
600	1.34×10^{-5}	2.94×10^{-5}
700	3.09×10^{-5}	3.27×10^{-5}
725	3.64×10^{-5}	4.29×10^{-5}
750	4.08×10^{-5}	5.34×10^{-5}
300 (Extrapolated)	8.92×10^{-8}	9.88×10^{-7}

Supplementary Table 13 CV results of Li-In|LPSCl|SE|SE-C cell at 30 °C for LZC and ZrO_2-LZCF.

Upper cutoff voltage (V vs. Li/Li$^+$)	Integrated area (mA V g^{-1})					
	1st			2nd		
	LZC (A)	ZrO_2-LZCF (B)	B/A (%)	LZC (C)	ZrO_2-LZCF (D)	D/C (%)
4.3	0.61	0.5	82.0	0.37	0.07	18.9
4.5	1.04	0.89	85.6	0.63	0.12	19.0
4.7	1.62	1.33	82.1	1.09	0.21	19.3
5.0	2.76	1.98	71.7	2.00	0.55	27.5

Supplementary Table 14 Calculated decomposed voltage and phase equilibria of Li_2ZrCl_6 and Li_2ZrCl_5F.

Composition	Voltage (vs. Li/Li$^+$)	Phase equilibria
Li_2ZrCl_6	0	Zr, LiCl
	1.093	ZrCl, LiCl
	1.293	$ZrCl_2$, LiCl
	1.620	$ZrCl_3$, LiCl
	1.645	Li_2ZrCl_6
	4.307	$ZrCl_4$, Cl_2
Li_2ZrCl_5F	0	Zr, LiCl, LiF
	1.094	ZrCl, LiCl, LiF
	1.245	ZrCl, LiCl, Li_4ZrF_8
	1.288	ZrCl, LiCl, Li_2ZrF_6
	1.293	$ZrCl_2$, LiCl, Li_2ZrF_6
	1.620	$ZrCl_3$, LiCl, Li_2ZrF_6
	1.750	Li_2ZrCl_5F
	4.274	$ZrCl_4$, Cl_2, **Li_2ZrF_6**
	4.341	$ZrCl_4$, Cl_2, **$Li_3Zr_4F_{19}$**
	4.445	$ZrCl_4$, Cl_2, ZrF_4

Supplementary Table 15 Summary of the electrochemical performance of the all-solid-state cells with $LiCoO_2$ and S-NCM88 cathodes

<table>
<tr><th>Positive electrode active material</th><th>Catholyte</th><th>SE layer</th><th>T (°C)</th><th>Upper cut-off voltage (V vs. Li/Li$^+$)</th><th>Specific current (mA g^{-1})</th><th>Capacity[a] (mAh g^{-1})</th><th>Initial coulombic efficiency (%)</th><th>Capacity retention[b] (%)</th><th>Corresponding Figure no.</th></tr>
<tr><td rowspan="14">$LiCoO_2$</td><td>LZC</td><td rowspan="8">Mono-layer</td><td>30</td><td rowspan="11">4.3</td><td rowspan="14">16.4 (2 cycles)-82.0 (after 2 cycles)</td><td>153
149</td><td>93.0</td><td>88.7</td><td rowspan="3">Supplementary Fig. 30</td></tr>
<tr><td>ZrO_2-LZC</td><td>30</td><td>156
152</td><td>95.4</td><td>88.2</td></tr>
<tr><td>ZrO_2-LZCF</td><td>30</td><td>151
146</td><td>95.7</td><td>92.1</td></tr>
<tr><td>LZC</td><td>60</td><td>150
123</td><td>80.3</td><td>1.7</td><td rowspan="3">Fig. 5b,c & Supplementary Fig 29 and 31</td></tr>
<tr><td>ZrO_2-LZC</td><td>60</td><td>152
146</td><td>91.8</td><td>68</td></tr>
<tr><td>ZrO_2-LZCF</td><td>60</td><td>157
155</td><td>94.5</td><td>93.7</td></tr>
<tr><td>Li_3YCl_6</td><td>60</td><td>157
157</td><td>94.4</td><td>72.4</td><td rowspan="2">Supplementary Fig. 31</td></tr>
<tr><td>Li_3InCl_6</td><td>60</td><td>153
146</td><td>88.1</td><td>38.6</td></tr>
<tr><td>LZC</td><td rowspan="6">Bilayer</td><td>60</td><td>155
148</td><td>89.0</td><td>70.0</td><td rowspan="3">Fig. 5d,e & Supplementary Fig. 29</td></tr>
<tr><td>ZrO_2-LZC</td><td>60</td><td>154
153</td><td>92.4</td><td>63.7</td></tr>
<tr><td>ZrO_2-LZCF</td><td>60</td><td>155
154</td><td>94.0</td><td>93.4</td></tr>
<tr><td>LZC</td><td>30</td><td rowspan="3">4.5</td><td>185
180</td><td>92.3</td><td>67.6</td><td rowspan="3">Fig. 5f,g & Supplementary Fig. 29</td></tr>
<tr><td>ZrO_2-LZC</td><td>30</td><td>184
176</td><td>95.0</td><td>44.1</td></tr>
<tr><td>ZrO_2-LZCF</td><td>30</td><td>185
182</td><td>96.1</td><td>89.1</td></tr>
<tr><td>S-NCM88</td><td>ZrO_2-LZCF</td><td>Mono-layer</td><td>30</td><td>4.3</td><td>100 (2 cycles)-400 (after 2 cycles)</td><td>188
140</td><td></td><td>90.8 (3rd/1000th)</td><td>Fig. 5j,k, & Supplementary Fig. 29</td></tr>
</table>

[a] the values in the first and second rows are at the 1st cycle and 3rd cycle, respectively, [b] at the 100th cycle with respect to that at the 3rd cycle; The specific current and capacity were determined based on the mass of active material (10.19 mg for LCO and 7.28 mg for S-

NCM88). Li-In negative electrodes were used in all cells, which were cycled under a pressure of 70 MPa.

Supplementary Table 16 Fitted EIS results of Li–In||$LiCoO_2$ ASSB cells with (ZrO_2-LZCF)|LPSCl bilayers at 60 °C, shown in Supplementary Figure 32. Corresponding equivalent circuit model is presented in Supplementary Figure 10b. R1 represents the bulk resistance of the SE layer. R2 and R3 may represent the interfacial resistance of the composite negative or positive electrode.

Electrode	Cycle number	R_1 (Ω)	R_2 (Ω)	Q_1 ($F\cdot s^{(\alpha-1)}$)	α_1	R_3 (Ω)	Q_2 ($F\cdot s^{(\alpha-1)}$)	α_2	Q_2 ($F\cdot s^{(\alpha-1)}$)	α_2	χ^2/|Z|
Li_2ZrCl_6	2	17.91	2.702	83.2 $\times10^{-6}$	0.998	19.87	0.432 $\times10^{-3}$	0.803	0.0841	0.465	0.021
	10	11.92	2.226	0.0103	0.718	31.85	0.390 $\times10^{-3}$	0.691	0.280	0.560	0.010
	100	12.09	6.873	0.140 $\times10^{-3}$	0.818	105.9	0.296 $\times10^{-3}$	0.749	0.439	0.551	0.009
ZrO_2-2Li_2ZrCl_6	2	17.94	2.2	3.59 $\times10^{-3}$	0.998	33.89	1.49 $\times10^{-3}$	0.618	0.169	0.618	0.011
	10	12.69	4.57	1.74 $\times10^{-3}$	0.692	33.39	0.736 $\times10^{-3}$	0.735	0.413	0.589	0.006
	100	13.95	8.181	0.504 $\times10^{-3}$	0.741	140.5	0.411 $\times10^{-3}$	0.755	0.283	0.459	0.007
ZrO_2-2Li_2ZrCl_5F	2	12.9	2.216	0.102	1	11.96	0.252 $\times10^{-3}$	0.756	0.207	0.547	0.009
	10	14.61	3.446	0.219	0.532	14.01	0.186 $\times10^{-3}$	0.767	0.239	0.528	0.005
	100	13.24	5.175	0.335 $\times10^{-3}$	0.981	9.07	0.108 $\times10^{-3}$	0.837	0.107	0.385	0.067

Supplementary Table 17 Compatibility results by DFT calculations for halide SEs with $LiCoO_2$ and Li_6PS_5Cl.

Mixture		Mutual reaction energies
Cathode material or Sulfide SE	Halide SE	
LCO	Li_2ZrCl_6	-0.40 eV/f.u.
	Li_2ZrCl_5F	-0.37 eV/f.u.
	Li_3YCl_6	-0.18 eV/f.u.
	Li_3YCl_5F	-0.17 eV/f.u.
	Li_3InCl_6	-0.08 eV/f.u.
	Li_3InCl_5F	-0.14 eV/f.u.
LPSCl	Li_2ZrCl_6	-0.92 eV/f.u.
	Li_2ZrCl_5F	-0.85 eV/f.u.
	Li_3YCl_6	-0.46 eV/f.u.
	Li_3YCl_5F	-0.38 eV/f.u.
	Li_3InCl_6	-0.95 eV/f.u.
	Li_3InCl_5F	-1.05 eV/f.u.

Supplementary Note 1 (Regarding Supplementary Figure 2)

Li_2ZrCl_6 is the only stable phase in the ZrO_2-$ZrCl_4$-LiCl ternary region, indicating that the formation of impurity is energetically less favorable than one of Li_2ZrCl_6 and ZrO_2 although local off-stoichiometry may occur during the reaction.

Supplementary Note 2 (Regarding Supplementary Figure 3, 4)

The XRD patterns with increasing ball-milling time are classified into three regions. In the initial stage of the reaction (2, 6, and 8 h, region I), the peaks for LiCl emerge with slightly decreased peak intensities for $ZrCl_4$ and Li_2O. In region II (10, 11, 12, and 16 h), the Li_2ZrCl_6 peaks evolve at the expense of the lowered peak intensities for $ZrCl_4$, Li_2O, and LiCl. Finally, after 20 h (region III), the XRD patterns remained almost identical with the exception of a minor decrease in the peak for Li_2O. Notably, for the sample ball-milled for 30 h, only the Li_2ZrCl_6 peaks are present with no detectable impurity or precursor peaks.

The PDF analysis results show that, as the ball-mill time increases, the signal intensity for Li_2ZrCl_6 increases at the expense of the lowered intensities for Li_2O and $ZrCl_4$ signals. Moreover, the PDF analysis results confirm that the amount of Li_2ZrCl_6 does not increase further after 20 h. In addition, the evolution and increase of the ZrO_2 peak around 2 Å, corresponding to Zr-O bonding, is confirmed during the mechano-chemical milling.

Supplementary Note 3 (Regarding Figure 1e-g)

Upon examining the cryo-TEM images presented in Figures 1e-g, it can be seen that the control sample $nZrO_2$-$2Li_2ZrCl_6$ shows ZrO_2 crystals of several tens of nanometers, while the ZrO_2 synthesized via the reaction of Li_2O and $ZrCl_4$ exhibits smaller crystals of approximately 10-20 nm in size. Assuming that $nZrO_2$ adopts a spherical morphology with a diameter of 40 nm, and that the ZrO_2 in ZrO_2-$2Li_2ZrCl_6$ assumes a spherical shape with a diameter of 20 nm, the specific surface area of $nZrO_2$ can be estimated to be 26.4 $m^2\ g^{-1}$, whereas that of ZrO_2 is 52.8 $m^2\ g^{-1}$. From a semi-quantitative comparison, we can expect that in situ synthesized ZrO_2 possesses a broader interface area.

Supplementary Note 4 (Regarding Supplementary Figure 13)

The oxygen-substituted Li_2ZrCl_6 ($Li_{12+x}Zr_6Cl_{36-x}O_x$ where x = 1-4) structures are prepared by enumeration technique. We considered all possible Cl and O orderings at Cl sites and selected 50 configurations with lowest electrostatic energy. All the structures are fully relaxed with DFT calculation. We checked the channel size of the most stable structure at each composition (Supplementary Figure 13a), and the optimal point with the largest channel size and the highest lattice volume is expected to be x = ≈3 in $Li_{12+x}Zr_6Cl_{36-x}O_x$ ($Li_{15}Zr_6Cl_{33}O_3$). Those are mainly attributed to the lattice expansion effects by oxygen and the lattice contraction effects by lithium, respectively.

Among the generated structures of $Li_{15}Zr_6Cl_{33}O_3$ ($Li_{2.5}ZrCl_{5.5}O_{0.5}$), we selected the structures in which oxygen is placed at one side (since oxygen substitution mainly occurs at the interface of Li_2ZrCl_6 and ZrO_2 and chemical potential difference drives degree of oxygen substitution gradient). For 20 structures, we conducted short AIMD screening during 20 ps at 700 K with topological analysis and the characteristics of structures which show rapid diffusion are analyzed (Supplementary Figure 13b and 13c). As the repulsion between adjacent $ZrCl_{6-x}O_x$ polyhedra increases, the ion conduction channel widens, enabling rapid Li^+ diffusion. Here, when the oxygen ions of neighboring $ZrCl_{6-x}O_x$ polyhedra face each other (agglomerated) in the c-axis direction, such effects are maximized, resulting in fast Li^+ diffusion. Furthermore, the energy difference between the most stable $Li_{2.5}ZrCl_{5.5}O_{0.5}$ structure (O-dispersed) and one with such characteristics (O-agglomerated, Figure 3a) is only 9.4 meV/atom. So, oxygen-agglomerated structures can be sufficiently formed when anion exchange occurs in the interfaces of ZrO_2-Li_2ZrCl_6.

Supplementary Note 5 (Regarding Supplementary Figure 20)

We traced the individual Li^+'s trajectory of the AIMD simulation at 750 K. Although Li^+'s trajectory is localized when Li^+ is moving in an area near oxygen (diffusion in intra-cage), when Li^+ is not in the sphere of influence for interaction with oxygen, it diffuses rapidly through the broadened Li^+ diffusion pathway (diffusion along with extended highway).

Supplementary Note 6 (Considerations about the report of the all-solid-state cell voltage)

Although our cell used a Li-In alloy electrode instead of metallic Li, the cell voltage "vs Li/Li^+" is reported instead of "vs $Li-In/Li^+$". The electrochemical alloying reaction of In with Li^+ proceeds in a series of the intermetallic phases: InLi, In_4Li_5, In_2Li_3, $InLi_2$, $InLi_3$ and In_3Li_{13}.[3,4] In this work, we used $Li_{0.5}In$ (nominal composition), which should consist of In and InLi phases. The corresponding two-phase reaction proceeds with a ~0.62 V (vs. Li/Li^+) plateau. We conducted control experiments using Li||Li and Li-In||Li-In symmetrical cells with a current increment of 0.1 mA cm^{-2} (Supplementary Figure 38). In the specific current density range of 0.5-1.0 mA cm^{-2}, employed in this study of Li-In||$LiCoO_2$ or Li-In||S-NCM88 cells, Li||Li and Li-In||Li-In symmetrical cells exhibited only a marginal difference in overpotential, i.e., <40 mV. Furthermore, Li||Li symmetric cells failed due to a short-circuit at 1.3 mA cm^{-2}, preventing their use for the fair evaluation of positive electrode reversibility. For this reason, we report the voltage of Li/Li^+ as being shifted by 0.62 V from the cell voltage of $Li-In/Li^+$.

Supplementary Note 7 (Regarding Supplementary Figure 32)

Interfacial stabilities between $LiCoO_2$ and SEs while using Li_2ZrCl_6 , ZrO_2-$2Li_2ZrCl_6$ or ZrO_2-$2Li_2ZrCl_5F$ were assessed by EIS measurements. The corresponding Nyquist plots at the second, tenth and one-hundredth cycles are presented in Supplementary Figure 32. The amplitude of semicircles in the Nyquist plots represents the $LiCoO_2$-SE interfacial resistance. Notably, the amplitude of semicircles at the second cycle while utilizing ZrO_2-Li_2ZrCl_5F remained consistent at the tenth and one-hundredth cycles, while the use of Li_2ZrCl_6 or ZrO_2-$2Li_2ZrCl_6$ resulted in a continuous increase in resistance. These results indicate the improved interfacial stability of ZrO_2-Li_2ZrCl_5F.

Supplementary Note 8 (Regarding Supplementary Figure 33)

To investigate the static compatibility of halide and sulfide SEs at 60°C, the following experiment was designed: A pellet was fabricated by mixing halide and sulfide in a 3:1 molar ratio and pressing the mixture at 70 MPa. The pellets were placed into Ti|(halide-LPSCl mixture)|Ti cells, and the ionic conductivity was measured at 60°C immediately after fabrication and after one week of storage at the same temperature. The amplitude of semicircles observed in the Nyquist plots represented the bulk resistance of halide and sulfide SEs, along with the interfacial resistance between halide and sulfide SEs, which could not be deconvoluted. After a week, slight differences were observed in the Nyquist plots (Supplementary Figure 33), providing evidence of limited compatibility between halide and sulfide at 60°C.

Supplementary References

1. Haynes, W. M. Abundance of Elements in the Earth's Crust and in the Sea. *CRC Handbook of Chemistry and Physics, 95th edition, internet Version* (2016).
2. Wang, K. et al. A cost-effective and humidity-tolerant chloride solid electrolyte for lithium batteries. *Nat. Commun.* **12**, 4410 (2021).
3. Jung, Y. S., Lee, K. T., Kim, J. H., Kwon, J. Y. & Oh, S. M. Thermo-electrochemical Activation of an In–Cu Intermetallic Electrode for the Anode in Lithium Secondary Batteries. *Adv. Func. Mater.* **18**, 3010-3017 (2008).
4. Santhosha, A. L., Medenbach, L., Buchheim, J. R. & Adelhelm, P. The Indium−Lithium Electrode in Solid-State Lithium-Ion Batteries: Phase Formation, Redox Potentials, and Interface Stability. *Batt. Supercaps* **2**, 524-529 (2019).
